# Assembling the Dead, Gathering the Living: Radiocarbon Dating and Bayesian Modelling for Copper Age Valencina de la Concepción (Seville, Spain)

**DOI:** 10.1007/s10963-018-9114-2

**Published:** 2018-05-19

**Authors:** Leonardo García Sanjuán, Juan Manuel Vargas Jiménez, Luis Miguel Cáceres Puro, Manuel Eleazar Costa Caramé, Marta Díaz-Guardamino Uribe, Marta Díaz-Zorita Bonilla, Álvaro Fernández Flores, Víctor Hurtado Pérez, Pedro M. López Aldana, Elena Méndez Izquierdo, Ana Pajuelo Pando, Joaquín Rodríguez Vidal, David Wheatley, Christopher Bronk Ramsey, Antonio Delgado-Huertas, Elaine Dunbar, Adrián Mora González, Alex Bayliss, Nancy Beavan, Derek Hamilton, Alasdair Whittle

**Affiliations:** 1 Department of Prehistory and Archaeology, University of Seville, María de Padilla s/n, 41004 Seville, Spain; 2 Valencina de la Concepción Municipal Museum, Plaza de España 9, 41907 Valencina de la Concepción, Seville, Spain; 3 Department of Earth Sciences, University of Huelva, Avda. de las Fuerzas Armadas s/n, 21007 Huelva, Spain; 4 Department of Archaeology and Conservation, Cardiff University, John Percival Building, Colum Drive, Cardiff, CF10 3EU UK; 5 Institut für Ur- und Frühgeschichte und Archäologie des Mittelalters, University of Tübingen, Hölderlinst 12, 72074 Tübingen, Germany; 6 Arqueología y Gestión S.L. Tránsito 8, 41420 Fuentes de Andalucía, Seville, Spain; 7 Research Group Tellus, University of Sevilla, María de Padilla s/n, 41004 Seville, Spain; 8 Department of Archaeology, University of Southampton, Highfield Road, Southampton, SO17 1BF UK; 9 Research Laboratory for Archaeology and the History of Art, Dyson Perrins Building, University of Oxford, South Parks Rd, Oxford, OX1 3QY UK; 10 Andalusian Institute of Earth Sciences, Avda. de las Palmeras 4, 18100 Armilla, Granada Spain; 11 Environmental Research Centre, Scottish Universities, Rankine Avenue, Scottish Enterprise Technology Park, East Kilbride, G75 0QF UK; 12 Historic England, 4th Floor, Cannon Bridge House, 25 Dowgate Hill, London, EC4R 2YA UK; 13 Biological and Environmental Sciences, University of Stirling, Stirling, FK9 4LA UK

**Keywords:** Southern Iberia, Copper Age, Settlement, Mortuary practice, Radiocarbon dating, Formal chronological modelling, Social change

## Abstract

The great site of Valencina de la Concepción, near Seville in the lower Guadalquivir valley of southwest Spain, is presented in the context of debate about the nature of Copper Age society in southern Iberia as a whole. Many aspects of the layout, use, character and development of Valencina remain unclear, just as there are major unresolved questions about the kind of society represented there and in southern Iberia, from the late fourth to the late third millennium cal BC. This paper discusses 178 radiocarbon dates, from 17 excavated sectors within the c. 450 ha site, making it the best dated in later Iberian prehistory as a whole. Dates are modelled in a Bayesian statistical framework. The resulting formal date estimates provide the basis for both a new epistemological approach to the site and a much more detailed narrative of its development than previously available. Beginning in the 32nd century cal BC, a long-lasting tradition of simple, mainly collective and often successive burial was established at the site. Mud-vaulted tholoi appear to belong to the 29th or 28th centuries cal BC; large stone-vaulted tholoi such as La Pastora appear to date later in the sequence. There is plenty of evidence for a wide range of other activity, but no clear sign of permanent, large-scale residence or public buildings or spaces. Results in general support a model of increasingly competitive but ultimately unstable social relations, through various phases of emergence, social competition, display and hierarchisation, and eventual decline, over a period of c. 900 years.

**Electronic supplementary material:**

The online version of this article (10.1007/s10963-018-9114-2) contains supplementary material, which is available to authorized users.

## Introduction

### New Questions for Copper Age Iberia

In the last 20–30 years, research into the Iberian Copper Age has experienced a remarkable upheaval. Numerous new sites have been discovered in the course of development-led fieldwork or through aerial photography, and significant numbers of these have been excavated, substantially augmenting the previously known range of settlements, megalithic tombs and other funerary structures. Interpretations have also been changing. Diffusionist ideas about the supposed Aegean origins of walled settlements and tholos tombs were already unsustainable following the first radiocarbon revolution (Renfrew 1965), and by the 1980s the focus had largely shifted to how social complexity emerged endogenously in Iberia, with discussions typically concentrating on the development of metallurgy, intensification of farming, craft specialisation, and exchange (Chapman 1982, 1990; Harrison 1985; Delibes de Castro et al. 1991; Gilman 1991; Ramos Millán et al. 1991; Monks 1997; Hernando Gonzalo 1997). Although some authors (e.g. Nocete Calvo 2001; López Aldana and Pajuelo Pando 2001, 2011, 2014) have argued for the emergence of high levels of social inequality and even the institutionalisation of power into early ‘state-like’ entities, these views have been challenged (Chapman 2008, p. 248; García Sanjuán and Murillo-Barroso 2013; García Sanjuán et al. 2017).

With continuing discoveries, however, the function and meaning of many of these sites are undergoing another reappraisal. For example, a significant number of ditched enclosures from this period (previously almost unknown in Iberia) have been found and excavated across southern Portugal as well as central and southern Spain. The best-studied so far, Perdigões (Évora), defies any simple categorisation as ‘settlement’ or ‘village’, but instead appears to have operated as a place for periodic or seasonal gatherings in which funerary practices and the manipulation of human remains played a major part (Valera et al. 2014, pp. 24–25). Some stone-walled sites, such as Castanheiro do Vento, in northern Portugal, have been interpreted as monumentalised spaces with largely ritual or funerary functions, and are now labelled simply ‘walled enclosures’ (Jorge 2003).The extension of the geographical spotlight beyond its previous focus on southeast Spain to include southern Portugal, as well as central and southwest Spain, has led to a reassessment of the nature of social relations in that region (Díaz-del-Río 2011; Ramos Millán 2013). It is now clear that there were substantial regional variations in the way Copper Age societies developed in Iberia (Chapman 2008; Balsera Nieto et al. 2015).

This changing debate, however, has not been accompanied by a marked improvement in chronological precision. The Copper Age is usually quoted as spanning c. 3200–2200 cal BC (Chapman 2008; García Sanjuán and Murillo-Barroso 2013), normally on the basis of either visual inspection of radiocarbon dates or of summed probabilities (Balsera Nieto et al. 2015); we are aware of a Bayesian approach to chronology for only one Copper Age megalithic monument (Aranda Jiménez and Lozano Medina 2014) and a ditched enclosure (Balsera Nieto et al. 2015, pp. 151–153). Relatively few radiocarbon dates have been obtained, set against the complexity of the sites involved (Balsera Nieto et al. [2015] list over 600 dates from the Neolithic and Copper Age in southern and central Spain), and short-life, single-entity samples are not yet the default. Increasingly sophisticated interpretation remains based on fuzzy chronology.

This period is often divided into an Early Copper Age and a Late one, the transition marked by the appearance of Bell Beaker pottery in the middle of the third millennium (Garrido Pena et al. 2011), whereas others have recently favoured the traditional tripartite ‘broad periodisation’ (Chapman 2008, p. 235), including an Initial Copper Age c. 3200–3000/2900 cal BC, a Full Copper Age c. 3000/2900–2700/2600 cal BC and a Recent Copper Age c. 2700/2600–2300/2200 cal BC (Chapman 2008, p. 236). Bob Chapman’s survey discusses debates about social differentiation, the character of individual sites and regional variation critically and with insight, but the smallest chronological currency is the scale of a couple of centuries. Subsequently the plausible inference that it is better to think in terms of a kinship mode of production and the short-term mobilisation of labour by emergent chiefs (rather than the development of an early state or any form of political or economic centralisation or class structure), with temporary unity giving way eventually to ‘factionalism, tensions and dispersions’ (Chapman 2008, p. 243), is made without specific reference to even the scale of centuries; what happened might have unfolded over a millennium, and there is no sense of the possible tempo of change. This is *not* to criticise Chapman’s insightful analysis, much of which still stands, but to highlight the type of chronological perspectives which have been and remain common in debates of this kind.

### Valencina de la Concepción

**Fig. 1:**
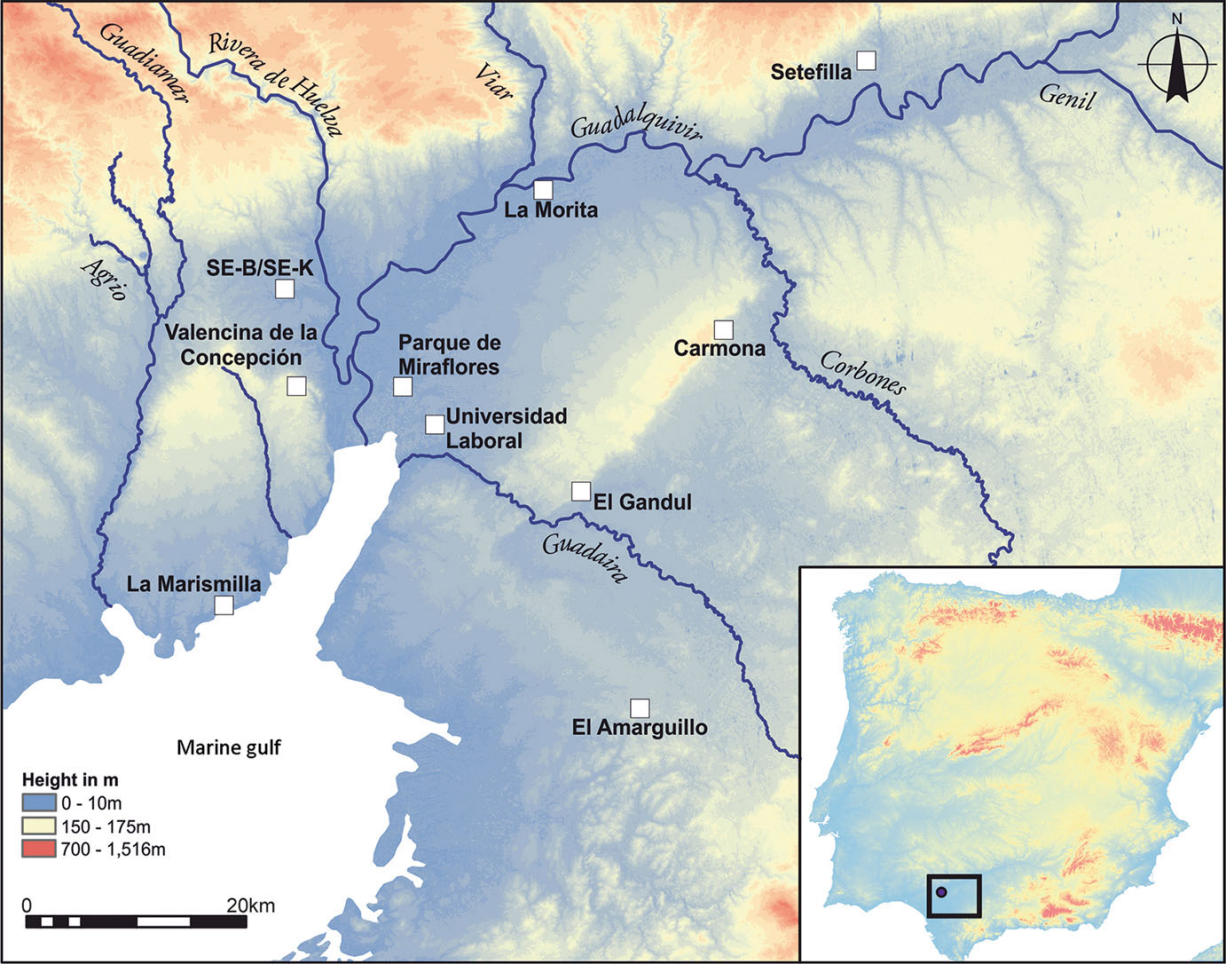
Location map of Valencina de la Concepción and selected other later prehistoric sites of the lower Guadalquivir Valley showing the approximate coastline of the third millennium cal BC. Height data are derived from the ASTER GDEM courtesy of the NASA EOSDIS Land Processes Distributed Active Archive Center (LP DAAC), USGS/Earth Resources Observation and Science (EROS) Center, Sioux Falls, South Dakota. Design: David Wheatley

From all these perspectives, Valencina de la Concepción, located near Seville in the lower Guadalquivir valley, is very important (Fig. 1), the extent and character of the site having emerged gradually. Discoveries in the 19th and earlier 20th centuries, including large tholoi such as La Pastora (Tubino y Oliva 1876) and Matarrubilla (Obermaier 1919; Collantes de Terán 1969; see Gómez de Terreros Guardiola [2005] and Ruiz Moreno [2013] for recent accounts of the early research at the site) were followed from the 1980s by over 120 rescue excavations in advance of urban development; see a synthesis in Vargas Jiménez (2004). This has produced an exceptionally rich record, but the majority of excavations have not been followed by comprehensive post-excavation analysis. In the regional survey discussed above, and following earlier work (cf. especially Vargas Jiménez 2004), Valencina de la Concepción was discussed in terms of separate areas for the living and the dead, with a mooted area of c. 20 ha for settlement, though it was noted that it is difficult to know the extent to which the whole site was occupied ‘at any one time’ (Chapman 2008, p. 240). Nonetheless, it is clear that Valencina differs markedly from smaller, walled sites such as Los Millares (Almagro Basch and Arribas Palau 1963; Molina González and Cámara Serrano 2008) or Zambujal (Sangmeister and Schubart 1981; Kunst 2010). The site is huge, extending over approximately 450 ha, and although there are large ditches, stone architecture appears confined to megalithic monuments and there is no evidence of stratigraphic accumulations outside the negative features that are counted by the thousands (García Sanjuán et al. 2017). Geophysical survey and excavations have revealed scores of pits and shafts, and a range of both small and large megalithic constructions and ‘artificial caves’ (or *hypogea*) (Vargas Jiménez, Meyer and Ortega Gordillo 2012; Wheatley et al. 2012; Mederos Martín et al. 2016; Meyer and Goosens 2016). The site has become central to recent debates about social complexity in Iberia; recent publications include the proceedings of a conference devoted to the site (García Sanjuán, Vargas Jiménez et al. 2013b), a monograph presenting the study of the Montelirio tholos (Fernández Flores et al. 2016), and a host of papers discussing metallurgy, craftsmanship and the exchange of exotic raw materials, notably cinnabar, amber, ivory, gold or rock-crystal (Nocete Calvo et al. 2008, 2013; Costa Caramé et al. 2010; García Sanjuán, Luciañez Triviño et al. 2013a; García Sanjuán and Murillo-Barroso 2013; Rogerio-Candelera et al. 2013; Murillo-Barroso et al. 2015; Morgado Rodríguez et al. 2016).

There are, however, profound unresolved issues in the study of Valencina. A paucity of robust, scientific data about its archaeobotany, archaeozoology and physical anthropology hampers interpretations of its demography, economy and social organisation. Not the least pressing problem is that the temporality of the site is very poorly understood. Before the work presented here, only 40 radiocarbon dates for seven excavated sectors (Cerro de la Cabeza, Plan Parcial Matarrubilla, Avenida de Andalucía Nº 9, IES, Calle Mariana de Pineda s/n, and the Montelirio and La Pastora tholoi) had been published; these span a period from the late fourth millennium cal BC through to the first half of the second millennium cal BC (García Sanjuán 2013, pp. 27–29). This means that we do not know, for example, whether the extraordinary size of Valencina represents a single major focus (of whatever character) or if it is a palimpsest of sporadic, repeated activities over several hundred years. Was it permanently occupied as a village or was it a space for the cyclical aggregation of communities from the surrounding region (or some mixture of the two)? We have no clear sense of the temporal dynamics of the construction, use and closure of the numerous features detected or of the scale of activity at any one moment. Was there any time when the entire 450 ha site was in use? Until we know all this, we cannot make reasonable comparisons with other notable Iberian Copper Age sites such as Los Millares, Zambujal, Camino de Las Yeseras, Perdigões or Marroquíes Bajos, whose detailed chronologies are themselves, in general, far from satisfactory (Table 1) (see various discussions of this topic in Díaz-del-Río 2004; Chapman 2008; Kunst 2010; Cámara Serrano et al. 2012; García Sanjuán 2013; Valera 2013; Valera et al. 2014; Balsera Nieto et al. 2015), and cannot more adequately assess social relations or the scale and tempo of social change.

**Table 1:** Numbers of radiocarbon measurements for significant Iberian Copper Age sites (counts up to October 2015)

Site	Number of ^14^C measurements
Valencina	170 (113 + 57)
Camino de las Yeseras	46
Zambujal	42
Leceia	38
Perdigões	36
Marroquíes Bajos	35
Cabezo Juré	28
Terrera Ventura	26
Los Millares	25
Almizaraque	19
Porto Torrão	14
La Pijotilla	11

### The Radiocarbon Dating Programme and Bayesian Approach at Valencina de la Concepción: A Multi-partner Collaborative Effort

**Fig. 2:**
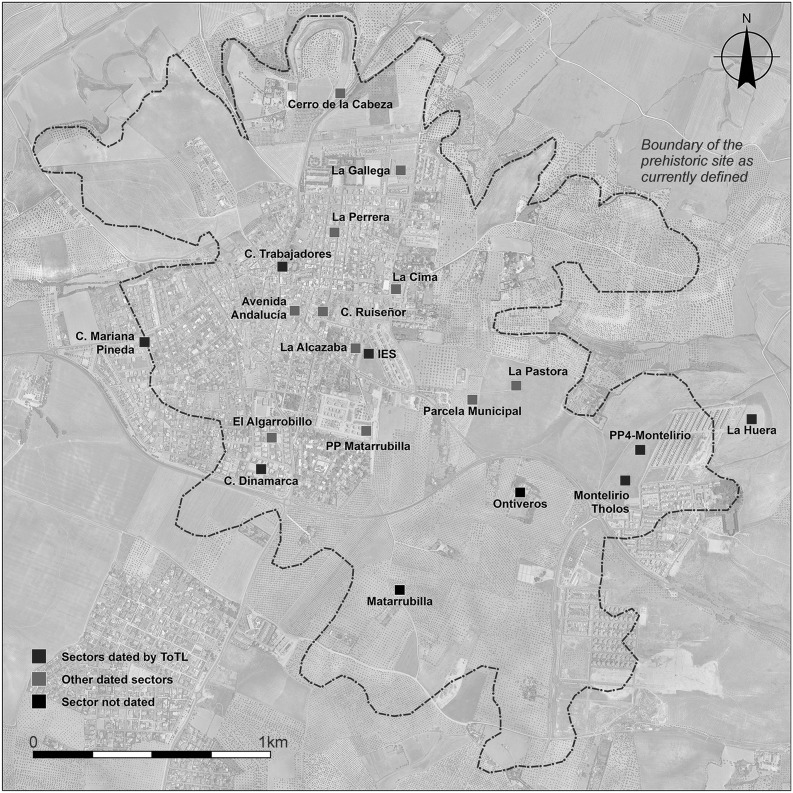
The site of Valencina de la Concepción, showing the locations of the sectors discussed in the text. Aerial photography base map is derived from 1:10,000 aerial photography (1998–2001), Instituto de Estadística y Cartografía de Andalucía. Design: David Wheatley

This paper presents formal chronological models for a selection of mortuary and other contexts from Valencina de la Concepción, blending 30 of the 40 radiocarbon measurements relating to the Copper Age use of the site that have already been published (García Sanjuán 2013, pp. 26–27; Cáceres Puro et al. 2014, Table 1) with a total of 138 new ones, obtained as part of a multi-partner collaborative effort. A total of 29 new measurements were obtained by the University of Seville between 2011 and 2012; eight results were obtained by the University of Huelva in 2014–2015; and 96 new determinations were obtained in the course of a major European Research Council-funded project, *The Times of Their Lives* (ToTL), between 2013 and 2015. (See **Acknowledgements** for further reference to the specific projects involved and their funding institutions.) The 29 measurements obtained by the University of Seville between 2011 and 2012 come from eight different sectors of the site (Calle Ruiseñor, Cerro de la Cabeza, El Algarrobillo, La Alcazaba, La Cima, La Gallega, La Pastora and PP4-Montelirio), and comprise 23 results on human bone and six measurements on unidentified charcoal (Table 2). A further five measurements obtained on ivory samples from the PP4-Montelirio sector are considered invalid due to low levels of collagen (García Sanjuán, Luciañez Triviño et al. 2013a, p. 625; Table 2). Of the eight results obtained in 2014–2015 by the University of Huelva, seven are from the Parcela Municipal Sector (five from unidentified charcoal, one from human bone and one from faunal remains), and one more from a perforated limpet shell bead from La Pastora (Table 2). The 96 measurements obtained by the ToTL project include samples from seven different sectors: La Huera, Calle Dinamarca N^os^ 3–5, Calle Mariana de Pineda, Calle Trabajadores, IES, PP4-Montelirio and Montelirio tholos (Table 3). Figure 2 shows the location of all the newly dated sectors. Altogether, the 138 new dates presented in this paper represent the most intensive effort towards the radiocarbon dating of a later prehistoric site ever carried out in Iberia.

**Table 2:** Radiocarbon dating results for the seven sectors from Valencina de la Concepción dated under the auspices of the Universities of Seville and Huelva

Laboratory number	Context [Sample ID]	Material	δ^13^C (‰)	C:N	Radiocarbon age (BP)
Calle Ruiseñor					
CNA-811	Deposit found inside a ditch that was open during the second phase and in use through the third phase of occupation, in which it was finally abandoned. This deposit represents the final moment in the filling of the ditch [DJ07-51/016/171]	Unidentified charcoal	− 21.99 ± 0.82^†^		4210 ± 35
CNA-812	Deposit corresponding to the hiatus identified between the second and third phases of the stratigraphic sequence [DJ07-51/025/067]	Unidentified charcoal	− 15.62 ± 0.66^†^		4235 ± 35
CNA-815	Deposit found inside a silo that, once its filling had started, was re-used as a burial structure (one individual inhumation) [DJ07-51/061/173]	Unidentified charcoal	− 28.96 ± 0.72^†^		4025 ± 35
CNA-816	Deposit found inside a hut belonging to the third phase of occupation. It corresponds to the final stage of its filling [DJ07-51/070/172]	Unidentified charcoal	− 9.48 ± 0.82^†^		4375 ± 40
CNA-817	Deposit found inside a ditch that was cut in the second phase of occupation. It represents the beginning of the filling of the ditch [DJ07-51/091/225]	Unidentified charcoal	− 26.33 ± 0.60^†^		4430 ± 30
CNA-818	Deposit found above the clay floor of a hut belonging to the first phase of occupation [DJ07-51/094/232]	Unidentified charcoal	− 23.86 ± 0.67^†^		4365 ± 35
Cerro de la Cabeza					
CNA-1277	Structure F1; 11 m deep, 1.1 m wide shaft; found in Unit 124 with marble cylinder idols, ceramics and copper object; female (18–25 years) [FFER18]	Human bone: sphenoid	− 22.27 ± 0.50^†^	3.2	4082 ± 44
CNA-1278	Ditch 1, northwest area grid C-3; − 0.65 m depth; abundant faunal remains and some human skulls commingled; female (18–25 years) [FFER19]	Human bone: parietal	− 14.76 ± 0.82^†^	3.2	4250 ± 31
CNA-1279	Ditch 2; grid 6; female (18–25 years) [FFER20]	Human bone: parietal	− 17.88 ± 0.54^†^	3.2	4230 ± 37
Gif-4028	In Shaft 1. Further description of the context is not available	Unidentified charcoal	Not measured		3910 ± 110
I-10187**	In Shaft 31. Further description of the context is not available	Unknown			4050 ± 105
UGRA-72**	Unknown	Unknown			3380 ± 150
El Algarrobillo					
CNA-1267	Structure 1, grid C-3; southeast sector in first level of human remains; adult male [ALG8]	Human bone: parietal	− 20.84 ± 0.86^†^	3.2	4205 ± 42
CNA-1269	Structure 1, grid C-7; southeast (PROF. 263); adult male [ALG10]	Human bone: parietal	− 21.34 ± 1.02^†^	3.2	4016 ± 26
CNA-1270	Structure 1, grid C-7; southeast (PROF. 263); adult male (26–35 years) [ALG11]	Human bone: occipital	− 22.30 ± 0.66^†^	3.2	3992 ± 39
CNA-1271	Structure 1, grid C-7; northeast; adult male (18–25 years) [ALG12]	Human bone: right temporal	− 24.57 ± 0.65^†^	3.2	4240 ± 44
CNA-1272	Structure 1, grid C-7; adult female (26–35 years) [ALG13]	Human bone: right zygomatic	− 19.27 ± 0.62^†^	3.4	4129 ± 29
CNA-1273	Structure 1, grid C-7; female (26–35 years) [ALG14]	Human bone: parietal	− 22.49 ± 1.13^†^	3.4	3950 ± 25
CNA-1276	Structure 1, grid C-3; adult 26–35 years [ALG17]	Human bone: occipital	− 23.04 ± 0.51^†^	3.2	4239 ± 31
La Alcazaba					
CNA-1260	Inside Structure 19; MNI 7; date from disarticulated commingled remains of four adult individuals [ALC1]	Human bone: right humerus	− 21.91 ± 0.54^†^	3.2	4297 ± 35
CNA-1261	Same context as CNA-1260, adult [ALC2]	Human bone: right humerus	− 18.92 ± 1.00^†^	3.2	4223 ± 32
CNA-1262	Same context as CNA-1260, adult [ALC3]	Human bone: right humerus	− 22.32 ± 0.60^†^	3.3	4252 ± 31
CNA-1263	Same context as CNA-1260, adult [ALC4]	Human bone: right humerus	− 19.84 ± 0.81^†^	3.2	4225 ± 28
La Cima					
CNA-1265	Structure C-6, level 9; articulated skeleton of a subadult (7–13 years)	Human bone: skull	− 18.01 ± 1.12^†^	3.2	4204 ± 49
CNA-1266	Structure C-6, level 9; articulated skeleton of a young adult female (18–25 years)	Human bone: left humerus	− 20.62 ± 0.53^†^	3.2	4257 ± 48
La Gallega					
CNA-1264	Articulated female individual (> 45 years); negative circular Structure 10 and Passage 11	Human bone: occipital	− 24.20 ± 1.05^†^	3.2	3905 ± 35
La Pastora					
CNA-1283	Sample comes from a stratigraphic unit in the passage of the tomb (1991 excavation) [PAS93-24]	Human bone: adult first right metatarsal	− 22.39 ± 0.55^†^	3.1	3929 ± 30
CNA-1284	Corridor of the tomb; 40 m from the tomb chamber (1963 excavation) [PAS64-25]	Human bone: adult skull	− 17.72 ± 0.52^†^	3.2	3999 ± 32
CNA-2504	Perforated shell from a necklace [VA1304] from the 1991 excavation	Shell: limpet	− 1.45 ± 1.50^†^		4280 ± 35
CNA-234	*Petricola lithophaga* shell from calcareous sandstone slab 6 from roof of the passage of the tomb	Shell: *Petricola lithophaga*	− 1.26 ± 0.23^†^		4735 ± 40
CNA-235	*Petricola lithophaga* shell from calcareous sandstone slab 16 from roof of the passage of the tomb	Shell: *Petricola lithophaga*	− 6.84 ± 0.24^†^		4835 ± 35
CNA-236	*Petricola lithophaga* shell from calcareous sandstone slab 6 from roof of the passage of the tomb	Shell: *Petricola lithophaga*	− 3.65 ± 0.25^†^		4520 ± 35
Avenida de Andalucía Nº 9					
UBAR-907	Negative Structure CUE 39, Unit 156 [06/44/156], from a circular bell-shaped feature 2 m in depth which contained an assemblage of bone spindles and awls, arrowheads, ceramic crescents, faunal remains, and a cylindrical betyl	Animal bone	− 20.4		4095 ± 40
UBAR-1024	Ditch 3, Unit 41 [06/44/41], from a concentration of charred material, gastropods, and copper slag from a depth of 1.8 m (the lowest excavated part of the ditch, the base of which was not reached)	Unidentified charcoal	− 31.7 ± 1.2*^†^		3780 ± 60
PP-Matarrubilla					
Ua-19474	MR 2 Cover level of the ditch. Last smelting rubbish dump	Charcoal: *Quercus ilex*	− 24.9		4045 ± 50
Ua-19475	MR 2 Base level of the ditch. First smelting rubbish dump	Charcoal: *Quercus ilex*	− 24.4		4150 ± 50
Ua-22813	Charcoal fragment from within copper slag, NV 18 Furnace	Charcoal: *Quercus ilex*	− 23.4		4050 ± 45
Ua-24557	NV 99 Furnace. Interior of a furnace’s slag	Charcoal: *Quercus ilex*	− 25.3		4135 ± 45
Ua-24558	Charcoal fragment from within copper slag, Furnace 540 NV smelting quarter	Charcoal: *Quercus ilex*	− 22.9		3995 ± 75
Ua-32042	Charcoal fragment from within copper slag, Furnace 182 NV smelting quarter	Charcoal: *Quercus ilex*	− 26.0		4105 ± 40
Ua-32043	Charcoal fragment from within copper slag, NV 104 Furnace.	Charcoal: *Quercus ilex*	− 24.3		3620 ± 55
Ua-32885	Charcoal fragment from within copper slag, IES 14 Furnace.	Charcoal: *Quercus ilex*	− 25.4		4120 ± 40
Ua-36022	Installation NV 77 smelting quarter	Animal bone: *Ovis aries/Capra hircus*	− 18.7		4235 ± 35
Ua-36023	Furnace 104 NV smelting quarter	Animal bone: *Ovis aries/Capra hircus*	− 18.3		3660 ± 55
Ua-36024	Furnace 125 NV smelting quarter	Animal bone: *Ovis aries/Capra hircus*	− 19.7		3985 ± 30
Ua-36025	Charcoal fragment from within copper slag, Furnace 171 NV smelting quarter	Charcoal: *Quercus ilex*	− 19.0		4295 ± 55
Ua-36026	Installation NV 188 smelting quarter	Animal bone: *Ovis aries/Capra hircus*	− 20.4		3965 ± 35
Ua-36027	Furnace 500 NV smelting quarter	Animal bone: *Ovis aries/Capra hircus*	− 19.9		4030 ± 50
Ua-36028	Furnace 505NV smelting quarter	Animal bone: *Ovis aries/Capra hircus*	− 12.7		4105 ± 40
Ua-36029	Charcoal fragment within copper slag from Installation NV506 smelting quarter	Charcoal: *Quercus ilex*	− 16.7		4180 ± 35
Ua-36030	Furnace 508 NV smelting quarter	Animal bone: *Ovis aries/Capra hircus*	− 17.9		4040 ± 35
Ua-36031	Furnace 513 NV smelting quarter	Animal bone: *Ovis aries/Capra hircus*	− 15.7		4010 ± 35
Parcela Municipal					
CNA-1098	Lower level of circular pit number 105 that also contained articulated animal bone [105/412/29]	Unidentified charcoal	− 25.99 ± 0.39^†^		4270 ± 25
CNA-1099	Upper part of the infill of ditch 206 [206/401/32]	Unidentified charcoal	− 25.94 ± 0.40^†^		4135 ± 35
CNA-1100	Lowest stratigraphic unit of feature 54 [54/248/50]	Unidentified charcoal	− 26.69 ± 0.47^†^		4225 ± 45
CNA-1101	Fill of a circular pit, feature 91, that cut upper fill of feature 54 [91/86/29]	Unidentified charcoal	− 26.22 ± 0.32^†^		4100 ± 50
CNA-1496	Infill associated with a concentration of sun-dried mud (358) on top of the western most ditch 186 [186/187/50]	Animal bone	− 19.84 ± 0.88^†^		3978 ± 46
CNA-1497	Lowest stratigraphic unit of feature 54 [54/243/44]	Unidentified charcoal	− 29.05 ± 1.46^†^		4149 ± 31
CNA-1499	Inhumation within structure 435 [435/ROH437]	Human bone	− 19.64 ± 1.13^†^		3967 ± 30

*The UBAR laboratory has indicated that they feel the δ^13^C measurement is too low (Joan Salvador Mestres Torres, pers. comm.)

**Details of these samples that were submitted in the 1970s could not be tracked down, but may still exist somewhere in the site archive in the Archaeology Museum of Seville (Fernando Fernández Gómez, pers. comm.)

^†^δ^13^C value unsuitable for dietary reconstruction

**Table 3:** Radiocarbon dating results for the seven sectors from Valencina de la Concepción that were dated as part of the project, The Times of Their Lives

Laboratory Number	Context [Sample ID]	Material	δ^13^C (‰)	δ^15^N (‰)	C:N	Radiocarbon age (BP)
La Huera						
OxA-30331	Individual 109, context 2229, was part of an assemblage of disarticulated human remains in the central chamber of an artificial cave. The excavator suggests that bodies were brought to the site while still fleshed and then disturbed by later burials. This deposit was possibly closed by the collapse of the chamber roof. The sample is stratigraphically earlier than the disarticulated individuals in deposit 2227, but not stratigraphically related to deposit 2236. Eleven right femurs were found in context 2229 (Individuals 20, 39, 71, 75, 76, 109, 136, 150, 152, 174 and 204). Sex and age: not available (n/a) [2229.109]	Human bone: disarticulated right femur	− 18.5 ± 0.2	9.8 ± 0.3	3.2	4508 ± 30
SUERC-53943	Individual 150, context 2229. See context description for OxA-30331 for details. Sex and age: n/a [2229.150]	Human bone: disarticulated right femur	− 19.8 ± 0.2	9.6 ± 0.3	3.3	4553 ± 31
SUERC-53938	Individual 75, context 2229. See context description for OxA-30331 for details. Sex and age: n/a [2229.75]	Human bone: disarticulated right femur	− 19.6 ± 0.2	8.7 ± 0.3	3.3	4397 ± 29
OxA-30334	Individual 3, context 2236, was found in an assemblage of disarticulated human remains in the central chamber of an artificial cave. The excavator suggests that bodies were brought to the site while still fleshed and then disturbed by later burials. This deposit was closed by the collapse of the chamber roof. The sample is stratigraphically earlier than the disarticulated individuals in deposit 2227, but not stratigraphically related to deposit 2229. Three right femurs were found in context 2236 (individuals 1, 3 and 6). Sex and age: n/a [2236.3]	Human bone: disarticulated right femur	− 19.3 ± 0.2	9.3 ± 0.3	3.1	4429 ± 29
SUERC-53944	Individual 6, context 2236. See context description for OxA-30334 for details Sex and age: n/a [2236.6]	Human bone: disarticulated right femur	− 19.2 ± 0.2	9.3 ± 0.3	3.2	4390 ± 31
SUERC-47677	Inhumation 2201 was fully articulated, and placed in a pit dug into the final fill of the La Huera artificial cave and therefore post-dating the last use of the feature as a burial chamber and the semi-articulated burial 2207. Age 17–22 years. Undetermined sex [2201]	Human bone: articulated left femur	− 19.2 ± 0.2	9.0 ± 0.3	3.2	4259 ± 33
OxA-28234	Inhumation 2207 was partially articulated, and was placed in the central chamber of the artificial cave, after the roof had collapsed and the space for burial had become restricted. The burial pre-dates the closing of the chamber and occurred before burial 2201, but it is stratigraphically later than two fully articulating burials within the chamber of the artificial cave (2209 and 2228). ?Female. 12–15 years [2207]	Human bone: articulated left humerus	− 18.5 ± 0.2	15.1 ± 0.3	3.3	4416 ± 31
SUERC-47678	Inhumation 2209 was placed in the central chamber of the artificial cave, after the roof had collapsed and the space for burial had become restricted. The burial therefore pre-dates the closing of the chamber and is stratigraphically earlier than Individual 2207. The burial, along with 2228, is later than the partial collapse of the chamber roof. Adult.?Female. Less than 25 years [2209]	Human bone: articulated right femur	− 19.5 ± 0.2	10.1 ± 0.3	3.3	4361 ± 31
OxA-28235	Individual 3, context 2227, was found in an assemblage of disarticulated remains in the central chamber of an artificial cave. The excavator suggests that bodies were brought to the site while still fleshed and then disturbed by later burials. This deposit was closed by the collapse of the chamber roof. The sample is stratigraphically earlier than articulated individuals 2209 and 2228 and later than the disarticulated remains in deposits 2229 and 2236. Sex and age: n/a [2227.3a]	Human bone: disarticulated right femur	− 18.1 ± 0.2	9.9 ± 0.3	3.3	4509 ± 30
SUERC-47679	Replicate of OxA-28235 [2227.3b]	Human bone: disarticulated right femur	− 19.2 ± 0.2	8.9 ± 0.3	3.3	4354 ± 33
Mean 2227.3	T′(^14^C) = 12.1; T′(δ^13^C) = 15.1; T′(δ^15^N) = 5.6; T′(5%) = 3.8; ν = 1		− 18.7 ± 0.14	9.4 ± 0.21		4440 ± 23
SUERC-60397	Inhumation 2228 was placed in the central chamber of the artificial cave, after the roof had collapsed and the space for burial had become restricted. The burial therefore pre-dates the closing of the chamber and is stratigraphically earlier than individual 2207. The burial, along with 2209, is later than the partial collapse of the chamber roof. Adult female. Less than 30 years [2228 sample 9a]	Human bone: articulated right femur	− 19.1 ± 0.2	9.3 ± 0.3	3.2	4319 ± 31
OxA-32263	Replicate of SUERC-60397 [2228 sample 9b]	Human bone: articulated right femur	− 18.9 ± 0.2	8.9 ± 0.3	3.3	4380 ± 40
Mean 2228 sample 9	T′(^14^C) = 1.5; T′(δ^13^C) = 0.5; T′(δ^15^N) = 0.9; T′(5%) = 3.8; ν = 1		− 19.0 ± 0.14	9.1 ± 0.21		4342 ± 25
OxA-30333	Individual 136, context 2229. See context description for OxA-30331 for details. Sex and age: n/a [2229.136A]	Human bone: disarticulated right femur	− 19.1 ± 0.2	8.9 ± 0.3	3.2	4429 ± 29
SUERC-53942	Replicate of OxA-30333 [2229.136B]	Human bone: disarticulated right femur	− 19.5 ± 0.2	9.2 ± 0.3	3.3	4374 ± 29
Mean 2229.136	T′(^14^C) = 1.8; T′(δ^13^C) = 2.0; T′(δ^15^N) = 0.5; T′(5%) = 3.8; ν = 1		− 19.3 ± 0.14	9.1 ± 0.21		4402 ± 21
OxA-28238	Individual 152, context 2229. See context description for OxA-30331 for details. Sex and age: n/a [2229.152]	Human bone: disarticulated right femur	− 19.1 ± 0.2	9.9 ± 0.3	3.2	4493 ± 29
OxA-30330	Individual 20, context 2229. See context description for OxA-30331 for details. Sex and age: n/a [2229.20]	Human bone: disarticulated right femur	− 19.3 ± 0.2	8.9 ± 0.3	3.2	4445 ± 29
OxA-28236	Individual 39, context 2229. See context description for OxA-30331 for details. Sex and age: n/a [2229.39]	Human bone: disarticulated right femur	− 18.5 ± 0.2	11.0 ± 0.3	3.2	4493 ± 29
OxA-28237	Replicate of OxA-28236 [2229.39]	Human bone: disarticulated right femur	− 18.8 ± 0.2	8.4 ± 0.3	3.2	4469 ± 30
Mean 2229.39	T′(^14^C) = 0.3; T′(δ^13^C) = 1.1; T′(δ^15^N) = 37.6; T′(5%) = 3.8; ν = 1		− 18.7 ± 0.14	9.7 ± 0.21		4481 ± 21
OxA-30332	Individual 71, context 2229. See context description for OxA-30331 for details. Sex and age: n/a [2229.71A]	Human bone: disarticulated right femur	− 19.2 ± 0.2	8.7 ± 0.3	3.1	4492 ± 28
SUERC-53937	Replicate of OxA-30332 [2229.71B]	Human bone: disarticulated right femur	− 19.6 ± 0.2	8.4 ± 0.3	3.3	4437 ± 29
Mean 2229.71	T′(^14^C) = 1.9; T′(δ^13^C) = 2.0; T′(δ^15^N) = 0.5; T′(5%) = 3.8; ν = 1		− 19.4 ± 0.14	8.6 ± 0.21		4466 ± 21
SUERC-47681	Individual 76, context 2229. See context description for OxA-30331 for details. Sex and age: n/a [2229.76]	Human bone: disarticulated right femur	− 19.0 ± 0.2	9.5 ± 0.3	3.1	4373 ± 33
SUERC-47680	Individual 1, context 2236. See context description for OxA-30334 for details. Sex and age: n/a [2236.1a]	Human bone: disarticulated right femur	− 19.4 ± 0.2	9.2 ± 0.3	3.2	4374 ± 33
OxA-28323	Replicate of SUERC-47680 [2236.1b]	Human bone: disarticulated right femur	− 19.2 ± 0.2	9.3 ± 0.3	3.3	4364 ± 29
Mean 2236.1	T′(^14^C) = 0.1; T′(δ^13^C) = 0.5; T′(δ^15^N) = 0.1; T′(5%) = 3.8; ν = 1		− 19.3 ± 0.14	9.3 ± 0.21		4368 ± 22
Calle Dinamarca N^os^ 3–5						
OxA-32306	Structure 28, Individual 1. Articulating inhumation from layer 13 within the southern niche of the rock-cut structure. The layer is overlain by layers 1 and 12, and underlain by layers 32 and 53. Sex and age: n/a [28 sample 4 (sample 8a)]	Human bone: right tibia	− 18.6 ± 0.2	10.0 ± 0.3	3.4	4423 ± 31
SUERC-60398	Replicate of OxA-32306 [28 sample 4 (sample 8b)]	Human bone: right tibia	− 18.9 ± 0.2	9.7 ± 0.3	3.2	4470 ± 31
OxA-30336	Same individual as OxA-32306 [28.13.1]	Human bone: indeterminate femur	− 19.2 ± 0.2	9.0 ± 0.3	3.2	4429 ± 29
Mean 28.13 Ind. 1	T′(^14^C) = 1.4; T′(δ^13^C) = 4.5; T′(δ^15^N) = 5.9; T′(5%) = 6.0; ν = 2		− 18.9 ± 0.12	9.6 ± 0.17		4440 ± 18
SUERC-53946	Structure 28, Individual 13. Articulating inhumation from layer 32 within a secondary niche beneath the southern niche. This layer is overlain by layers 1 and 13. Sex and age: n/a [28.32.13]	Human bone: left femur	− 19.8 ± 0.2	9.2 ± 0.3	3.3	4388 ± 31
OxA-30339	Structure 28, Individual 4. Articulating inhumation from layer 32 within a secondary niche beneath the southern niche. This layer is overlain by layers 1 and 13. Sex and age: n/a [28.32.4A]	Human bone: left femur	− 19.0 ± 0.2	9.5 ± 0.3	3.2	4449 ± 29
SUERC-53948	Replicate of OxA-30339 [28.32.4B]	Human bone: left femur	− 19.4 ± 0.2	9.9 ± 0.3	3.3	4450 ± 31
Mean 28.32.4	T′(^14^C) = 0.0; T′(δ^13^C) = 2.0; T′(δ^15^N) = 0.9; T′(5%) = 3.8; ν = 1		− 19.2 ± 0.14	9.7 ± 0.21		4449 ± 22
SUERC-47669	Feature 5, semi-articulated inhumation in the lower layer (31) of a small niche off to the southwest of the main chamber. It is thought the remains were initially placed soon after death in the main chamber and moved to the niche after much of the flesh had decomposed. Sex and age: n/a [5.31.101a]	Human bone: indeterminate humerus	− 19.4 ± 0.2	8.2 ± 0.3	3.5	4367 ± 33
OxA-28241	Replicate of SUERC-47669 [5.31.101b]	Human bone: indeterminate humerus	− 18.9 ± 0.2	8.9 ± 0.3	3.2	4486 ± 29
Mean 5.31.101	T′(^14^C) = 7.3; T′(δ^13^C) = 3.1; T′(δ^15^N) = 2.7; T′(5%) = 3.8; ν = 1		− 19.2 ± 0.14	8.6 ± 0.21		4367 ± 33
SUERC-47670	Feature 5, semi-articulated inhumation in the lower layer (31) of a small niche off to the southwest of the main chamber. It is thought the remains were initially placed soon after death in the main chamber and moved to the niche after much of the flesh had decomposed. Sex and age: n/a [5.31.105]	Human bone: indeterminate ulna	− 19.7 ± 0.2	8.0 ± 0.3	3.3	4347 ± 29
OxA-30337	Feature 5, semi-articulated inhumation in the lower layer (31) of a small niche off to the southwest of the main chamber. It is thought the remains were initially placed soon after death in the main chamber and moved to the niche after much of the flesh had decomposed. Sex and age: n/a [5.31.25]	Human bone: indeterminate humerus	− 19.1 ± 0.2	8.4 ± 0.3	3.1	4440 ± 29
SUERC-60399	Replicate of OxA-30337 [5.31.25]	Human bone: indeterminate humerus	− 19.3 ± 0.2	8.4 ± 0.3	3.2	4390 ± 29
Mean 5.31.25	T′(^14^C) = 1.5; T′(δ^13^C) = 0.5; T′(δ^15^N) = 0.0; T′(5%) = 3.8; ν = 1		− 19.2 ± 0.14	8.4 ± 0.21		4415 ± 21
SUERC-47667	Feature 5, fully articulating Individual 1 at the base of layer 60, within the central chamber. Individual 1 is in the same layer as Individuals 2 and 29, but stratigraphically earlier than Individual 3. Female, adult [5.60.1]	Human bone: left femur	− 19.6 ± 0.2	8.7 ± 0.3	3.3	4169 ± 33
OxA-32307	Same individual as SUERC-47667 [5.60.1 tooth]	Human tooth: upper second incisor	− 18.7 ± 0.2	8.9 ± 0.3	3.3	4306 ± 29
Mean 5.60.1	T′(^14^C) = 9.7; T′(δ^13^C) = 10.1; T′(δ^15^N) = 0.2; T′(5%) = 3.8; ν = 1		− 19.2 ± 0.14	8.8 ± 0.21		4247 ± 22
SUERC-47668	Feature 5, fully articulating Individual 2 at the base of layer 60, within the central chamber. Individual 2 is in the same layer as Individuals 1 and 29, but stratigraphically earlier than Individual 3. Male, adult [5.60.2]	Human bone: left femur	− 19.3 ± 0.2	8.2 ± 0.3	3.4	4257 ± 31
OxA-X-2633-40	Same individual as SUERC-47668 [5.60.2 tooth]	Human tooth: upper left second molar	− 19.1 ± 0.2	8.9 ± 0.3	3.2	4359 ± 32
Mean 5.60.2	T′(^14^C) = 5.2; T′(δ^13^C) = 0.5; T′(δ^15^N) = 2.7; T′(5%) = 3.8; ν = 1		− 19.2 ± 0.14	8.6 ± 0.21		4307 ± 23
OxA-30335	Feature 5, semi-articulating Individual 29 at the base of layer 60, within the central chamber. Individual 29 is in the same layer as Individuals 1 and 2, but stratigraphically earlier than Individual 3. Sex and age: n/a [5.60.29]	Human bone: right humerus	− 18.4 ± 0.2	10.4 ± 0.3	3.1	4355 ± 30
OxA-28239	Feature 5, semi-articulating Individual 3 in layer 60, within the central chamber. Individual 3 is stratigraphically later than Individuals 1, 2, and 29, but earlier than Individual 5. Sex and age: n/a [5.60.3]	Human bone: left femur	− 18.9 ± 0.2	9.4 ± 0.3	3.2	4269 ± 28
OxA-28240	Feature 5, semi-articulating Individual 5 at the top of layer 60, within the central chamber. Individual 5 is stratigraphically later than Individual 3. Sex and age: n/a [5.60.5]	Human bone: left femur	− 18.9 ± 0.2	9.7 ± 0.3	3.2	4221 ± 30
OxA-30338	Structure 5, disarticulated human remains from within layer 60, within the central chamber of the rock-cut pit. The sample represents a different individual than samples 5.60.c6 and 5.60.c7B. Sex and age: n/a [5.60.c14]	Human bone: left femur	− 18.5 ± 0.2	10.4 ± 0.3	3.1	4307 ± 29
SUERC-53945	Structure 5, disarticulated human remains from within layer 60, within the central chamber of the rock-cut pit. The sample represents a different individual than samples 5.60.c7B and 5.60.c14. Sex and age: n/a [5.60.c6]	Human bone: indeterminate tibia	− 19.4 ± 0.2	8.7 ± 0.3	3.2	4324 ± 31
SUERC-53947	Structure 5, disarticulated human remains from within layer 60, within the central chamber of the rock-cut pit. The sample represents a different individual than samples 5.60.c6 and 5.60.c14. Sex and age: n/a [5.60.c7B]	Human bone: indeterminate humerus	− 19.2 ± 0.2	8.7 ± 0.3	3.3	4203 ± 31
Calle Mariana de Pineda s/n						
Sac-2216*	Structure 30	Human bone	–19.0^†^			3840 ± 60
OxA-30340	Structure 30, articulating Individual 6E from the fill of Unit 135. This funerary structure overlies ditch Structure 1. There is an MNI of 14 from the funerary deposit, including a number of articulating and semi-articulating human elements. Sex and age: n/a [30.135.6E.A]	Human bone: right femur	− 19.1 ± 0.2	7.9 ± 0.3	3.2	4019 ± 28
SUERC-60400	Structure 30, articulated Individual 16C. The context is the same as OxA-30340. Sex and age: n/a [30.16C]	Human tooth: lower right first molar	− 19.6 ± 0.2	8.5 ± 0.3	3.2	4026 ± 30
OxA-32305	Structure 30, articulated Individual B61. The context is the same as OxA-30340. Sex and age: n/a [30.B61]	Human tooth: lower left molar (probably third)	− 18.5 ± 0.2	9.8 ± 0.3	3.3	4073 ± 29
Sac-2214*	Structure 1, ditch	Animal bone: disarticulated	–19.6^†^			3870 ± 90
SUERC-53952	Structure 1 (ditch), basal deposit (Unit 139). While not found with articulating elements, the condition of bone surface at the epiphysis is very good. Given the highly porous area of the bone, with only a thin cortex/covering that would have degraded rapidly if it entered the ground unprotected by its epiphyseal plate, the bone probably entered the deposit in a semi-fleshed state [1.139.cow]	Animal bone: cattle phalanx	− 19.5 ± 0.2	7.3 ± 0.3	3.2	4008 ± 31
Calle Trabajadores N^os^ 14–18						
SUERC-47671	Individual 10 from the uppermost deposit (Unit 2) in a negative feature (Structure 1). Unit 2 contained both disarticulated human remains and two articulated individuals, as well as some animal bones, and overlaid Units 119 and 120. Adult, sex indeterminate [1.10]	Human bone: radius	− 19.4 ± 0.2	9.4 ± 0.3	3.2	3957 ± 32
SUERC-53955	Disarticulated animal bone from lower deposit (Unit 120) of Structure 1. Unit 120 directly underlies the uppermost Unit 2 [1.120.sheep2]	Animal bone: sheep/goat; left metacarpal	− 19.7 ± 0.2	8.1 ± 0.3	3.2	3956 ± 31
OxA-30342	Disarticulated human remains from the same context as SUERC-47671. It comes from the same cluster of human bone as two other axis vertebrae (1.2.axis2 and 1.2.axis3) and a human skull. Juvenile, sex indeterminate [1.2.axis1]	Human bone: axis	− 18.8 ± 0.2	8.4 ± 0.3	3.2	3925 ± 29
SUERC-60391	Replicate of OxA-30342 [1.2.axis1 replicate]	Human bone: axis	− 19.0 ± 0.2	8.4 ± 0.3	3.2	3922 ± 32
Mean 1.2.axis1	T′(^14^C) = 0.0; T′(δ^13^C) = 0.5; T′(δ^15^N) = 0.0; T′(5%) = 3.8; ν = 1		− 18.9 ± 0.14	8.4 ± 0.21		3924 ± 22
OxA-30341	Disarticulated human remains from the same context as SUERC-47671. It comes from the same cluster of human bone as two other axis vertebrae (1.2.axis1 and 1.2.axis3) and a human skull. Juvenile, sex indeterminate [1.2.axis2A]	Human bone: axis	− 19.5 ± 0.2	9.0 ± 0.3	3.2	3939 ± 29
SUERC-53954	Replicate of OxA-30341 [1.2.axis2B]	Human bone: axis	− 19.3 ± 0.2	9.5 ± 0.3	3.2	3915 ± 31
Mean 1.2.axis2	T′(^14^C) = 0.3; T′(δ^13^C) = 0.5; T′(δ^15^N) = 1.4; T′(5%) = 3.8; ν = 1		− 19.4 ± 0.14	9.3 ± 0.21		3928 ± 22
SUERC-53953	Disarticulated human remains from the same context as SUERC-47671. It comes from the same cluster of human bone as two other axis vertebrae (1.2.axis1 and 1.2.axis2) and a human skull. Adult, sex indeterminate [1.2.axis3]	Human bone: axis	− 19.4 ± 0.2	7.6 ± 0.3	3.2	3955 ± 31
OxA-28242	Individual 5 from the uppermost deposit (Unit 2) in an artificial cave (Feature 1). Unit 2 contained both disarticulated human remains and two articulated individuals, as well as some animal bones, and overlaid Units 119 and 120. Child (aged 6–12 years), sex indeterminate [1.5]	Human bone: tibia	− 20.2 ± 0.2	8.1 ± 0.3	3.3	3904 ± 29
SUERC-47672	Pig ulna found to refit with its corresponding radius during faunal analysis, comes from the uppermost deposit (Unit 2) in a negative feature (Structure 1), which overlay Units 119 and 120 [1.pig ulna]	Animal bone: pig; ulna	− 20.1 ± 0.2	7.2 ± 0.3	3.3	3966 ± 32
OxA-28243	Disarticulated skull (child, aged 9–15 years) from one of a number of skulls that were placed along the edge of the negative feature (Structure 1). It was one of a number of skulls from this layer (Unit 2) and was accompanied by both disarticulated human remains and two articulated individuals [1.G]	Human bone: cranium	− 19.4 ± 0.2	9.0 ± 0.3	3.3	3940 ± 31
OxA-28244	Disarticulated juvenile or young adult skull from one of a number of skulls that were placed along the edge of the negative feature (Structure 1). It was one of a number of skulls from this layer (Unit 2) and was accompanied by both disarticulated human remains and two articulated individuals [1.L]	Human bone: cranium	− 20.4 ± 0.2	10.2 ± 0.3	3.3	3967 ± 29
SUERC-53957	Structure 136, Unit 135. A cranium was the only human bone recovered from the pit and was possibly a formally placed deposit. Sex and age: n/a [136.135.cranium (B)]	Human bone: cranium	− 19.0 ± 0.2	9.2 ± 0.3	3.3	4130 ± 31
OxA-30380	Structure 136, Unit 135. Sample was taken to investigate whether the mortuary practice of single skull burial potentially involves curated skulls [136.135.pig1]	Animal bone: pig	− 19.7 ± 0.2	3.6 ± 0.3	3.3	3965 ± 29
SUERC-53956	Structure 77, Unit 146. Virtually complete (minus mandible) human skull from the uppermost stratigraphic deposit. The structure is c. 50 m from Structures 1 and 90. The skull has been interpreted as a possible closing/formally placed deposit. Sex and age: n/a [77.146.cranium]	Human bone: cranium	− 19.1 ± 0.2	8.9 ± 0.3	3.3	3878 ± 31
OxA-30343	Structure 77, Unit 146. Sample of disarticulating sheep/goat radius from the same unit as a virtually intact single human skull (77.146.cranium). [77.146.sheep1]	Animal bone: sheep/goat; right radius	− 19.6 ± 0.2	7.4 ± 0.3	3.2	3992 ± 29
SUERC-60396	Replicate of OxA-30343 [77.146.sheep1 replicate]	Animal bone: sheep/goat; right radius	− 19.5 ± 0.2	7.8 ± 0.3	3.2	4005 ± 29
Mean 77.146.sheep1	T′(^14^C) = 0.9; T′(δ^13^C) = 0.1; T′(δ^15^N) = 0.9; T′(5%) = 3.8; ν = 1		− 19.6 ± 0.14	7.6 ± 0.21		3999 ± 21
SUERC-53958	Structure 90, Unit 155. This middle stratigraphic unit contained fragments of a human skull and two articulating sheep/goat vertebrae [90.155.animal (B)]	Animal bone: sheep/goat cervical vertebra	− 19.3 ± 0.2	7.7 ± 0.3	3.2	3884 ± 31
OxA-30379	Structure 90, Unit 155. Same context as SUERC-53958. Sex and age: n/a [90.155.human]	Human bone: cranium	− 18.9 ± 0.2	9.6 ± 0.3	3.3	3907 ± 30
OxA-30400	Replicate of OxA-30379	Human bone: cranium	− 18.9 ± 0.2	9.4 ± 0.3	3.3	3889 ± 28
SUERC-60395	Replicate of OxA-30379	Human bone: cranium	− 19.0 ± 0.2	9.5 ± 0.3	3.2	3897 ± 33
Mean 90.155.human	T′(^14^C) = 0.2; T′(δ^13^C) = 0.2; T′(δ^15^N) = 0.2; T′(5%) = 6.0; ν = 2		− 18.9 ± 0.12	9.5 ± 0.17		3897 ± 18
IES						
OxA-28286	Individual 14/728 is from the uppermost deposit in a small circular rock-cut feature that contained human remains. Most of the remains appear to be disarticulated and most were compressed so the anatomical connections could only be identified once excavated, but the torso of this individual was fully articulating. The MNI of the deposit is 10, based on the number of skulls present. Sex and age: n/a [64.1]	Human bone: left humerus	− 19.9 ± 0.2	8.3 ± 0.3	3.3	4201 ± 25
OxA-28285	Feature 402/403 (also known as the ‘ivory workshop’). The ivory was found concentrated in the northern sector of the feature, within an area c. 30 cm in diameter. The fragments possibly represent one episode of ivory working, though it is not known if all the fragments come from the same tusk. The feature also contained copper tools thought to be used for working ivory [402/403]	Ivory fragments	− 21.3 ± 0.2	7.6 ± 0.3	3.3	4072 ± 27
OxA-30382	Same context as OxA-28285 [402/403.b]	Ivory fragments	− 20.2 ± 0.2	9.0 ± 0.3	3.2	4131 ± 30
Mean 402/403	T′(^14^C) = 2.1; T′(δ^13^C) = 15.1; T′(δ^15^N) = 10.9; T′(5%) = 3.8; ν = 1		− 20.8 ± 0.14	8.3 ± 0.21		4099 ± 21
OxA-32308	Individual 4, block 12, which was the uppermost deposit in a small circular rock-cut feature that contained human remains. Most of the remains appear to be disarticulated and most were compressed, so the anatomical connections could only be identified once excavated. Sex and age: n/a [64.12.4]	Human tooth: upper left first molar	− 19.9 ± 0.2	9.8 ± 0.3	3.3	4208 ± 29
SUERC-53962	Structure 64, Block 12. Human skull from amongst remains that appeared to be disarticulated, with most compressed so the anatomical connections could only be identified once blocks of soils were lifted out of the feature and excavated. Sex and age: n/a [64.12.cranium]	Human bone: cranium	− 19.9 ± 0.2	8.1 ± 0.3	3.2	4114 ± 31
OxA-32309	Structure 64, Individual 10, block 13, which was the uppermost deposit in a small circular rock-cut feature that contained human remains. Most of the remains appear to be disarticulated and most were compressed so the anatomical connections could only be identified once excavated. Sex and age: n/a [64.13.10]	Human tooth: upper left second premolar	− 18.6 ± 0.2	8.9 ± 0.3	3.3	4269 ± 31
OxA-30381	Structure 64, Block 13. Human skull from amongst remains that appeared to be disarticulated, with most compressed so the anatomical connections could only be identified once blocks of soils were lifted out of the feature and excavated. Sex and age: n/a [64.13A.cranium]	Human bone: cranium	− 19.0 ± 0.2	9.0 ± 0.3	3.3	4214 ± 30
SUERC-53963	Replicate of OxA-30381 [64.13B.cranium]	Human bone: cranium	− 19.3 ± 0.2	9.4 ± 0.3	3.3	4094 ± 31
Mean 64.13.cranium	T′(^14^C) = 7.7; T′(δ^13^C) = 1.1; T′(δ^15^N) = 0.9; T′(5%) = 3.8; ν = 1		− 19.2 ± 0.14	9.2 ± 0.21		4157 ± 22
SUERC-53964	Structure 64, Block 16. Human skull from amongst remains that appeared to be disarticulated, with most compressed so the anatomical connections could only be identified once blocks of soils were lifted out of the feature and excavated. Sex and age: n/a [64.16.cranium]	Human bone: cranium	− 20.3 ± 0.2	8.0 ± 0.3	3.2	4278 ± 31
SUERC-47676	Individual CR-6, which was the lowermost deposit in a small circular rock-cut feature that contained human remains. Most of the remains appear to be disarticulated and most were compressed so the anatomical connections could only be identified once excavated. The MNI of the deposit is 10, based on the number of skulls present. Sex and age: n/a [64.19.6a]	Human bone: cranium	− 19.5 ± 0.2	8.6 ± 0.3	3.3	4123 ± 33
OxA-28287	Replicate of SUERC-47676 [64.19.6b]	Human bone: cranium	− 20.3 ± 0.2	9.6 ± 0.3	3.3	4177 ± 28
Mean 64.19.6	T′(^14^C) = 1.6; T′(δ^13^C) = 8.0; T′(δ^15^N) = 5.6; T′(5%) = 3.8; ν = 1		− 19.9 ± 0.14	9.1 ± 0.21		4155 ± 22
OxA-30383	Structure 64, Block 19. Human skull from amongst remains that appeared to be disarticulated, with most compressed so the anatomical connections could only be identified once blocks of soils were lifted out of the feature and excavated. Sex and age: n/a [64.19.cranium]	Human bone: cranium	− 19.5 ± 0.2	9.7 ± 0.3	3.4	4164 ± 33
Ua-32886*	I.E.S. 34 smelting quarter	Charcoal: *Quercus ilex*	–24.4			4215 ± 40
Ua-32887*	I.E.S. 223 domestic area	Charcoal: *Quercus ilex*	–21.9			3265 ± 75
Montelirio tholos						
OxA-28245	Inhumation 103 was a complete individual in the main chamber of the megalithic tomb. The burial is from part of a series of burials, all in anatomical position placed directly on top of each other. The anthropologist suggests that the burials were made in quick succession because the addition of a new burial did not disturb the earlier burials (there were no post-mortem fractures and all the skeletons were in anatomical connection). The burial is stratigraphically directly below 110 and above 111. The chamber in which the burial was placed was cut by a tunnel created in the Late Iron Age/Roman period, but this does not appear to have disturbed the burials. Female. Age 25–29 years [103]	Human bone: right femur	− 20.2 ± 0.2	9.2 ± 0.3	3.3	4279 ± 29
OxA-32304	Same individual as OxA-28245 [103.tooth.a (Sample 20A)]	Human tooth: lower right first molar	− 18.6 ± 0.2	10.1 ± 0.3	3.4	4179 ± 29
SUERC-60405	Same individual as OxA-28245 [103.tooth.b]	Human tooth: upper left first molar	− 19.0 ± 0.2	9.8 ± 0.3	3.2	4203 ± 28
Mean 103	T′(^14^C) = 6.5; T′(δ^13^C) = 34.7; T′(δ^15^N) = 4.7; T′(5%) = 6.0; ν = 2		− 19.3 ± 0.12	9.7 ± 0.17		4220 ± 17
OxA-32303	Inhumation 105, in the same feature as OxA-28245. The burial is stratigraphically directly below 104 and above 115.?Female. Age 18–25 years [105.tooth (Sample 24)]	Human tooth: lower left second molar	− 19.1 ± 0.2	9.9 ± 0.3	3.3	4164 ± 36
SUERC-47682	Inhumation 110, in the same feature as OxA-28245. The burial is stratigraphically directly above Individual 103. Female. Age 22–24 years [110]	Human bone: left femur	− 19.0 ± 0.2	9.7 ± 0.3	3.1	4129 ± 33
SUERC-47686	Inhumation 116, in the same feature as OxA-28245. The burial is stratigraphically directly below 111 and above 115. Adult. Undetermined sex [116]	Human bone: right femur	− 19.1 ± 0.2	9.9 ± 0.3	3.2	4168 ± 33
CNA-585*	Main chamber, Individual 102. Female. Age 25–34 years	Human bone: left radius	–20.6 ± 1.0^†^			4250 ± 35
Ua-40803*	Replicate of CNA-585	Human bone: left radius	– 20.4			4165 ± 30
CNA-586*	Same Individual as CNA-585	Human tooth	– 20.31 ± 0.86^†^			4140 ± 50
Mean Main Ind 102	T′(^14^C) = 4.6; T′(5%) = 6.0; ν = 2					4191 ± 21
CNA-587*	Main chamber, Individual 113. The burial is stratigraphically directly below 114. Female. Age 30–35 years	Human bone: right radius	– 21.74 ± 0.74^†^			3950 ± 70
Ua-40804*	Replicate of CNA-587	Human bone: right radius	– 22.6			3862 ± 30
OxA-32302	Same individual as CNA-587	Human tooth: upper right second molar	− 18.4 ± 0.2	10.6 ± 0.3	3.4	4145 ± 29
CNA-588*	Same individual as CNA-587	Human tooth	– 21.38 ± 0.62^†^			4060 ± 40
Mean Main Ind 113	T′(^14^C) = 48.1; T′(5%) = 7.8; ν = 3					
OxA-32301	Inhumation 343, in the same feature as OxA-28245. The body was covered in a shell-beaded garment and is not stratified with the sequence of burials from the tomb. Age 24–32 years. Female [343 (sample 30B)]	Human bone: right ulna	− 19.1 ± 0.2	9.0 ± 0.3	3.3	4168 ± 30
CNA-589*	Main chamber, Unit 166. Discrete lens of ash and charcoal beneath individuals	Unidentified charcoal	– 22.70 ± 0.70			4400 ± 60
Ua-40805*	Main chamber, Unit 166. Discrete lens of ash and charcoal beneath individuals. Same sample as CNA-589	Unidentified charcoal	– 25.5			4086 ± 35
OxA-30439	Inhumation 229 was placed in the corridor of the megalithic tomb, west of a deposit of two ceramic vessels which have been interpreted as altars. The skeleton was placed in a very tightly crouched position. Male. Age 30–40 years [229.teeth]	Human tooth: lower right third molar	− 18.8 ± 0.2	9.8 ± 0.3	3.1	4125 ± 30
OxA-30385	Inhumation 232 is from the corridor of the megalithic tomb, west of a deposit of two ceramic vessels which have been interpreted as altars. The skeleton was placed in a very slightly crouched position and only the legs, part of the torso and parts of the arms were preserved. Age 25–35 years. Undetermined sex [232.teeth]	Human teeth: upper right and lower right 1st incisors	− 18.9 ± 0.2	10.7 ± 0.3	3.2	4151 ± 30
OxA-X-2535-32	Inhumation 273 is located in the corridor of the megalithic tomb. The burial was placed in a pit which is partly covered by a capstone from the corridor, suggesting it pre-dates the construction of the corridor. As the monument’s corridor was dug into a natural hill (rather than covered with a mound), the length of time between burial 273 taking place and the construction of the monument is unknown. This grave was not accompanied by grave goods, so there are no archaeological indications of its date.?Male. Age 35–55 years [273]	Human bone: right humerus	− 18.7 ± 0.2	9.6 ± 0.3	3.3	5802 ± 34
OxA-32200	Unit 76 is a discrete deposit of burnt botanical material, which took place in the corridor of the megalithic tomb, near Individual 232 and the deposit of two ceramic vessels which have been interpreted as altars. [UE76]	Charcoal: unidentified roundwood twig; less than 4 growth rings	− 24.0 ± 0.2			2569 ± 29
Ua-40801*	Small chamber, Unit 80. Age 35–55. Undetermined sex	Human bone	– 19.9			4180 ± 30
Ua-40802*	Small chamber, Unit 88. Age 35–55. Undetermined sex	Human bone	– 21.4			4002 ± 31
PP4-Montelirio						
OxA-32299	Articulated individual from the lowest level of Structure 71. The body is overlain by Individual 2. Young adult. Indeterminate sex [10.071 Individual 1]	Human tooth: upper right third molar	− 19.1 ± 0.2	11.7 ± 0.3	3.4	4222 ± 28
SUERC-60401	Articulated individual from the lowest level of structure 71. The body overlies Individual 1. Young female adult. Age 17–25 years) [10.071 Individual 2]	Human tooth: lower left first molar	− 18.8 ± 0.2	9.7 ± 0.3	3.2	4192 ± 27
OxA-32300	Articulated individual from the uppermost layer in Structure 71. Young adult. Indeterminate sex [10.071 Individual 4]	Human tooth: upper left first molar	− 19.2 ± 0.2	9.5 ± 0.3	3.3	4147 ± 37
OxA-32370	Disarticulated skull from the intermediate phase of use of Structure 71, below Individual 4. Mature adult. Indeterminate sex [10.071 Individual 6]	Human tooth: upper right first incisor	− 18.8 ± 0.2	8.9 ± 0.3	3.3	4277 ± 30
CNA-1291	Unit 211; one of four individuals in main chamber (10.042) [PP4-2]. Adult. Undetermined sex	Human bone: left ulna	− 23.76 ± 0.66^†^		3.5	4161 ± 34
CNA-1303	Unit 211; one of four individuals in main chamber (10.042) [PP4-4]. Aged 18–25 years. Undetermined sex	Human bone: left ulna	− 19.64 ± 1.36^†^		3.2	4277 ± 31
CNA-1300	Structure 10.031, Unit 453; MNI 3, this is Individual 1; male 25–40 years [PP4-12]	Human bone: skull	− 23.04 ± 0.94^†^		3.4	4094 ± 36
CNA-1301	Structure 10.031, Unit 453; MNI 3, this is Individual 2; female 18–25 years [PP4-13]	Human bone: skull	− 20.31 ± 1.54^†^		3.1	4100 ± 68
Erl-17927	Ivory vessel, Stratigraphic Unit 664-1, lower level of Structure 10.049, grave good to the ‘ivory merchant’ (García Sanjuán et al. 2013a, p. 625)	Ivory				2299 ± 68
Erl-17298	Ivory sheath, Stratigraphic Unit 535, upper level of Structure 10.049 (García Sanjuán et al. 2013a, p. 625)	Ivory				2439 ± 58
Erl-17299	Ivory hilt, Stratigraphic Unit 535, upper level of Structure 10.049 (García Sanjuán et al. 2013a, p. 625)	Ivory				3905 ± 74
Erl-17300	Decorated ivory tusk, Stratigraphic Unit 535, upper level of Structure 10.049 (García Sanjuán et al. 2013a, p. 625)	Ivory				1930 ± 57
Erl-17588	Unworked ivory tusk, Stratigraphic Unit 664-1, lower level of Structure 10.049, grave good to the ‘ivory merchant’ (García Sanjuán et al. 2013a, p. 625)	Ivory				2180 ± 55

Where more than one result was available on the same sample, these have been combined, forming a weighted mean (Ward and Wilson 1978). The laboratory numbers denoted by an asterisk (*) indicate radiocarbon results available from the sector, where the material dated was not sampled as part of The Times of Their Lives

^†^δ^13^C value unsuitable for dietary reconstruction

From the outset, the radiocarbon dating programme for Valencina undertaken under the auspices of the ToTL team was conceived within the framework of Bayesian chronological modelling (Buck et al. 1996). This allows the combination of calibrated radiocarbon dates with archaeological prior information using a formal statistical methodology. Since this approach integrates more than one type of information, it provides date estimates that are not only formal but also more robust and precise than those reliant on only one element of the chronological information available about a site (i.e. either the stratigraphy or the scientific dating). Bayesian chronologies are, however, not absolute, but fundamentally interpretative. This means that it is essential to explore and compare alternative models to investigate the reliability of our suggested chronologies.

Further information on the Bayesian approach can be found in Lindley (1985), who provides an accessible introduction to the principles of Bayesian statistics; Buck et al. (1996), who introduce the approach from an archaeological viewpoint; and Bayliss, Bronk Ramsey et al. (2007), who more specifically provide an introduction to building Bayesian chronologies in archaeology.

For the ToTL project it was decided to concentrate principally on contexts with human remains, given the size of the complex as a whole, as one way to begin to investigate chronological patterning and diversity with greater precision. The forms and contexts of mortuary practice at Valencina are very varied, from individual to collective depositions, and from megaliths to ‘artificial caves’, pits and ditches (Vargas Jiménez 2004; Cruz-Auñón Briones and Mejías García 2013; García Sanjuán and Díaz-Zorita Bonilla 2013; Pajuelo Pando et al. 2013); human remains are also often found in contexts, including pits and ditches, whose part in formal mortuary practice requires further research and discussion. Although there are many other relevant lines of evidence, these mortuary data may be particularly significant in helping to tease out patterns and trends in the development of social relations.

Details of further unpublished radiocarbon measurements from eight sectors are provided in Table 2. These derive from a variety of contexts, principally investigated in the course of rescue excavations in advance of development. These encompass a further series of contexts with mortuary and other remains. Samples used for dating are of varying quality, but the information from these other sectors contributes materially both to the construction of a more precise chronology for Valencina de la Concepción and to changing assessment and characterisation of the site as a whole.

### Radiocarbon Dating and Chronological Modelling

Within the ToTL project, a total of 96 radiocarbon measurements have been obtained from 72 samples of human bone and teeth, animal bone and ivory, and charred plant remains (Table 3). For sectors where there are measurements from both ToTL and previous research—Calle Mariana de Pineda s/n, Montelirio tholos, PP4-Montelirio, and IES—all the results are presented in Table 3, but the pre-existing measurements are indicated with an asterisk, since these samples were not selected with Bayesian chronological modelling in mind and may lack the level of reporting set as the minimum standard for this project. There are a total of 82 pre-existing measurements, with details of those from sectors that were not part of the ToTL project given in Table 2. All reported results are conventional radiocarbon ages, corrected for fractionation (Stuiver and Polach 1977).

In 2013–2015, 42 samples of human and animal bone dated by the Scottish Universities Environmental Research Centre (SUERC-) were processed by gelatinisation and ultrafiltration (Brock et al. 2010), and combusted to carbon dioxide, graphitised and dated by Accelerator Mass Spectrometry (hereafter AMS) (Dunbar et al. 2016; Naysmith et al. 2010). Fifty-three measurements were obtained from samples of human bone, animal bone and ivory and one sample of charcoal that were dated by AMS at the Oxford Radiocarbon Accelerator Unit (OxA-). Samples were pre-treated and combusted as described by Brock et al. (2010), graphitised (Dee and Bronk Ramsey 2000) and measured as described by Bronk Ramsey et al. (2004).

The samples submitted by the ToTL project consist almost entirely of human bone (*n* = 64), with a few samples of animal bone (*n* = 6), ivory (*n* = 1), and charcoal (*n* = 1). The human bone was recovered from a range of features, from those which included inhumations that were observed as bodies in articulation (i.e. placed fresh in the burial structure and relatively undisturbed) to those where the bodies appeared to have been initially placed in the structure while fully fleshed but had been disturbed by later activity. There were also cases of individual or multiple skulls or cranial fragments recovered from structures. The breadth of treatment of the human remains necessitated sampling strategies aimed at understanding the timing and temporality of the individual sectors, but also taking into account the specific manner in which the different structures were used in the Copper Age.

As a general rule, the inhumations with observable articulated bone assemblages were interpreted as having been placed fresh into the structure. In cases where a structure appeared to have been subjected to disturbance, probably as the result of human activity in the past, and bodies were moved to the point that articulations were lost, every care was taken to sample the same element (such as the right femur) to remove any concerns that the same individual was sampled more than once. None of the animal bone samples came from an articulated individual. They were either in basal deposits and selected to provide a *terminus post quem* for the overlying burial activity, or were interpreted as freshly deposited, due to the lack of weathering on specific fragile surfaces or articulating groups of bone. The ivory samples were worked ‘chips’ and were selected to provide a date for when the tusks had been harvested, which might not necessarily be the same as when the ivory was being worked. Finally, the charcoal was short-lived and came from a specific burnt deposit, thus functionally related to the formation of the deposit, and providing a robust date for that specific event. Further details are available on each of these samples in Table 3 and in the text.

Forty-five results are available from charcoal, bone and teeth, and shell samples submitted to the Centro Nacional de Aceleradores, Seville (CNA-) for dating by AMS (Tables 2, 3). Samples were pre-treated, graphitised and measured as described by Santos Arévalo et al. (2009).The reported δ^13^C values were measured by AMS.

A bulk charcoal sample was dated at the Centre des Faibles Radioactivités CNRS–Gif-sur-Yvette (Gif-; Table 2). The sample was pre-treated following a standard acid–alkali–acid process, with the sample of CO_2_ measured by gas proportional counting (Delibrias et al. 1966).

The two bone samples dated at the Instituto Tecnológico e Nuclear, Portugal (Sac-; Table 3) underwent collagen extraction using the Longin (1971) method. The δ^13^C values for the samples were determined by isotope ratio mass spectrometry (IRMS) using the CO_2_ from combusted prepared collagen. The radiocarbon measurement was made using liquid scintillation counting after processing the samples in a benzene synthesis line.

A sample of unknown material, probably either bone or bulk charcoal, was dated by gas proportional counting of CO_2_ at Teledyne Isotopes (I-) in the 1970s (Table 2). The laboratory procedures for the time are summarised in Buckley and Valdes-Pages (1981).

Twenty-five samples of both charcoal and bone were processed for radiocarbon dating by AMS at the Tandem Laboratory, University of Uppsala (Ua-; Tables 2, 3). Samples were prepared as described by Wohlfarth and Possnert (2000), graphitised as described by Vogel et al. (1984) and dated by AMS (Possnert 1984, 1990). The reported δ^13^C values were measured by IRMS (Olsson and Possnert 1992).

One sample of bone and one of charcoal were dated at the University of Barcelona (UBAR-; Table 2). The bone sample underwent collagen extraction as gelatin by means of an acid hydrolysis, after elimination of the bone mineral fraction with hydrochloric acid. The radiocarbon was then measured by liquid scintillation on benzene and the δ^13^C value was obtained by IRMS. The charcoal sample underwent initial mechanical separation and the coarse and fine charcoal fractions were treated separately with hydrochloric acid. The fine fraction was further treated with hydrofluoric acid to eliminate silica. Both fractions were then treated with ammonia and hydrochloric acid prior to submission for graphitisation and measurement of both the radiocarbon age and δ^13^C value by AMS at the Centro Nacional de Aceleradores, Seville (CNA-).

The bone sample dated at the University of Granada (UGRA-; Table 2) was processed following the Longin (1971) method and measured by liquid scintillation spectrometry and reported following González-Gómez et al. (1982).

At Erlangen, the five ivory samples were cleaned and crushed and then treated with acid–alkali–acid, before the collagen was dissolved in acid, dried and separated by centrifugation. Combustion and graphitisation were undertaken as described by Kretschmer et al. (1997) and the samples were dated by AMS as outlined in Kretschmer et al. (1998).

There are 21 sets of replicate radiocarbon measurements (17 pairs, three samples with three measurements, and one with four), either on the same bone sample (*n* = 15) or from the same individual (*n* = 6). Of these replicate groups, 14 are statistically consistent at 2σ, two are statistically consistent at 3σ and five are statistically inconsistent at more than 3σ (Table 3; Ward and Wilson 1978). This scatter is more than would be expected on purely statistical grounds and, within the groups that are statistically inconsistent at more than 3σ in particular, it is likely that some samples have not been accurately dated. Replicate measurements that are statistically consistent at 3σ were combined before calibration and incorporation in the chronological modelling by taking a weighted mean (but see discussion on the results from Individual 103 in the Montelirio tholos below). These means are given in Table 3, along with the relevant results for the test statistics of Ward and Wilson (1978). The accuracy of the measurements in the replicate groups which are statistically inconsistent at more than 3σ was assessed on a case-by-case basis during the modelling process (see further below).

Carbon and nitrogen stable isotopic ratios were measured by IRMS from all bone samples dated as part of the ToTL project (Table 3), as outlined by Brock et al. (2010; OxA-) and Sayle et al. (2014; SUERC-). There are 19 replicate groups of stable isotopic measurements. Fourteen of the groups of δ^13^C values are statistically consistent at 2σ, one is consistent at 3σ, and the remaining four are statistically inconsistent at more than 3σ (Table 3); 14 of the groups of δ^15^N values are also statistically consistent at 2σ, with three more consistent at 3σ, and the remaining two groups statistically inconsistent at more than 3σ (Table 3). These measurements should reflect the natural isotopic composition of the dated sample and so are suitable for assessing past diet (see below).

Measured δ^13^C values are available for most of the pre-existing dated samples (Tables 2, 3). The values reported by Uppsala (Ua-) were measured by IRMS from sub-samples of the dated material and so should also reflect the natural isotopic composition of the dated material. Those reported by Seville and Barcelona (CNA-, UBAR-) were measured by AMS and so may reflect fractionation inherent in the graphitisation and measurement processes as well as the natural isotopic composition; and those reported by the Portuguese laboratory (Sac-) were made on sub-samples of the carbon dioxide produced for conventional dating and so may include a component of fractionation from the open combustion process used. For these reasons, these measurements (marked † in Tables 2, 3) are not suitable for dietary reconstruction.

The chronological modelling presented in this paper has been undertaken using OxCal 4.2 (Bronk Ramsey 1995, 2009a, b), and the internationally agreed calibration curve for the northern hemisphere (IntCal13; Reimer et al. 2013). The models are defined by the OxCal CQL2 keywords and by the brackets on the left-hand side of the graphs included in this paper. In the diagrams, calibrated radiocarbon dates are shown in outline and the posterior density estimates produced by the chronological modelling are shown in solid black. The Highest Posterior Density intervals which describe the posterior distributions are given in italics.

## Sectors Dated by the ToTL Project: Archaeological Description and Chronological Models

For the ToTL project, samples were selected from contexts with human remains across seven sectors of the complex (Table 3). Sectors are presented in chronological order, as far as that can be determined.

### La Huera

**Fig. 3:**
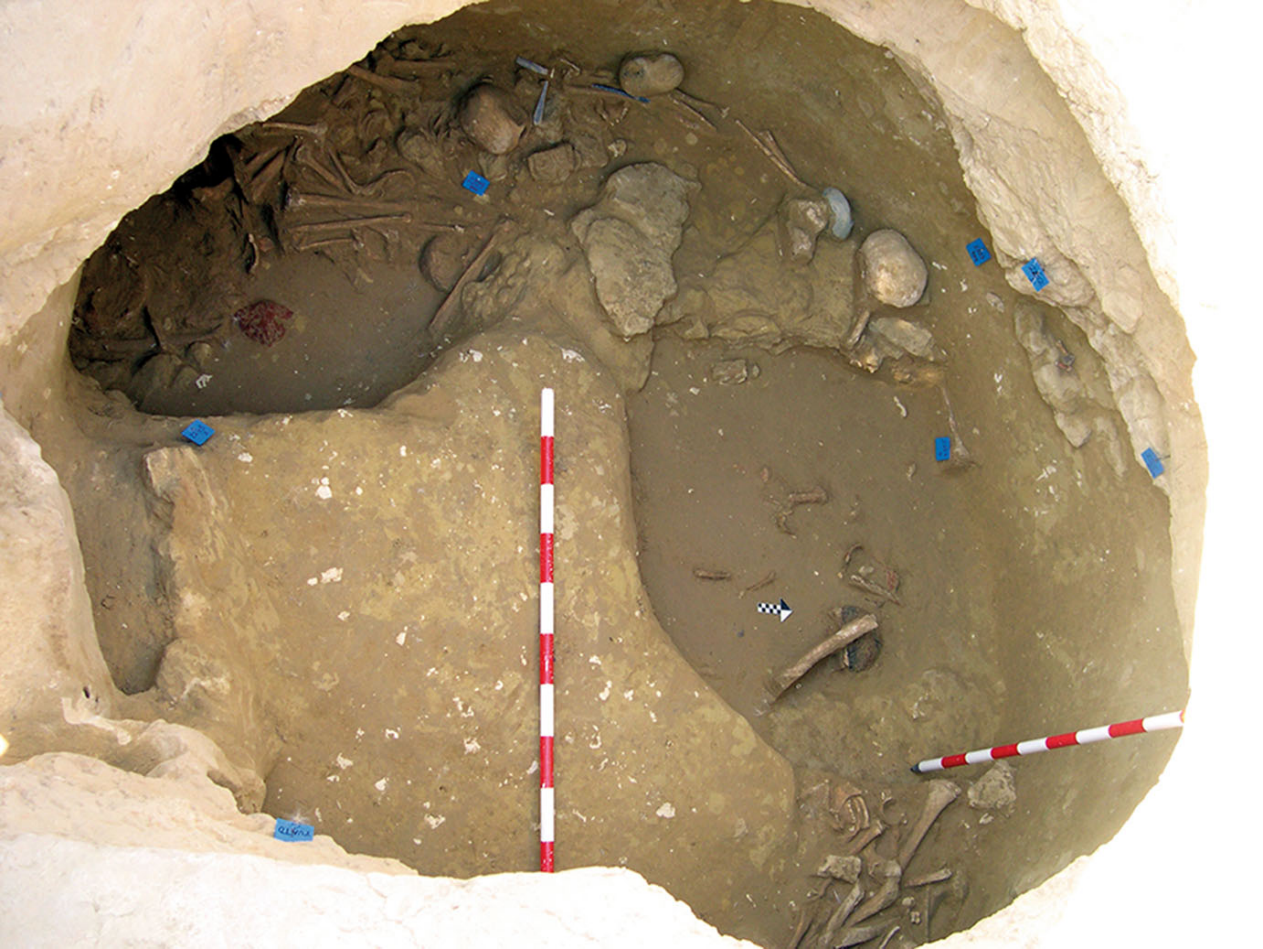
La Huera burial chamber with human bone deposits UU.EE. 2229 and 2236, as well as roof collapse level U.E. 2218. Photo: Elena Méndez Izquierdo

The La Huera artificial cave, on the highest elevation in its locality on the eastern edge of Valencina, was excavated in 2007, as part of a larger intervention covering 25 ha in which other probable Copper Age features, including a pit, and two further probable artificial caves, were discovered but not excavated (Méndez Izquierdo 2010, 2013). The excavated hypogeum comprised a simple, more or less circular, chamber, 2.8 m in maximum diameter and 2.1 m deep, and a small corridor on its southeast side (Fig. 3). We do not know how the structure was roofed. Inside the chamber there were over 300 human bones, mostly disarticulated except for three inhumations that showed complete or partial anatomical articulation. Altogether, the minimum number of individuals (MNI) constituted by these human remains is 22, with a representation of both sexes and an age range from infancy to 50 years, with the most frequent age category being 25–35-year-olds (Méndez Izquierdo 2013, pp. 310–312). Successive individual inhumations were deposited in the lower deposits over a period long enough to cause earlier remains to be displaced towards the sides of the chamber by the introduction of successors. After a period of continued use, the structure was closed. Later on, after an unspecified time interval, a further individual inhumation was made. Material, principally from the upper part of the lower deposits, included five complete or semi-complete pots and as many fragments with typically Copper Age forms, flint blades and arrowheads, polished stones, unworked granite and quartzite, and two rock-crystal cores. There were four bone pins and a small number of faunal remains, including a scallop shell (*Pecten maximus*).

#### Results and Models

This artificial cave has been shown to have a complex history of use with earlier burials being disturbed during episodes of reuse, but the vertical nature of many of the deposits does lend itself to relative ordering of some deposits and those skeletal remains that were found in articulation. There are two deposits of human bone at the base of the cave (2229 and 2236), and many of these burials were disarticulated, though interpreted as having been deposited while still fleshed and then disturbed by later burials. From these two deposits 12 right femurs were selected for dating, thus representing 12 different individuals. Of the 12 individuals, three (2229 Ind. 71, 2229 Ind. 136 and 2236 Ind. 1) had samples dated by both Oxford and SUERC, while the fourth (2229 Ind. 39) has two replicate measurements made at the Oxford laboratory as part of its internal quality assurance protocols. All four replicate groups are statistically consistent, and so weighted means have been calculated and incorporated in the chronological modelling (means: 2229.39, 2229.71, 2229.136 and 2236.1; Table 3). A total of 16 radiocarbon measurements are available from this lowest deposit. Above these two deposits was 2227, in which there were three discrete burials that were disturbed by later activity. There are two results (OxA-28235 and SUERC-47679) from 2227 Individual 3, which are divergent at well over 3σ (T′ = 12.1; T′ (5%) = 3.8; ν = 1; Table 3). This is more than would be expected simply on the statistical scatter of radiocarbon results, and it seems that there must be a problem with one of them. In the initial model, each has been included separately. After an episode of roof collapse that restricted the space for burial, the semi-articulated Inhumations 2209 and 2228 were placed in the chamber. There are two statistically consistent results from Inhumation 2228 (OxA-32263 and SUERC-60397), and so their weighted mean has been used in the model (mean: 2228 sample 9; Table 3). After these two inhumations, the semi-articulated Inhumation 2207 was placed into the structure. The burial structure went out of use and, after a period of time in which the pit was allowed to completely fill, a pit was dug into the upper fills for the deposition of the fully articulated Inhumation 2201.

The modelling for the artificial cave at La Huera uses the 23 radiocarbon results from these 17 individuals, and the observed stratigraphic relationships to develop the chronological framework for the activity described.

The initial model for La Huera has poor agreement between the calibrated radiocarbon dates and the stratigraphy (Amodel: 23; model not shown). Both measurements from Inhumation 2228 have poor individual agreement, although that for OxA-28235 (A: 6) is much lower than that for SUERC-47679 (A: 56). It appears that OxA-28235 is anomalously old, and so it has been excluded and the model re-calculated.

**Fig. 4:**
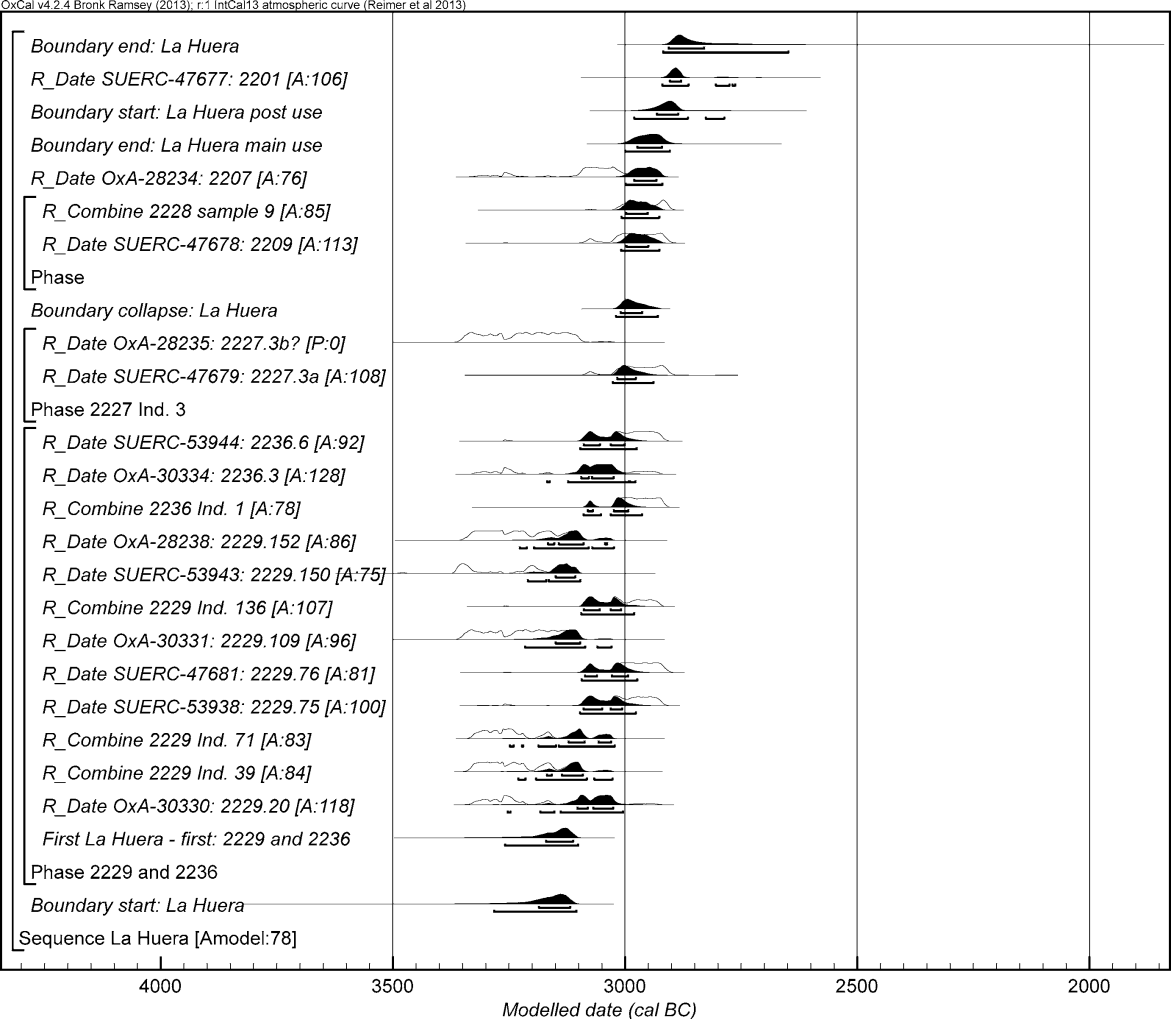
Chronological model for burial activity at La Huera. Each distribution represents the relative probability that an event occurred at some particular time. For each of the radiocarbon measurements two distributions have been plotted, one in outline which is the result of simple radiocarbon calibration, and a solid one which is based on the chronological model use. The other distributions correspond to aspects of the model. For example, ‘*La Huera*—*first: 2229 and 2236’* is the estimated date that the burial began in this sector. The large square ‘brackets’ down the left-hand side along with the OxCal keywords define the overall model exactly

**Fig. 5:**
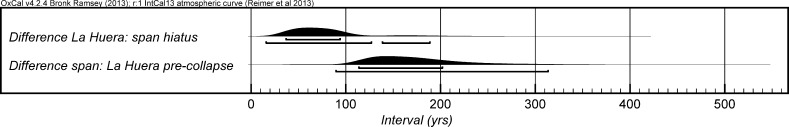
Probability distributions for the number of years over which the majority of burial activity at La Huera took place (*span: La Huera pre*-*collapse*) and number of years (*La Huera: span hiatus*) between *OxA*-*28234: 2207* at the top of the main use sequence and the insertion of *SUERC*-*47677: 2201*. The distributions are derived from the model defined in Fig. 4

This model has good overall agreement between the calibrated radiocarbon dates and the stratigraphic sequence (Amodel: 78; Fig. 4). It estimates that the earliest burial in the structure at La Huera took place in *3260*–*3100* *cal BC* (*95%* *probability*; *La Huera*–*first: 2229 and 2236*; Fig. 4), probably in *3170*–*3100* *cal BC* (*68%* *probability*). The burials in these lower two units (2229 and 2236) ended in *3020*–*2925* *cal BC* (*95%* *probability*; *collapse: La Huera*; Fig. 4), probably in *3010*–*2960* *cal BC* (*68%* *probability*), when the roof collapsed. The overall span of burials in these lowest deposits covered a period of *85*–*315* *years* (*95%* *probability*; *span: La Huera pre*-*collapse*; Fig. 5), probably *110*–*205* *years* (*68%* *probability*).

Burial continued with 2207, 2209, and 2228 being inserted after the roof collapse and before *3000*–*2900* *cal BC* (*95%* *probability*; *end: La Huera main use*; Fig. 4), probably before *2975*–*2920* *cal BC* (*68%* *probability*).

There was no dated activity at the structure for either *15*–*130* *years* (*68%* *probability*; *La Huera span: hiatus*; Fig. 5) or *135*–*190* *years* (*6%* *probability*), probably for *35*–*95* *years* (*68%* *probability*).

Burial 2201 was inserted into a pit in the top of the artificial cave in either *2920*–*2860* *cal BC* (*88%* *probability*; *SUERC*-*47677: 2201*; Fig. 4) or *2805*–*2760* *cal BC* (*7%* *probability*), probably in *2905*–*2875* *cal BC* (*68%* *probability*).

### Calle Dinamarca N^os^ 3–5

**Fig. 6:**
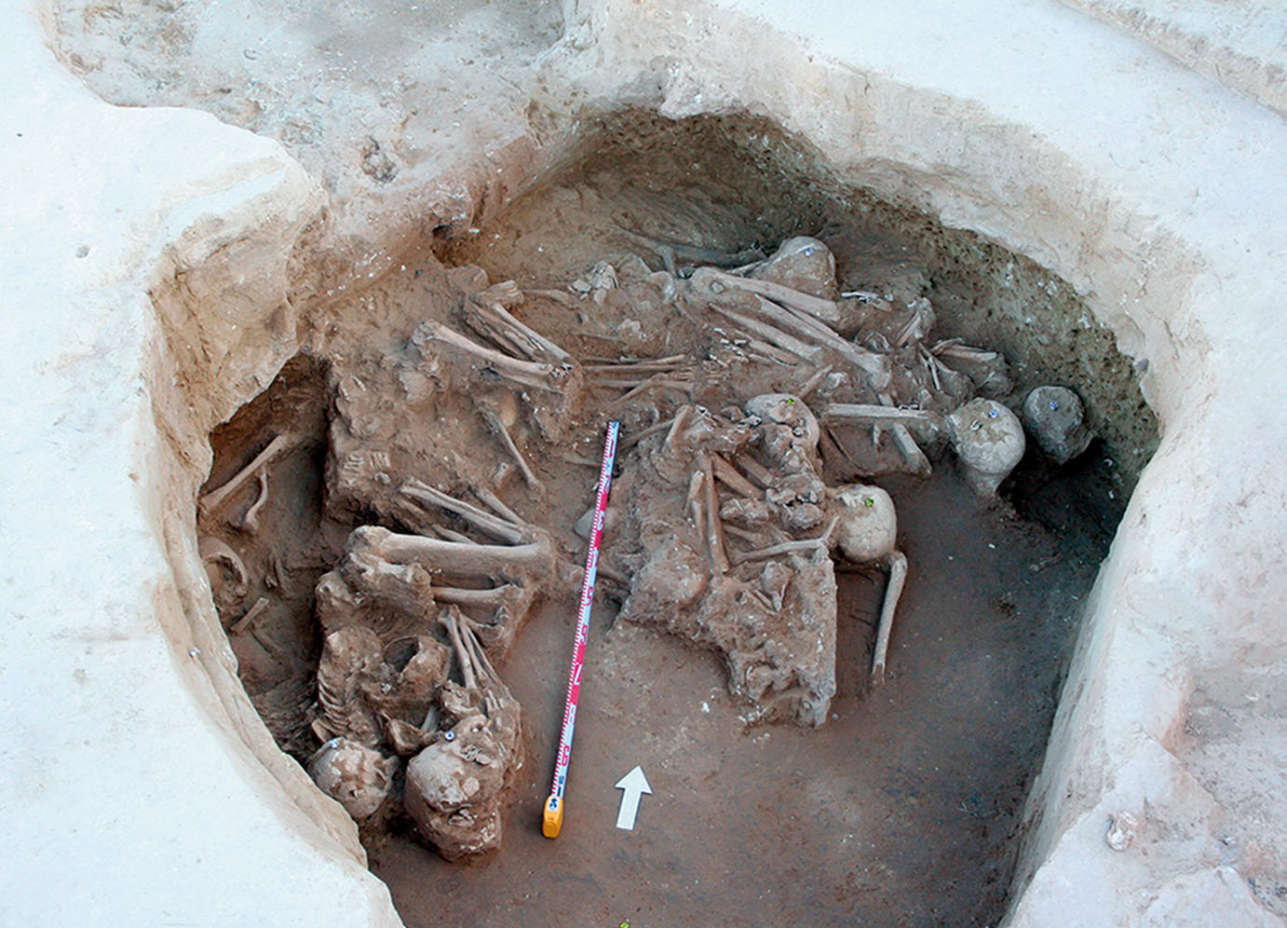
Structure 5 at Calle Dinamarca N^os^ 3–5. General view of the upper layers of the central chamber. Photograph: Ana Pajuelo Pando

**Fig. 7:**
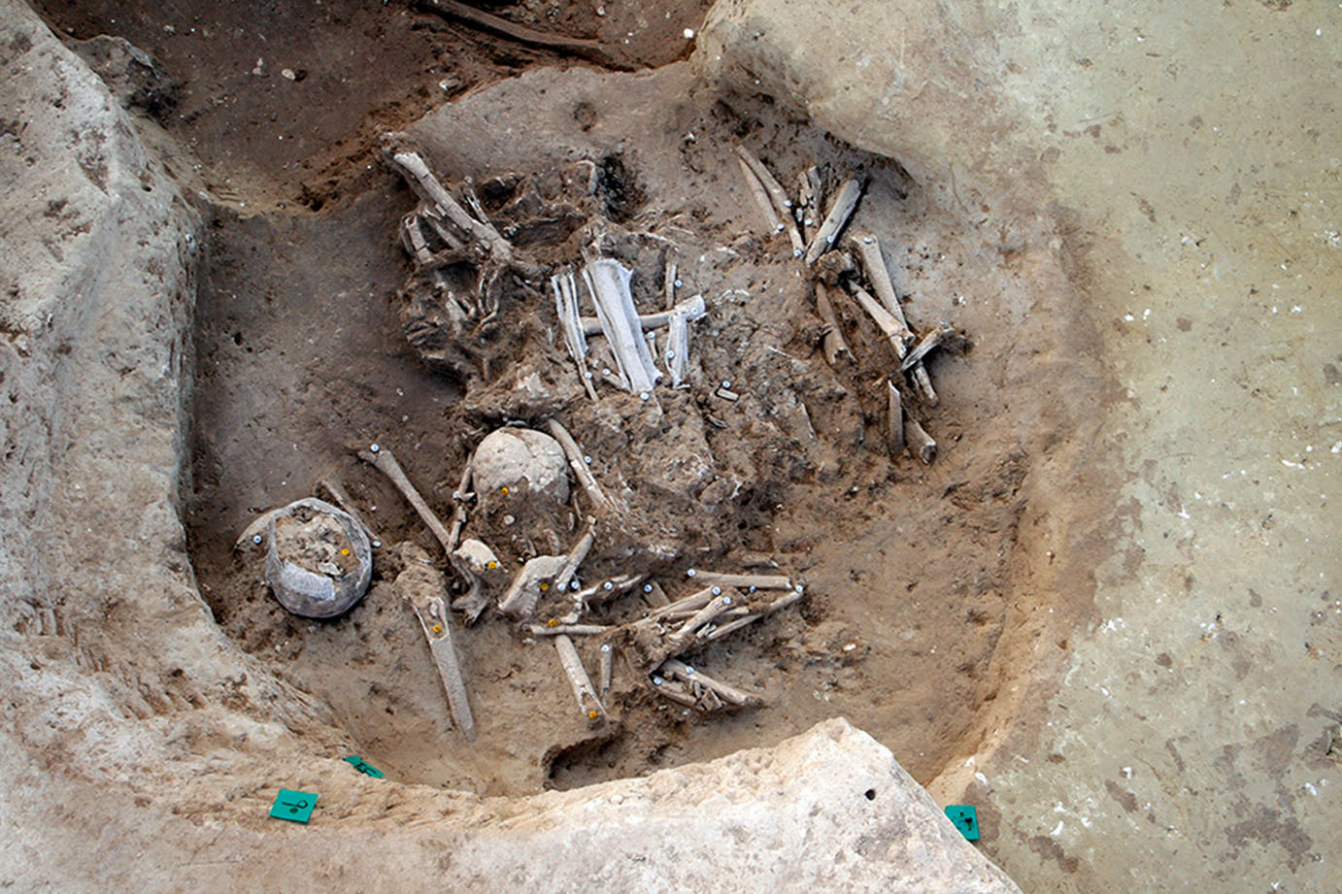
Human bone deposits of Structure 5 at Calle Dinamarca N^os^ 3–5. North in this photo is towards the bottom left-hand corner. Photo: Ana Pajuelo Pando

**Fig. 8:**
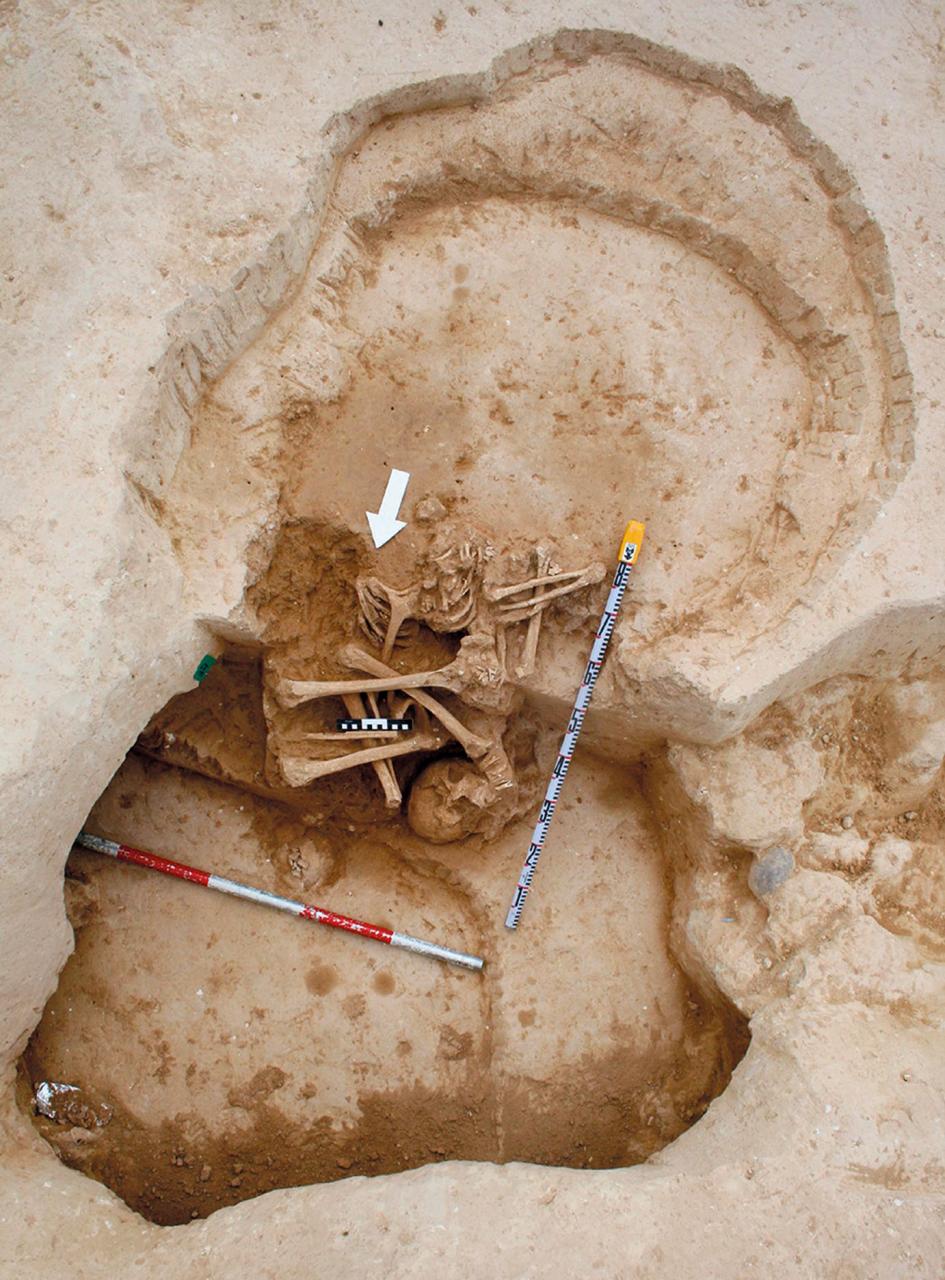
Structure 28 at Calle Dinamarca N^os^ 3–5. Photo: Ana Pajuelo Pando

Calle Dinamarca N^os^ 3–5 is located on the western side of the site. A rescue excavation in 2009 prior to new construction led to the discovery of five prehistoric structures, four of which (numbers 5, 28, 48 and 51) were excavated (Pajuelo Pando and López Aldana 2013a; Pajuelo Pando et al. 2013). Structures 48 and 51 were rather simple. Structure 5 appears to be an artificial cave, with a niche to one side (Figs. 6, 7), while Structure 28 is a more open and trilobate negative feature (Fig. 8). Excavations were conducted only to the level of destruction by the foundations of the new building, so the bottom of neither structure was reached (according to the criteria of administration officials, in some cases developers are only required to fund excavation down to the level to which new construction will cause destruction). Further skulls could be seen in a remaining deposit at the base of Structure 5, and further human remains were seen in a remaining deposit, labelled Context 32, one of the three parts of Structure 28. These two structures were close together, but were not connected underground.

These structures seem to have been predominantly used for successive primary inhumations, with bodies tightly flexed, especially in the lower limbs. According to field observations (no anthropological analysis of this collection is available at this time), an MNI of 63 people was recorded, although numerous other human remains were left *in situ* in unexcavated stratigraphic units. Little material culture was found. There was a slate arrowhead in Structure 51, and Structure 5 yielded a small pot, two worked *Patella* sp. shells, two bone-pin shafts, a rock-crystal arrowhead, 180 perforated beads of clinochlore (a phyllosilicate mineral of the chlorite group: we thank Carlos Odriozola Lloret for this information), a retouched blade, an adze fragment and a small ‘tolva’ idol made of ivory.

#### Results and Models

Samples from 14 individuals from Structures 5 and 28 were submitted for radiocarbon dating, and have produced a total of 21 radiocarbon ages for inclusion in the overall site chronological model. Eleven individuals have been dated from Structure 5 and a further three from Structure 28. The three individuals from Structure 28 are fully articulated and come from two layers, with Layer 32 (Individuals 4 and 13) underlying Layer 13 (Individual 1). The two measurements from Individual 4 and three measurements from Individual 1 are statistically consistent and the weighted mean for each burial has been included in the model (28.13 Ind. 1 and 28.32.4; Table 3).

The dated samples from Structure 5 come from Layer 60 in what has been called the Main Structure and Layer 31 in the SW Niche. From this Main Structure there are replicate results from two articulated skeletons from the base of Layer 60 (Individuals 1 and 2), along with a result (OxA-30335) from semi-articulated Individual 29. The replicate measurements on Individual 2 are statistically consistent at 3σ, and so their weighted mean has been included in the model (mean 5.60.2; Table 3); but those from Individual 3 are statistically inconsistent at more than 3σ and so each measurement has been included in the preliminary model separately (OxA-32307 and SUERC-47667; Table 3). Above these three skeletons, there is a sequence of a further two semi-articulated individuals (3: OxA-28239, overlain by 5: OxA-28240) and three results (SUERC-53945, SUERC-53947 and OxA-30338) from samples associated with the disturbed burials of Individuals 6, 7 and 14, respectively, which are not modelled with any stratigraphic relationship to Individuals 3 and 5, but are later than the three skeletons at the base of the layer.

Within the SW Niche the three burials come from a single layer. Individuals 101 and 105 were semi-articulated, while 25 was disarticulated but formed a discrete group of bones that was interpreted as a secondary deposit in the niche that originated in the Main Structure. The replicate results on Individual 25 are statistically consistent and so their weighted mean has been included in the analysis (5.31.25; Table 3), but those from Individual 101 are statistically inconsistent at more than 3σ and so have been included separately in the preliminary model (OxA-28241 and SUERC-47669; Table 3).

The initial model has poor agreement between the calibrated radiocarbon dates and the archaeological sequence just described (Amodel: 14; model not shown). The two divergent dates from 5.60.1 have individual indices of agreement of A: 1 (SUERC-47667) and A: 118 (OxA-32307); and those from 5.31.101 have values of A: 106 (SUERC-47669) and A: 8 (OxA-28241). OxA-32307 and SUERC-47669 clearly provide the most accurate estimates of the age of these individuals (SUERC-476667 and OxA-28241 being respectively anomalously young and anomalously old in relation to the other radiocarbon dates in the model).

**Fig. 9:**
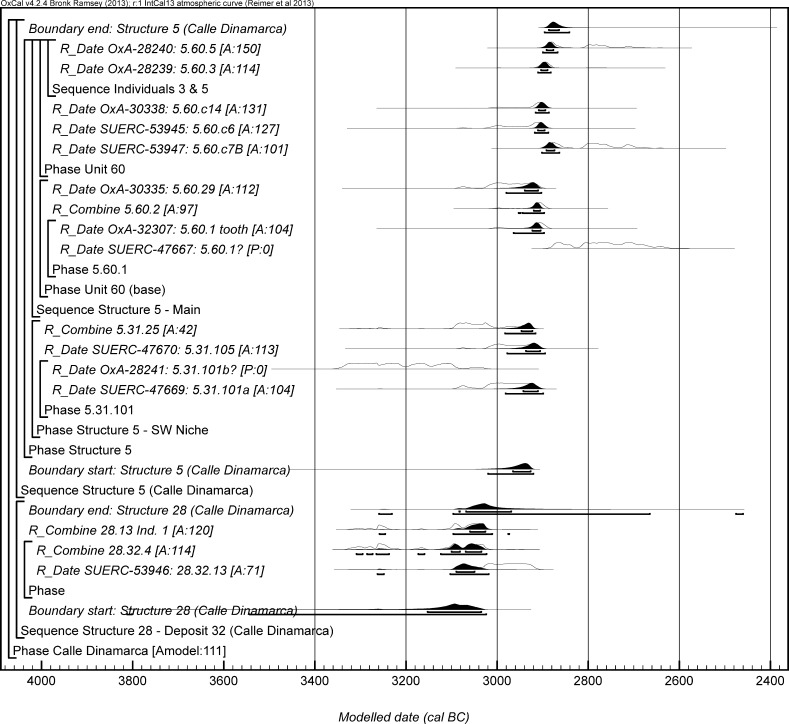
Chronological model for the burial activity at Calle Dinamarca N^os^ 3–5. The format is as described in Fig. 4. The large square ‘brackets’ down the left-hand side along with the OxCal keywords define the overall model exactly

**Fig. 10:**
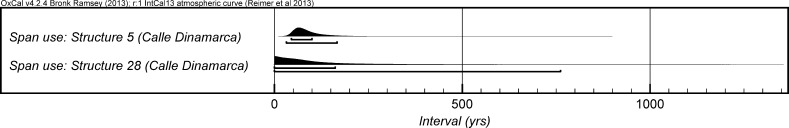
Probability distributions for the number of years over which burial activity took place at structures 5 and 28 of Calle Dinamarca N^os^ 3–5. The distributions are derived from the model defined in Fig. 9

The revised model, which omits SUERC-476667 and OxA-28241, has good overall agreement (Amodel: 111; Fig. 9). The model estimates that the dated burial activity in Structure 28 began by *3545*–*3020* *cal BC* (*95%* *probability*; *start: Structure 28 (Calle Dinamarca)*; Fig. 9), probably by *3155*–*3030* *cal BC* (*68%* *probability*). The model suggests that Structure 28 was in use for at least *1*–*765* *years* (*95%* *probability*; *use: Structure 28 (Calle Dinamarca)*; Fig. 10), probably for at least *1*–*165* *years* (*68%* *probability*). Burial activity in Structure 28 ended in either *3260*–*3230* *cal BC* (*1%* *probability*; *end: Structure 28 (Calle Dinamarca)*; Fig. 9) or *3100*–*2660* *cal BC* (*94%* *probability*), probably in *3070*–*2965* *cal BC* (*68%* *probability*).

The dated burial activity in Structure 5 began by *3020*–*2920* *cal BC* (*95%* *probability*; *start: Structure 5 (Calle Dinamarca)*; Fig. 9), probably by *2970*–*2925* *cal BC* (*68%* *probability*). Structure 5 was in use for at least *30*–*170* *years* (*95%* *probability*; *use: Structure 5 (Calle Dinamarca)*; Fig. 10), probably for at least *45*–*100* *years* (*68%* *probability*). The structure ceased to be used in *2900*–*2840* *cal BC* (*95%* *probability*; *end: Structure 5 (Calle Dinamarca)*; Fig. 9), probably in *2890*–*2860* *cal BC* (*68%* *probability*).

It must be reiterated that the excavation of these two features did not reach the bottom of the structures and so the modelled estimates for the beginning of burial activity should be considered as *termini ante quos* for the construction and use of both features. The two date estimates provide a marker for when activity had begun, but we cannot be certain for how long prior to these dates burial was taking place.

### Instituto de Enseñanza Secundaria (IES)

More or less at the centre of Valencina, the plot of land where a new secondary school was to be built was excavated in 2005–2006, leading to the discovery of over 150 negative features, mostly of prehistoric date (Vargas Jiménez et al. 2010).They have sub-circular, circular, oval or poly-lobulate plans and range from less than 1 m in diameter to big features like Structure 34, which covered 26.5 m^2^. These seem to have served a variety of purposes, including metallurgical production, occupation and burial (Vargas Jiménez et al. 2010; Nocete Calvo et al. 2013).

Structures 64 and 281/283 were used for burial. Structure 64 is a shallow cut, circular in plan, and 1 m across. Two articulated skeletons were identified in its upper part (Vargas Jiménez et al. 2010), but the bulk of this complex deposit was removed in blocks for excavation in the Valencina museum. In these, a minimum of eight further skulls have been identified, and ongoing investigation of the lifted blocks suggests the presence also of other, smaller, post-cranial bones. No formal anthropological study has yet been made, and no material culture was found.

**Fig. 11:**
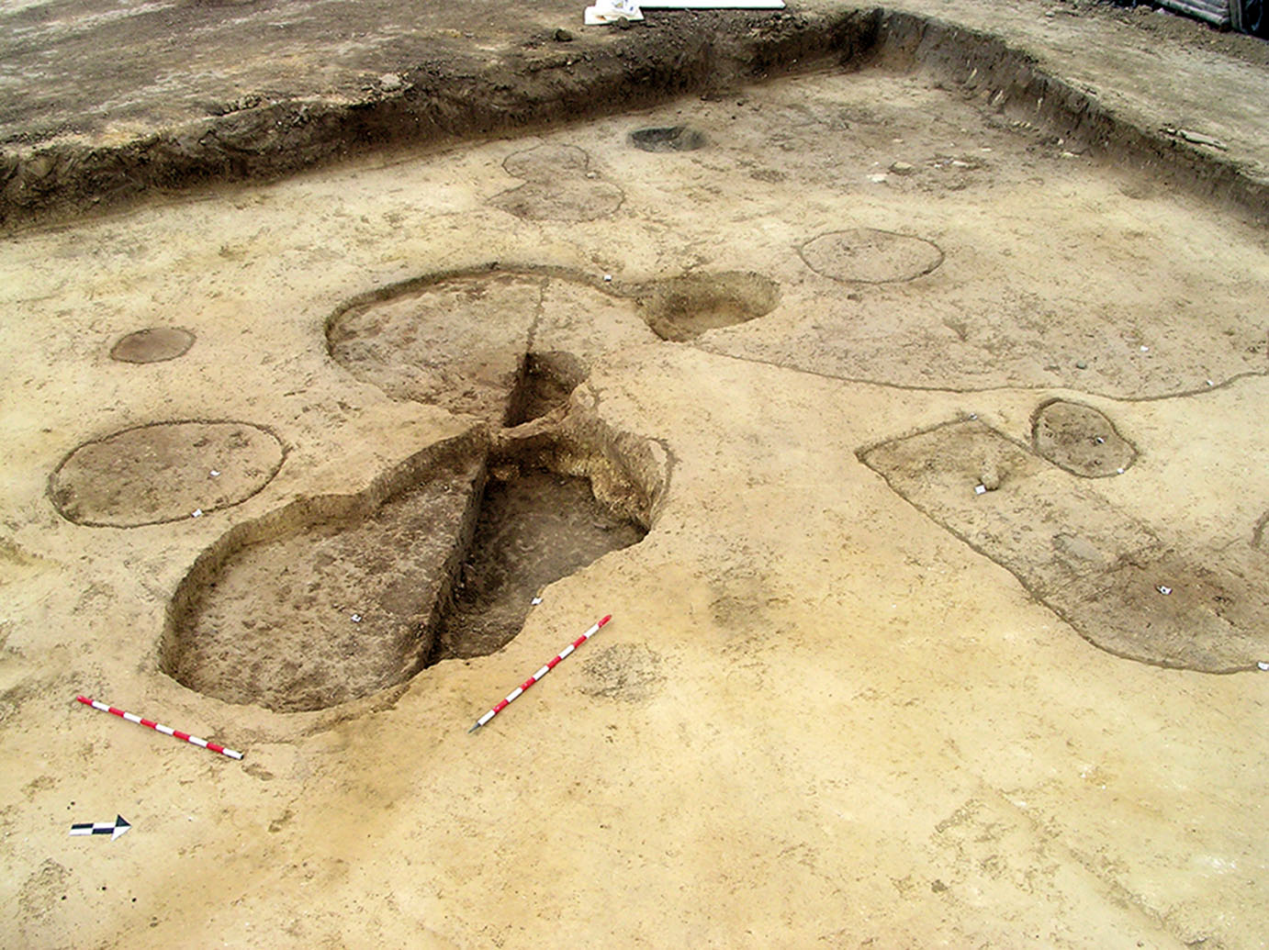
Excavation area of IES with Structure 402 in the foreground. Photo: Juan Manuel Vargas Jiménez

Structure 402/403 (Fig. 11) was interpreted as an ivory workshop, as chips and half-worked items of this material and a copper saw were found in it (Vargas Jiménez, Nocete Calvo and Schuhmacher 2012b). Radiocarbon dates have also been obtained from Structure 34, interpreted as a large hut floor by the excavators, and an area of domestic debris, 223.

#### Results and Models

The radiocarbon dating in the IES sector investigated the remains of Structure 402/403, the ivory workshop, and Structure 64, which included human remains. In addition to the 12 radiocarbon measurements made on samples submitted from the ToTL project, two further results are available from samples submitted by the excavators to the Tandem Laboratory, University of Uppsala (Vargas Jiménez, Nocete Calvo and Schuhmacher 2012b, p. 74).

All the lower remains in Structure 64 were compressed, with anatomical connections only apparent when studied by the physical anthropologist. Five of the ten identified inhumations in the blocks that were lifted and are being excavated under controlled laboratory conditions have been dated. The two results on 64.19.6 (OxA-28287 and SUERC-47676) are statistically consistent at 2σ, while those on 64.13.cranium (OxA-30381 and SUERC-53963) are inconsistent at 2σ, but consistent at 3σ (Table 3). These results were combined prior to calibration to form the weighted means 64.19.6 and 64.13.cranium. These two inhumations and the human remains that make up the bulk of Structure 64, including those dated (64.13.10, 64.19.cranium, 64.12.4, 64.16.cranium, and 64.12.cranium) were overlain by the partially articulated remains of Individual 14/728 in deposit 1 (sample 64.1).

Structure 64 was cut after Structure 34 had gone out of use, as the cut for its construction appears to have caused the collapse of part of the abandoned structure. The single radiocarbon date from Structure 34 (Ua-32886), however, is on oak charcoal from metalworking debris within its backfill. This sample, therefore, only provides a *terminus post quem* for the infilling of Structure 34 and has no relationship to Structure 64 (it was deposited after its construction, but as the sample may have an inbuilt age this relationship cannot be used in the model). This backfilling occurred in or after 2910–2835 cal BC (34% probability; Ua-32886; Stuiver and Reimer 1993) or 2815–2670 cal BC (61% probability), probably in or after 2895–2860 cal BC (26% probability) or 2810–2755 cal BC (35% probability) or 2720–2700 cal BC (7% probability).

**Fig. 12:**
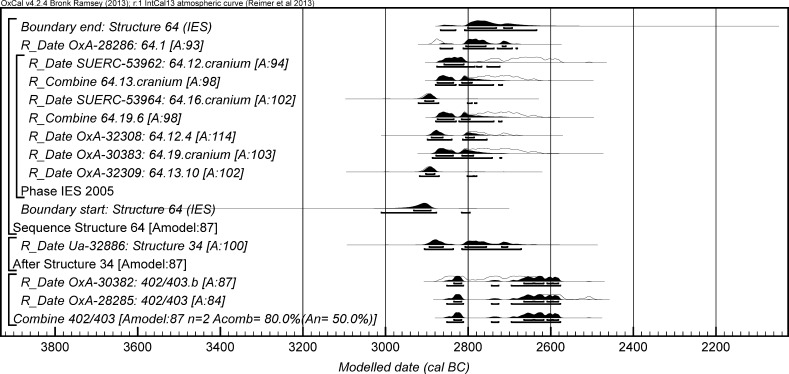
Chronological model for the burial activity associated with Structure 64 and for ivory working in Structure 402/403 in the IES sector, with the calibrated radiocarbon date from the infill of Structure 34. The format is as described in Fig. 4. The large square ‘brackets’ down the left-hand side along with the OxCal keywords define the overall model exactly

**Fig. 13:**
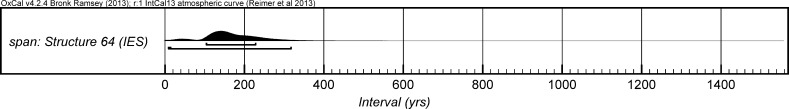
Probability distribution for the number of years over which burial activity associated with Structure 64 at IES took place. The distribution is derived from the model defined in Fig. 12

The model estimates that Structure 64 was built in either *3010*–*2875* *cal BC* (*94%* *probability*; *start: Structure 64 (IES)*; Fig. 12) or *2820*–*2795* *cal BC* (*1%* *probability*), probably in *2935*–*2890* *cal BC* (*68%* *probability*). The burial activity associated with this structure ended in either *2870*–*2830* *cal BC* (*6%* *probability*; *end: Structure 64 (IES)*; Fig. 12) or *2810*–*2630* *cal BC* (*89%* *probability*), probably in either *2805*–*2730* *cal BC* (*59%* *probability*) or *2715*–*2690* *cal BC* (*9%* *probability*). The structure was in use for *5*–*320* *years* (*95%* *probability*; *span: Structure 64 (IES)*; Fig. 13), probably for *100*–*230* *years* (*68%* *probability*).

It should be noted that the model for the chronology of Structure 64 presented in Fig. 12 is provisional, as the sequence of deposition of corpses within the tomb is unknown until excavation of the blocks in Valencina Museum has been completed. The radiocarbon measurements on the eight dated individuals are not statistically consistent (T′ = 26.6; T′ (5%) = 14.1; ν = 7), which suggests that the deposit formed over some time and was not a single event; when the relative sequence of the skeletons is available, this will provide further prior information that can be included in a revised model.

Two results are available on ivory chips from pit 402/403 (OxA-28285 and OxA-30382). The measurements are statistically consistent (T′ = 2.1; T′(5%) = 3.8; ν = 1), so the samples could be the same actual age. On the assumption that the chips derive from the same episode of ivory working, the results have been combined after calibration and provide a date estimate of *2855*–*2810* *cal BC* (*21%* *probability*) or *2745*–*2725* *cal BC* (*3%* *probability*) or *2695*–*2575* *cal BC* (*71%* *probability*; *402/403*; Fig. 12), probably *2835*–*2815* *cal BC* (*15%* *probability*) or *2665*–*2615* *cal BC* (*35%* *probability*) or *2610*–*2580* *cal BC* (*18%* *probability*), for the procurement of the ivory that was deposited in the pit.

A second date (Ua-32887; Table 3) on a fragment of oak charcoal from Domestic Area 223 is about a millennium later than the activity presented here, and so has also been excluded from the model.

### PP4-Montelirio Sector and Montelirio Tholos Tomb

**Fig. 14:**
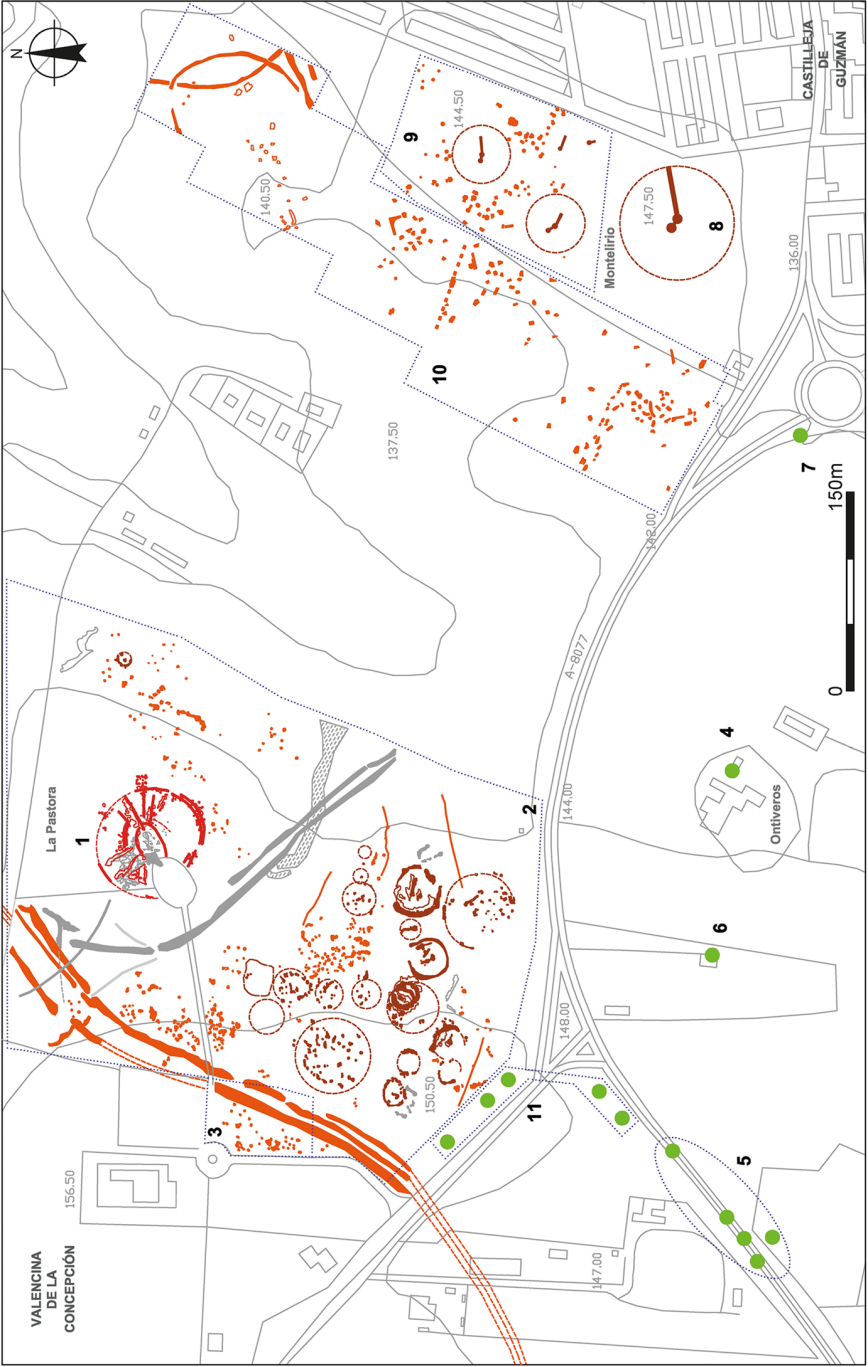
Main sectors and features in the southeastern quadrant of Valencina: *1* La Pastora; *2* La Pastora-El Cuervo area surveyed by magnetometry; *3* Parcela Municipal; *4* Ontiveros; *5* El Roquetito; *6* Structure 3 of Nuestra Señora de los Reyes; *7* Structure 2 of Nuestra Señora de los Reyes; *8* Montelirio tholos; *9* PP4-Montelirio; *10* Montelirio-La Pastora area surveyed by magnetometry; *11* Mataherrera-La Pastora sector. Design: Juan Manuel Vargas Jiménez

These two adjacent sectors are in the southeastern part of the site (Fig. 2). In keeping with the rest of the paper, monuments and structures in them are presented by sector, but it is best to think of the two together as a single area including megalithic and non-megalithic structures, with and without human remains. Megalithic Structure 10.042–10.049 is the largest structure found at the PP4-Montelirio sector, but far smaller than the Montelirio tholos, while a third, unexcavated structure remains roughly between them (Fig. 14).

The PP4-Montelirio sector was excavated between January 2007 and February 2008. A total of 134 Copper Age structures were identified, both megalithic and non-megalithic, of which 61 contained human remains. A summary of the results of this intervention is available in Mora Molina et al. (2013). In January 2011 the Research Group *ATLAS* (HUM-694) from the University of Seville began a project to study the collection of finds from this excavation currently kept in the Archaeological Museum of Seville (approximately 100 boxes). By January 2017, work carried out as part of this project included: a full inventory of finds and a preliminary assessment of the overall excavation results (Mora Molina 2011; Mora Molina et al. 2013); a full report on the sector’s faunal remains (Liesau et al. 2014); a bioarchaeological analysis of the human remains found in various structures (Robles Carrasco 2011; Robles Carrasco and Díaz-Zorita Bonilla 2013; Robles Carrasco et al. 2017); the study and restoration of several ivory items found in Structure 10.042–10.049 (García Sanjuán, Luciañez Triviño et al. 2013a; Luciañez Triviño et al. 2014); the geochemical characterisation of cinnabar pigments (Rogerio-Candelera et al. 2013) and of an exceptional piece of amber (Murillo-Barroso and García Sanjuán 2013) also from Structure 10.042–10.049; and the analysis of various rock-crystal objects (including the dagger blade from the upper level of Structure 10.049 (Morgado Rodríguez et al. 2016), as well as of a large gold plaque found in Structure 10.029, a simple pit with no human remains (Murillo-Barroso et al. 2015).

#### PP4-Montelirio Structure 10.042–10.049

**Fig. 15:**
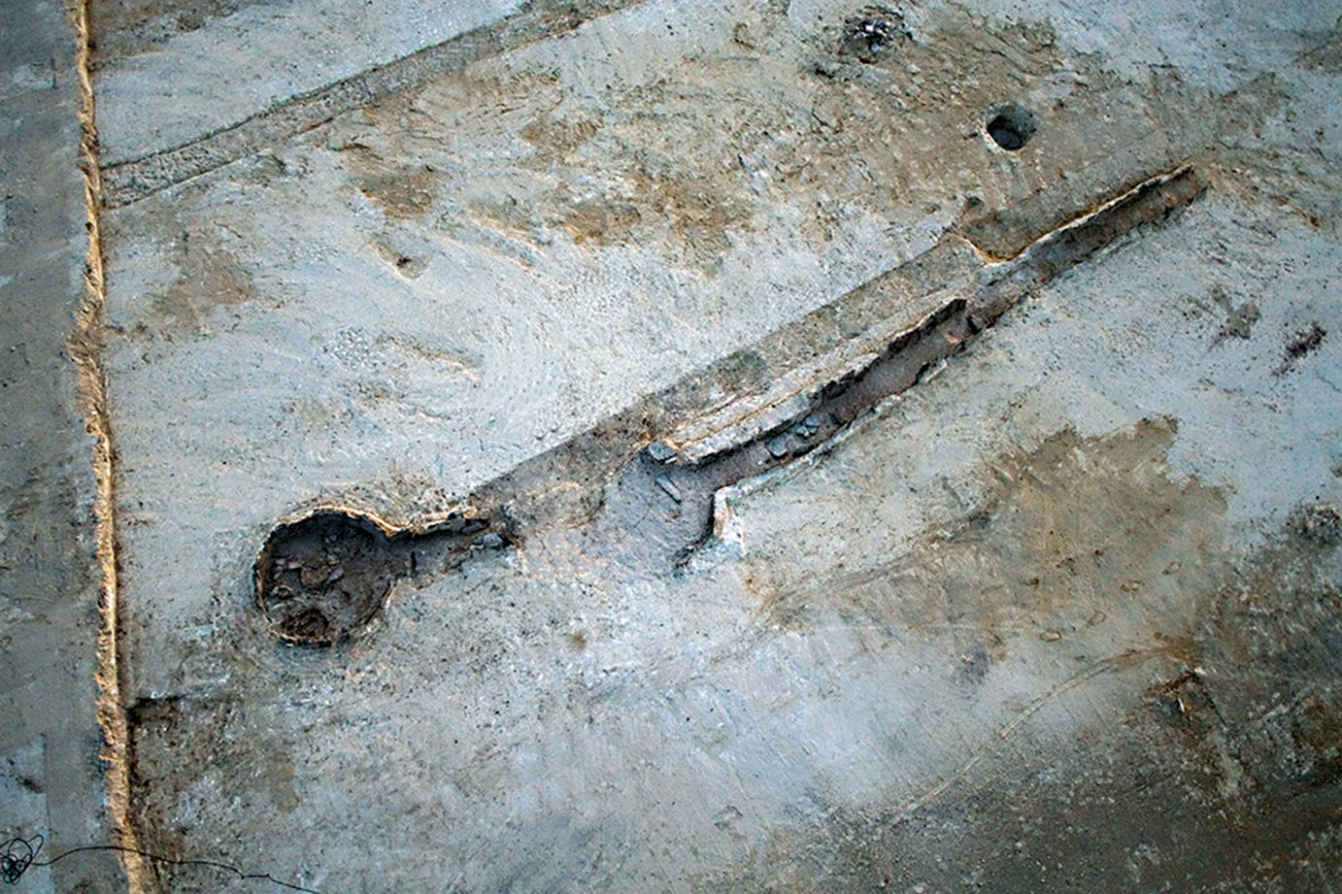
Overview of PP4-Montelirio Structure 10.042-10.049. Photo: José Peinado Cucarella

Structure 10.042–10.049 is the largest megalithic construction found at PP4-Montelirio, and one of the largest in the whole of Valencina (Fig. 15). A short description will be provided here; for a more extended one see García Sanjuán et al. (2018). This construction presents an outer access corridor 13 m long and 0.7 m wide, made from numerous slate slabs; at the end of this corridor is the first chamber, with a circular plan of 2.57 m in diameter, which was disturbed by later activity. In the space between the access corridor and the first chamber (Structure 10.042), the skeletal remains of four individuals were identified, as well as some grave goods, including more than two thousand beads covered in red pigment, fragments of a fired clay figurine, more than eight hundred sherds of pottery (some of them intrusive wheel-thrown ones), fragments of 12 arrowheads (nine of which are of the long-barbed type found in large numbers in Montelirio), three blade fragments, some lithic chipping debris and very fragmented ivory objects. Beyond the first chamber, a second corridor, 2.52 m long by 0.51 m wide and formed from several slate slabs, separates the first and the second burial chambers. This second chamber, found in a much better state of preservation than the first, has a maximum diameter of 2.1 m and is again formed by numerous slabs of slate. The excavation inside the second chamber led to the identification of two deposits stratigraphically separated by a set of horizontally laid slate slabs, which may be interpreted as some sort of ‘seal’ between the lower and upper depositional levels.

**Fig. 16:**
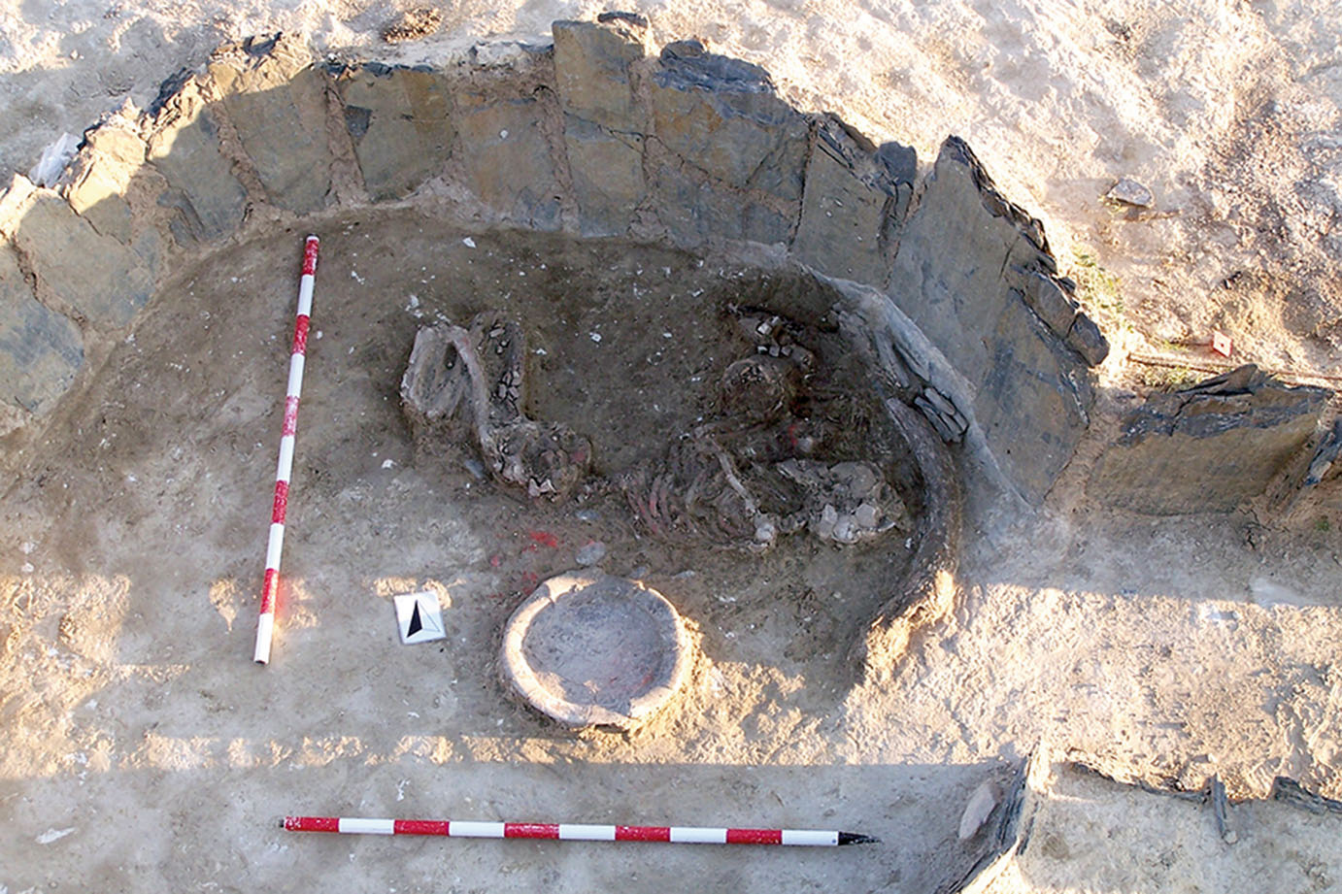
Lower level of PP4-Montelirio Structure 10.049 with individual inhumation of an adult male. Photo: José Peinado Cucarella

The lower depositional level of the second chamber (Structure 10.049) contained the articulated inhumation of a young male aged between 17 and 25 years (Robles Carrasco and Díaz-Zorita Bonilla 2013, p. 377) (Fig. 16). Lying in the foetal position on his right side, he was found in connection with a large set of grave goods, including an undecorated elephant tusk (laid above his head); an ‘almond-rim’ type plate; a set of 21 flint blades; a flint dagger blade in close spatial connection with an amber pommel (mentioned above); and various ivory objects, including two bowls and an assortment of non-diagnostic fragments (García Sanjuán, Luciañez Triviño et al. 2013a). Red pigment made from cinnabar had been sprayed all over this individual and the objects, surrounding him. In the upper depositional level of Structure 10.049, the finds comprised five complete or semi-complete ceramic plates—some with red pigment; 38 whole flint blades; 13 other lithic tools; a flint arrowhead with very long barbs (of the kind found in the Montelirio tholos); numerous ivory objects (most of them decorated and quite fragmented—among them a palette, a dagger hilt and the extreme terminal of a tusk, which were all decorated); 90 beads; an ostrich egg; and an outstanding rock-crystal dagger blade (Morgado Rodríguez et al. 2016).

#### PP4-Montelirio Structure 10.071

**Fig. 17:**
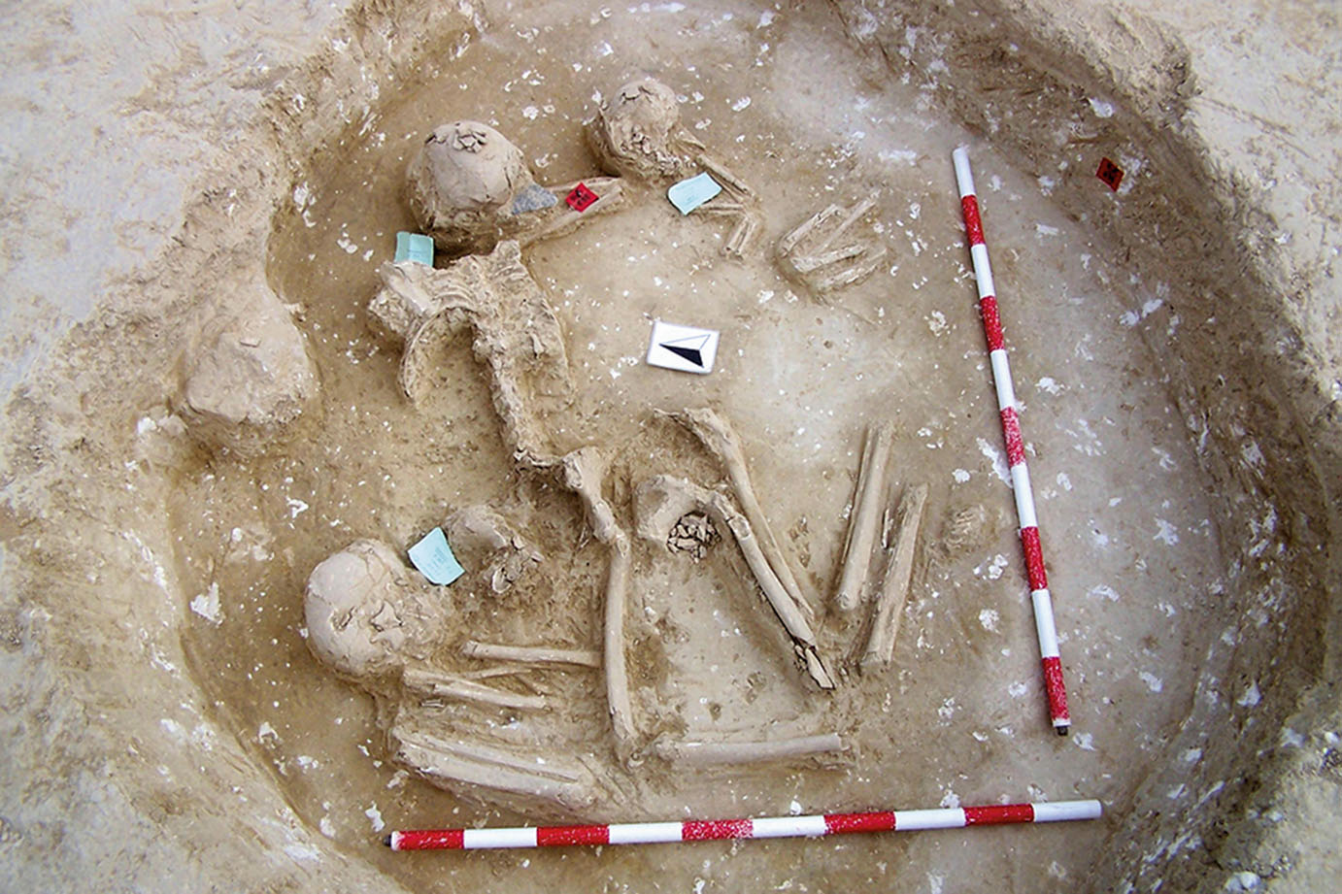
Lower level (Phase I) of PP4-Montelirio Structure 10.071 with three individual inhumations. Photo: José Peinado Cucarella

Structure 10.071 is a shallow, circular pit without stone elements, in which an MNI of seven was recorded (Mora Molina et al. 2013). A recent study (Cintas-Peña et al. 2018) has identified them as one female and six of indeterminate sex, of various ages, including infants (I and II), a young adult and a mature adult. Three individuals (1–3) in primary position and anatomical connection belong to an initial phase of use called Phase I (Fig. 17). Between this phase and the closure of the pit a further four individuals (4–7) were deposited. While some of the bones documented in this phase are articulated, most are not, probably as a consequence of the funerary re-use of this space. The final use of the structure is marked by the deposition of the latest individual (4).

#### PP4-Montelirio Structure 10.031

**Fig. 18:**
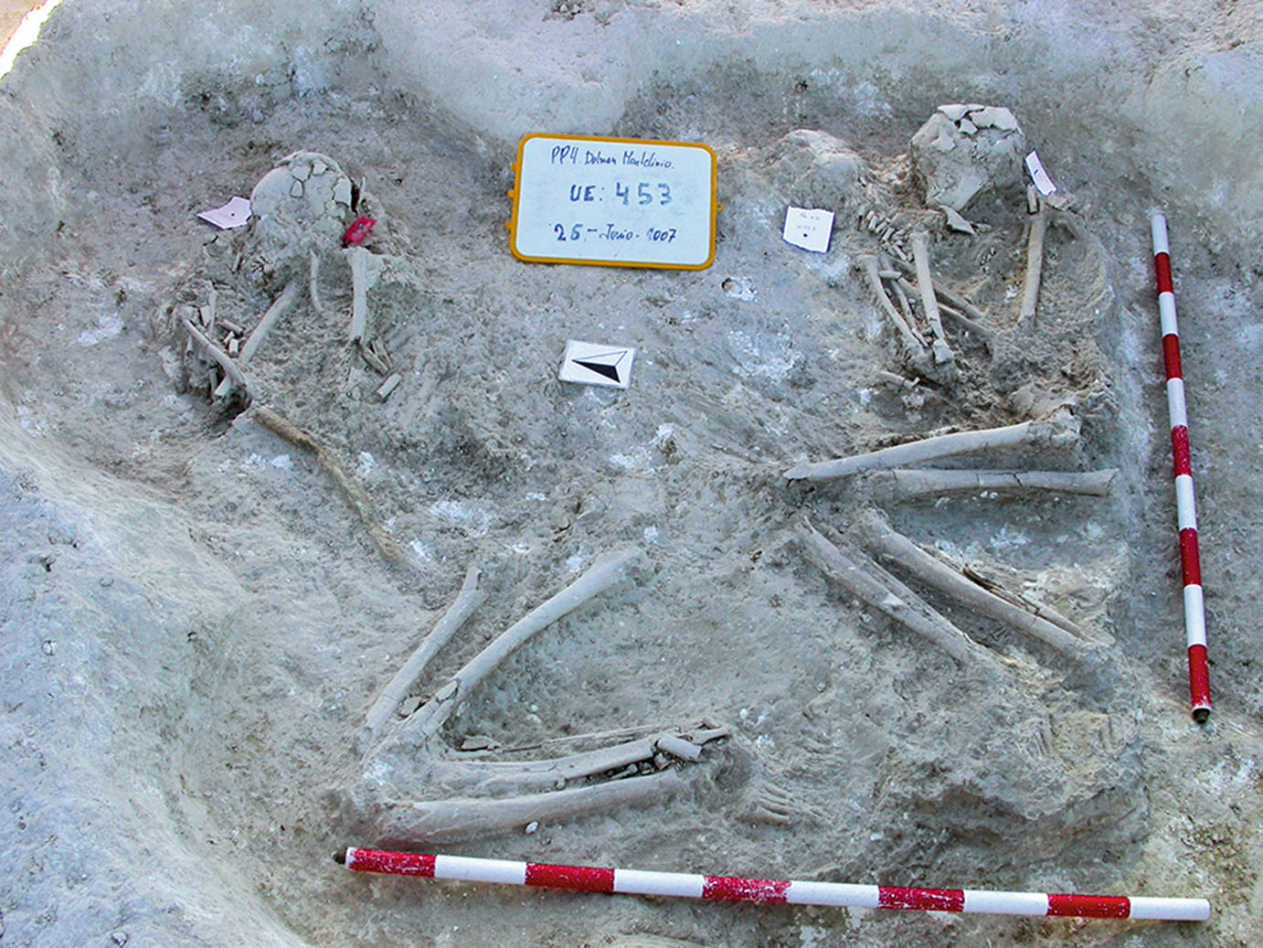
Human remains in PP4-Montelirio Structure 10.031. Photo: José Peinado Cucarella

Structure 10.031 is another shallow, oval negative feature (2.4 m long by 1.94 m wide) without stone elements (Mora Molina et al. 2013, p. 269) (Fig. 18). Within the structure, there was a primary multiple burial represented by a minimum of three individuals: two adults (male and female), 25–40 and 18–25 years old, respectively, and one subadult (6 ± 2–4 years old). The adults were articulated, on their right side with upper and lower limbs flexed. The report from the excavator does not provide enough contextual details to allow further interpretation of the deposition of the subadult individual. No grave goods were recorded, except for a single object made of bone or ivory (the surface of this object is badly eroded and to date it has been impossible to establish whether it is made of ivory or some other osseous material) that was associated with the male adult.

#### Results and Models

Four radiocarbon measurements from four individuals buried in the PP4-Montelirio sector were obtained by the University of Seville, two from Structure 10.042–10.049 and the other two from Structure 10.031 (Table 2). A series of results reported by the Erlangen laboratory on fragments of ivory from 10.049 are considered to be invalid due to low levels of collagen (García Sanjuán, Luciañez Triviño et al. 2013a, p. 625). The ToTL project attempted to date 17 samples of human bone and elephant ivory from this sector. Thirteen of the samples, including all seven submitted from 10.049, produced little or no collagen and failed the Oxford and SUERC quality-control procedures. The four samples successfully dated by ToTL were teeth from three individuals in Structure 10.071. Collagen preservation in tooth dentine can be better than that in bone samples, since it can sometimes be protected from hostile burial environments by the tooth enamel.

Each of the three dated structures has between two and four measurements on different individuals. There are no stratigraphic relationships noted between skeletons buried in Structures 10.031 or 10.042, but a series of burials were dated from Structure 10.071. Individuals 1 and 2 were from the bottom layer, although Individual 2 partially overlay Individual 1. Individual 6 was from the middle of the structure, but was a disarticulated skull and so could have been displaced from an earlier burial. All three of these individuals, however, were earlier than Individual 4 who was buried in the upper part of the structure.

**Fig. 19:**
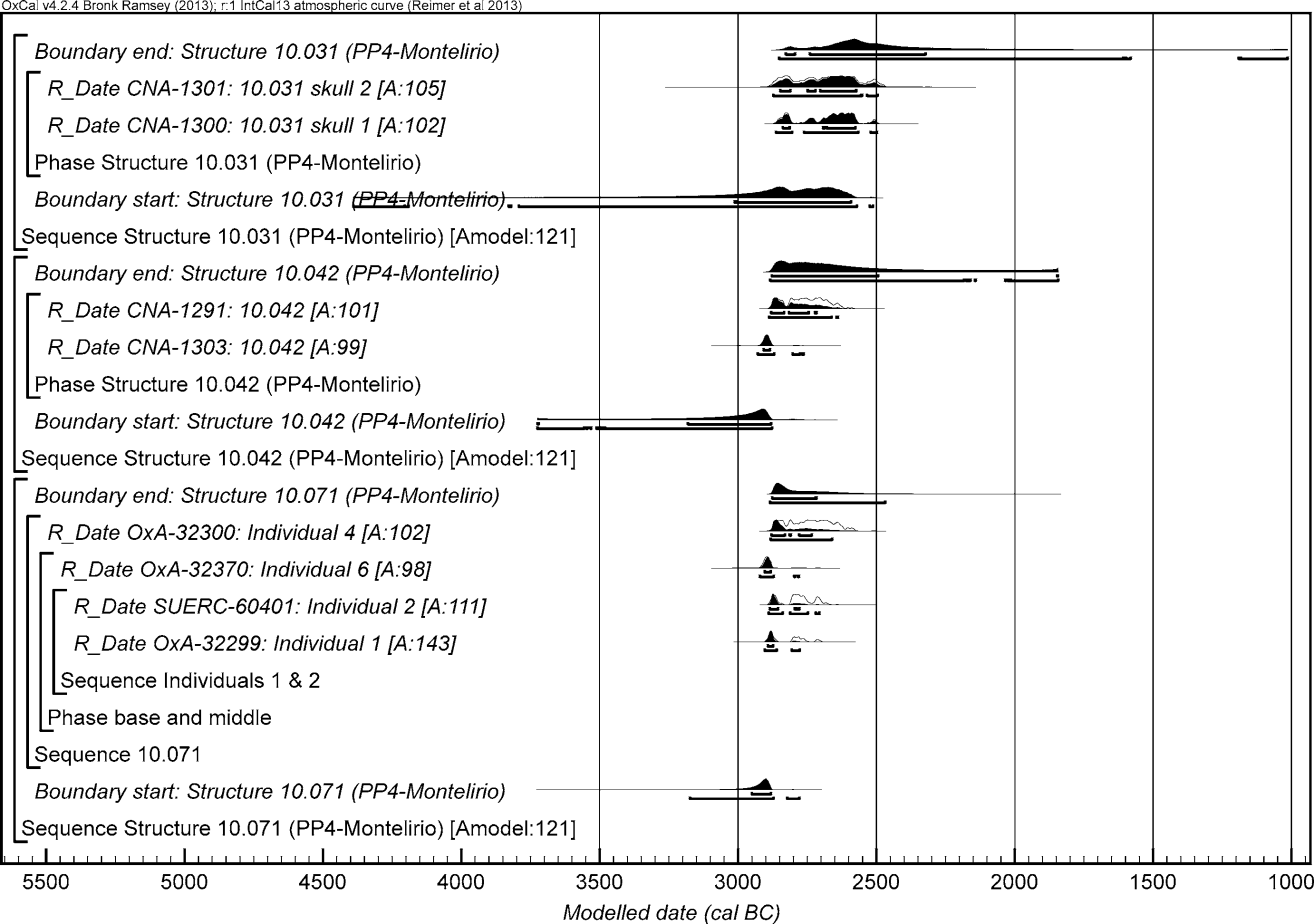
Chronological model for the burial activity associated with the three structures in the PP4-Montelirio sector. The format is as described in Fig. 4. The large square ‘brackets’ down the left-hand side along with the OxCal keywords define the overall model exactly

The model incorporates this stratigraphic sequence for Structure 10.071, but treats the use of each structure as an independent phase of activity. It has good overall agreement (Amodel = 121; Fig. 19), but the date estimates are comparatively imprecise because of the low number of results available. While consideration was given to modelling all the dates from this sector together in one related phase of activity, the fact that they derive from a varied range of structural types led us to maintain them as independent models, and accept the reduced precision.

The model for Structure 10.031 is based on two radiocarbon dates (CNA-1300 and CNA-1301) from the skulls of two individuals. The model estimates that the activity associated with their burial began in *4390*–*4190* *cal BC* (*3%* *probability*; *start: Structure 10.031 (PP4*-*Montelirio)*; Fig. 19) or *3795*–*2570* *cal BC* (*92%* *probability*), and probably in *3015*–*2590* *cal BC* (*68%* *probability*). The activity ended in *2855*–*1580* *cal BC* (*92%* *probability*; *end: Structure 10.031 (PP4*-*Montelirio)*; Fig. 19) or *1195*–*1010* *cal BC* (*3%* *probability*), and probably in either *2830*–*2790* *cal BC* (*3%* *probability*) or *2745*–*2320* *cal BC* (*65%* *probability*).

Although samples were submitted from both Structures 10.042 and 10.049, those from 10.049 all failed to produce results. Therefore, the model is for Structure 10.042, and includes two dates (CNA-1291 and CNA-1303) on the left ulna of two of the four recovered individuals. On this basis, burials in this structure began either in *3725*–*3555* *cal BC* (*9%* *probability*; *start: Structure 10.042 (PP4*-*Montelirio)*; Fig. 19) or *3515*–*2875* *cal BC* (*86%* *probability*), and probably in *3185*–*2880* *cal BC* (*68%* *probability*). The dated activity ended either in *2885*–*2155* *cal BC* (*86%* *probability*; *end: Structure 10.042 (PP4*-*Montelirio)*; Fig. 19) or *2045*–*1840* *cal BC* (*9%* *probability*), and probably in *2880*–*2490* *cal BC* (*68%* *probability*).

The model for Structure 10.071 is based on four radiocarbon results from four inter-stratified individuals. The model estimates that burial in this structure began in *3175*–*2870* *cal BC* (*93%* *probability*; *start: Structure 10.071 (PP4*-*Montelirio)*; Fig. 19) or *2825*–*2775* *cal BC* (*2%* *probability*), probably in *2950*–*2880* *cal BC* (*68%* *probability*). The burial ended here in *2885*–*2465* *cal BC* (*95%* *probability*; *end: Structure 10.071 (PP4*-*Montelirio)*; Fig. 19), and probably in *2880*–*2715* *cal BC* (*68%* *probability*).

#### The Montelirio Tholos

**Fig. 20:**
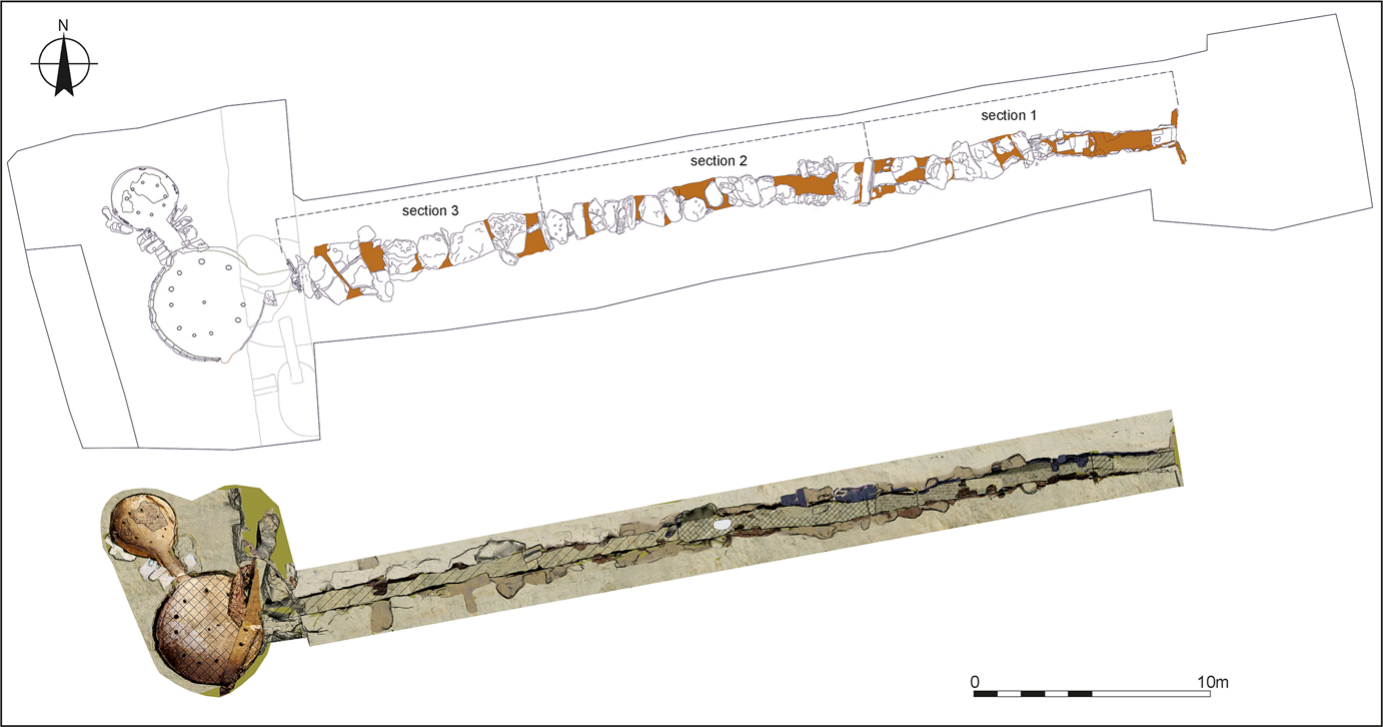
Plan of the Montelirio tholos. Above: linear drawing showing the corridor capstones; Below: ortho-photomosaic combined with laser scan after the excavation of the corridor and chambers. Design: Álvaro Fernández Flores

The Montelirio tholos was excavated in 2007 and later in 2009–2010. Given that an extended description is provided in Fernández Flores et al. (2016), only a short summary will be provided here. Montelirio is a major megalithic tomb, covered by a mound 75 m in diameter and 2.75 m high (Fig. 20). It has a long corridor (39 m) that leads into a large, circular, main chamber (4.75 m in diameter), which is connected to a smaller, secondary chamber through a small corridor. The corridors and the two chambers were excavated into a hillside, and large slate slabs lining their sides were painted in very bright red with cinnabar pigment (Hunt Ortiz et al. 2011; Bueno Ramírez et al. 2016). The corridors were roofed with large stone slabs and the chambers with vaults made of clay and marls.

There was an exceptional collection of artefacts. In the centre of the large chamber there were a stela and a varied series of objects, inhumations and ceramic vessels with food offerings were placed around them. Even though the outer parts of the large chamber had been disturbed, excavation documented 20 individuals. The processes of autolysis and skeletonisation of many of these individuals had not been completed when new inhumations took place. Radiocarbon dating suggests that the deposition of these inhumations may have taken place simultaneously or within a very short period (Bayliss et al. 2016). The 20 individuals identified are adults (11 cases between 20 and 35 years old), and at least 12 of them are female. While no clear sexual determination could be provided for the rest of the inhumations, five have been classified as ‘probably female’ and three as indeterminate. The small chamber was also badly disturbed, with nearly all human remains and objects recovered from disturbed deposits. There were at least two mature or adult individuals, one of them gracile and the other more robust. Material includes ivory tools, ivory zoomorphic figures (Luciañez Triviño and García Sanjuán 2016), fragments of golden sheets, and arrowheads. In the corridor, there were one primary and two secondary inhumations, as well as two altars, lithic blades and arrowheads.

Alongside La Pastora, Matarrubilla and perhaps Ontiveros (which has never been fully excavated) from Valencina itself and El Romeral from Antequera (Málaga), the Montelirio tholos stands out as one of the largest megalithic monuments ever built in Copper Age Iberia, and certainly with one of the most sumptuous sets of grave goods yet recorded.

#### Results and Models

Of the 22 radiocarbon results available for this monument, ten were published previously (Fernández Flores and Aycart Luengo 2013, p. 252) and 12 were obtained by the ToTL project. A full analysis of these 22 results has already been published in Spanish (Bayliss et al. 2016) as part of a large monograph presenting the study of this tomb (Fernández Flores et al. 2016).

Beneath the human skeletons in the main chamber was a layer of ash and charred material (Unit 166), from which two samples of unidentified charred material have been dated (CNA-589 and Ua-40805). These measurements are not statistically consistent (T′ = 20.8; T′ (5%) = 3.8; ν = 1), but provide a *terminus post quem* for the formation of the deposit and the deposition of the human remains above. Within this deposit there are ten results from five articulated burials in a sequence. The sequence begins with Individual 113, from which there are two results on bone (CNA-587 and Ua-40804) that are statistically consistent with one another (T′ = 1.3; T′ (5%) = 3.8; ν = 1), and a further two measurements on teeth (CNA-588 and OxA-32303) that are also statistically consistent with each other (T′ = 3.0; T′ (5%) = 3.8; ν = 1). However, the four measurements are not statistically consistent as a group (T′ = 48.0; T′ (5%) = 7.8; ν = 3). The two measurements from the radius of the individual are later than those from the teeth. Given the stratigraphic relationship between this individual and the overlying bodies it would appear that the two results on the bone are too recent, and they are thus excluded from the modelling. The two results from the teeth have been combined by taking a weighted mean prior to calibration to form mean 113: tooth (4116 ± 24 BP).

Individual 113 is overlain by Individuals 105 and 116, which are not stratigraphically related to one another. These two individuals are both overlain by Individual 103, from which there are three results on two teeth and a bone sample. The measurements are not statistically consistent at 2σ (T′ = 6.5; T′ (5%) = 6.0; ν = 2), but are at 3σ and have been combined prior to calibration to form mean 103 (4220 ± 17 BP). Individual 110 lies above 103. Elsewhere in the main chamber, Individuals 102 and 343 were also dated. These samples are later than the deposit of ash and charred material (Unit 166), but unrelated to the sequence of five superimposed corpses. There are three measurements from Individual 102, two on bone (CNA-585 and Ua-40803) and one on a tooth (CNA-586), that are statistically consistent (Table 3) and have been combined prior to calibration to form mean 102 (4191 ± 21 BP).

From within the tholos but outside the main chamber there are six radiocarbon dates. These have no direct stratigraphic relationships with the deposits in the main chamber. First, two further measurements come from bone fragments from the small chamber of the tholos that were recovered from Units 80 and 88 (Ua-40801–2). These dates are probably related to the general use of the structure, but cannot be confidently placed within the sequence of activity dated in the main chamber.

Two further burials in the corridor (Individuals 229 and 232) have been dated (OxA-30439 and OxA-30385), along with charcoal roundwood (OxA-32200) from a burnt deposit. Although the burials are similar in age to other activity in the tomb, the charcoal is approximately 2000 years younger, which may relate to a much later reuse of the corridor in the Early Iron Age, and since it is not related directly to the Copper Age activity, the result is excluded from further modelling. (Iron Age activity is not uncommon in Valencina’s Copper Age features, burial or otherwise: see García Sanjuán 2013, pp. 29–30, for a brief discussion.)

There is also a result (OxA-X-2535-32) from Individual 273, who was placed in a pit that was cut into the mound, outside the corridor, and partially covered by a capstone from the corridor, which suggests it pre-dates the construction of the tholos. The sample was reported with an OxA-X- number, reflecting the very low collagen yield from the bone (less than 0.5% weight collagen). Such a low yield makes it very difficult to be sure that all the material extracted was collagen, although the C:N ratio of this sample (3.3) is within the expected range for adequately preserved protein. The radiocarbon date is nearly two millennia earlier than any other date associated with the tholos. Either the result is anomalously early, or it is accurate and does not relate to the period of activity under consideration. In either case, it has been excluded from the modelling. If OxA-X-2535-32 accurately reflects the age of the crouched burial, this occurred in 4730–4545 cal BC (95% probability; Stuiver and Reimer 1993).

The model just described has poor overall agreement between the radiocarbon dates and the archaeological sequence (Amodel = 45; model not shown). Two dates have poor individual agreement in this model: the weighted mean of three results from Individual *103* (A: 26) and one of the dates on human bone from Unit 88 in the small chamber (*Ua*-*40802*; A: 22). Given the difference in the results from bone and teeth from Individual 113 in the main chamber, it is possible that either OxA-28245 or Ua-40802 or both are anomalous for technical reasons. Since the two measurements on teeth from Individual 103 are statistically consistent at 2σ (OxA-32304 and SUERC-47682, T′ = 0.4, T′ (5%) = 3.8, ν = 1), it seems likely that OxA-28245 is slightly too old. It has therefore been excluded from the analysis and the model re-run. This model has good overall agreement (Amodel: 70; model not shown), but *Ua*-*40802* still has poor individual agreement (A: 33). It is possible that this sample indeed dates a slightly later burial but, given the demonstrable difficulties of dating human bone at this site evident where we have replicate measurements on bone and teeth from the same individual (cf. Individual 113, CNA-587 and Ua-40804, Individual 103, OxA-28245), it is certainly possible that this result is slightly too recent.

**Fig. 21:**
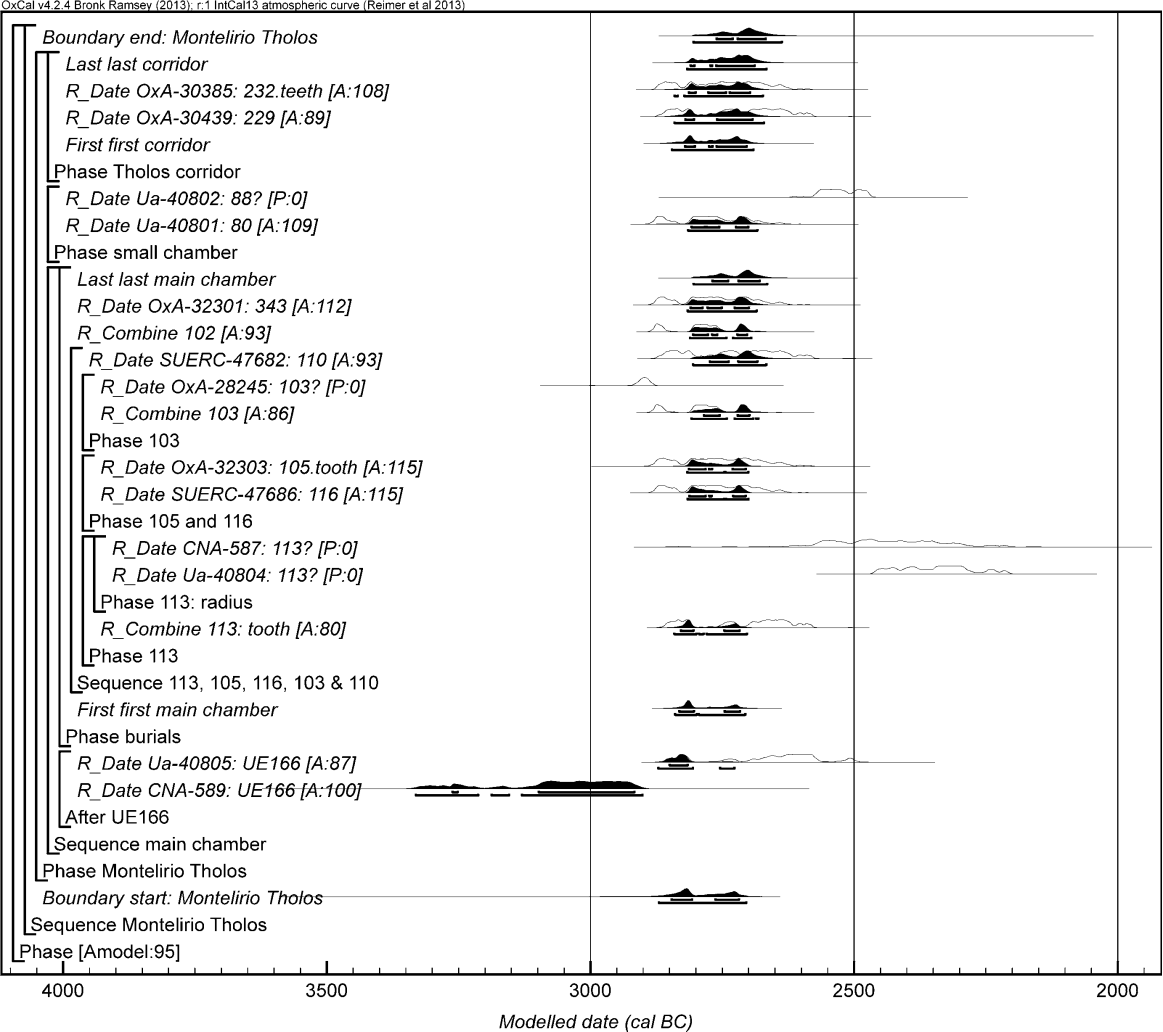
Chronological model for the burial activity associated with the Montelirio tholos (Model 1). The format is as described in Fig. 4. The large square ‘brackets’ down the left-hand side along with the OxCal keywords define the overall model exactly

**Fig. 22:**
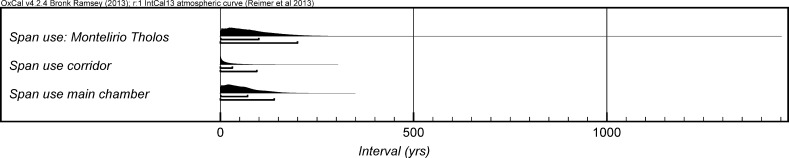
Probability distribution for the number of years over which burial activity inside the Montelirio tholos took place. The distributions are derived from the model defined in Fig. 21 (Model 1)

For this reason a further model has been constructed which excludes both OxA-28245 and Ua-40802 as anomalous for scientific reasons. This model (Model 1) has good overall agreement (Amodel: 95; Fig. 21), and all the individual dates have good individual agreement. This model suggests that the Montelirio tholos was constructed in *2875*–*2700* *cal BC* (*95%* *probability*; *start: Montelirio Tholos*; Fig. 21), probably in *2850*–*2805* *cal BC* (*38%* *probability*) or *2765*–*2715* *cal BC* (*30%* *probability*), and continued to be used for burial until *2805*–*2635* *cal BC* (*95%* *probability*; *end: Montelirio Tholos*; Fig. 21), probably in *2765*–*2730* *cal BC* (*18%* *probability*) or *2725*–*2665* *cal BC* (*50%* *probability*). Overall, it was in use for a period of *1*–*200* *years* (*95%* *probability*; *use: Monteliro Tholos*; Fig. 22), probably for *1*–*100* *years* (*68%* *probability*).

By calculating the first and last dated events in the main chamber and the corridor (we only have one dated sample from the small chamber now that we have excluded Ua-40802 as scientifically anomalous), we can assess the period at which different areas of the tomb were used for burial. Burial began in the main chamber in *2840*–*2705* *cal BC* (*95%* *probability*; *first main chamber*; Fig. 21), probably in *2835*–*2800* *cal BC* (*42%* *probability*) or *2750*–*2715* *cal BC* (*26%* *probability*). It continued until *2805*–*2660* *cal BC* (*95%* *probability*; *last main chamber*; Fig. 21), probably until *2770*–*2735* *cal BC* (*22%* *probability*) or *2720*–*2675* *cal BC* (*46%* *probability*), over a period of *1*–*140* *years* (*95%* *probability*; *use main chamber*; Fig. 22), probably a period of *1*–*75* *years* (*68%* *probability*). Burial in the corridor began in *2850*–*2690* *cal BC* (*95%* *probability*; *first corridor*; Fig. 21), probably in *2825*–*2800* *cal BC* (*19%* *probability*) or *2780*–*2700* *cal BC* (*49%* *probability*). It ended in *2820*–*2665* *cal BC* (*95%* *probability*; *last corridor*; Fig. 21), probably in *2810*–*2800* *cal BC* (*4%* *probability*) or *2775*–*2685* *cal BC* (*64%* *probability*), after a period of *1*–*95* *years* (*95%* *probability*; *use corridor*; Fig. 22), probably after a period of *1*–*35* *years* (*68%* *probability*).

The similarity of all these date estimates from Model 1 and the apparent brevity of activity in the tomb suggested by the formal estimates of its duration (Fig. 22) lead us to consider the suggestion that the processes of autolysis and skeletonisation of many of the individuals in the main chamber had not been completed when new inhumations took place, and so deposition may have taken place simultaneously or within a very short period (Pecero Espín 2016). In fact, when OxA-28245 is omitted, the radiocarbon determinations on all seven dated individuals from the main chamber are statistically consistent (T′ = 9.5; T′ (5%) = 12.6; ν = 6) and so, statistically, they could have all died at exactly the same time. Indeed, when this measurement, Ua-40802, and OxA-X-2735-32 (which is clearly inaccurate or related to a pre-tholos episode of activity on the site) are excluded, all the radiocarbon results from all the individuals from the tomb are statistically consistent (T′ = 11.7; T′ (5%) = 16.9; ν = 9). This would be compatible with the suggestion that burial in the entire tholos was a single episode.

**Fig. 23:**
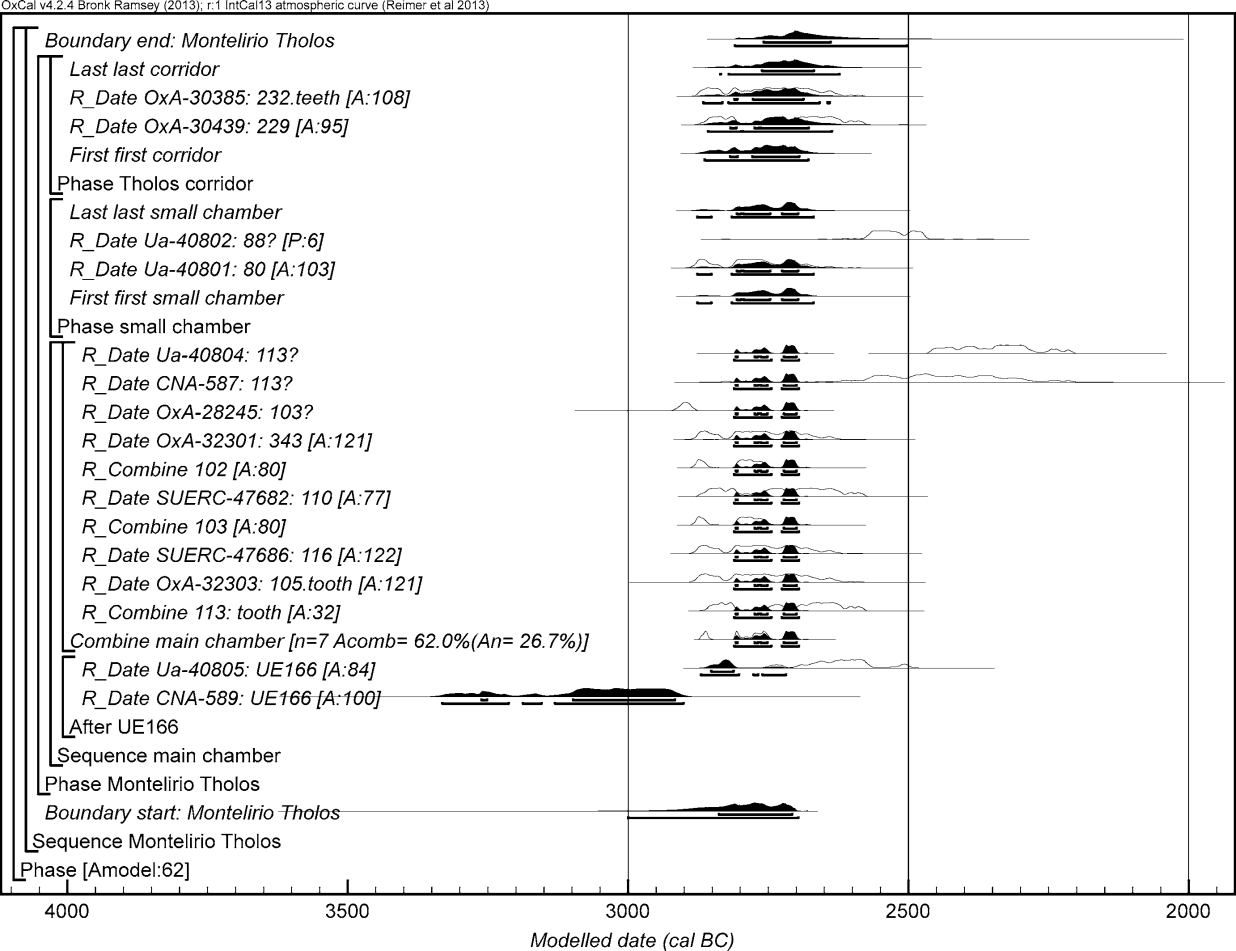
Alternative chronological model for the burial activity associated with the Montelirio tholos (Model 2). The format is as described in Fig. 4. The large square ‘brackets’ down the left-hand side along with the OxCal keywords define the overall model exactly

To explore this possibility two further models have been constructed. Model 2, in which only the burials in the main chamber are interpreted as a single event, has good overall agreement (Amodel: 62; Fig. 23), although *113: tooth* has poor individual agreement (A: 32). This model suggests that the 20 corpses were placed in the main chamber in *2815*–*2740* *cal BC* (*48%* *probability; main chamber*; Fig. 23) or *2730*–*2695* *cal BC* (*47%* *probability*), probably in *2810*–*2800* *cal BC* (*3%* *probability*) or *2775*–*2750* *cal BC* (*22%* *probability*) or *2725*–*2695* *cal BC* (*43%* *probability*). Model 3, in which all the burials from the tholos are assumed to have happened as one event, has poor overall agreement (Amodel: 59; model not shown), again with *113: tooth* having poor individual agreement (A: 35).

Models 1 and 2 are both statistically plausible and agree in placing the construction and use of the Montelirio tholos within a restricted period in the later 29th or 28th century cal BC. The radiocarbon dates are compatible with the interpretation of burial in the main chamber as a single event, but do not prove that this was the case. The radiocarbon evidence is also compatible with these burials occurring over a period of a few decades. The choice between these readings must thus be made on the basis of other evidence.

### Calle Mariana de Pineda s/n

**Fig. 24:**
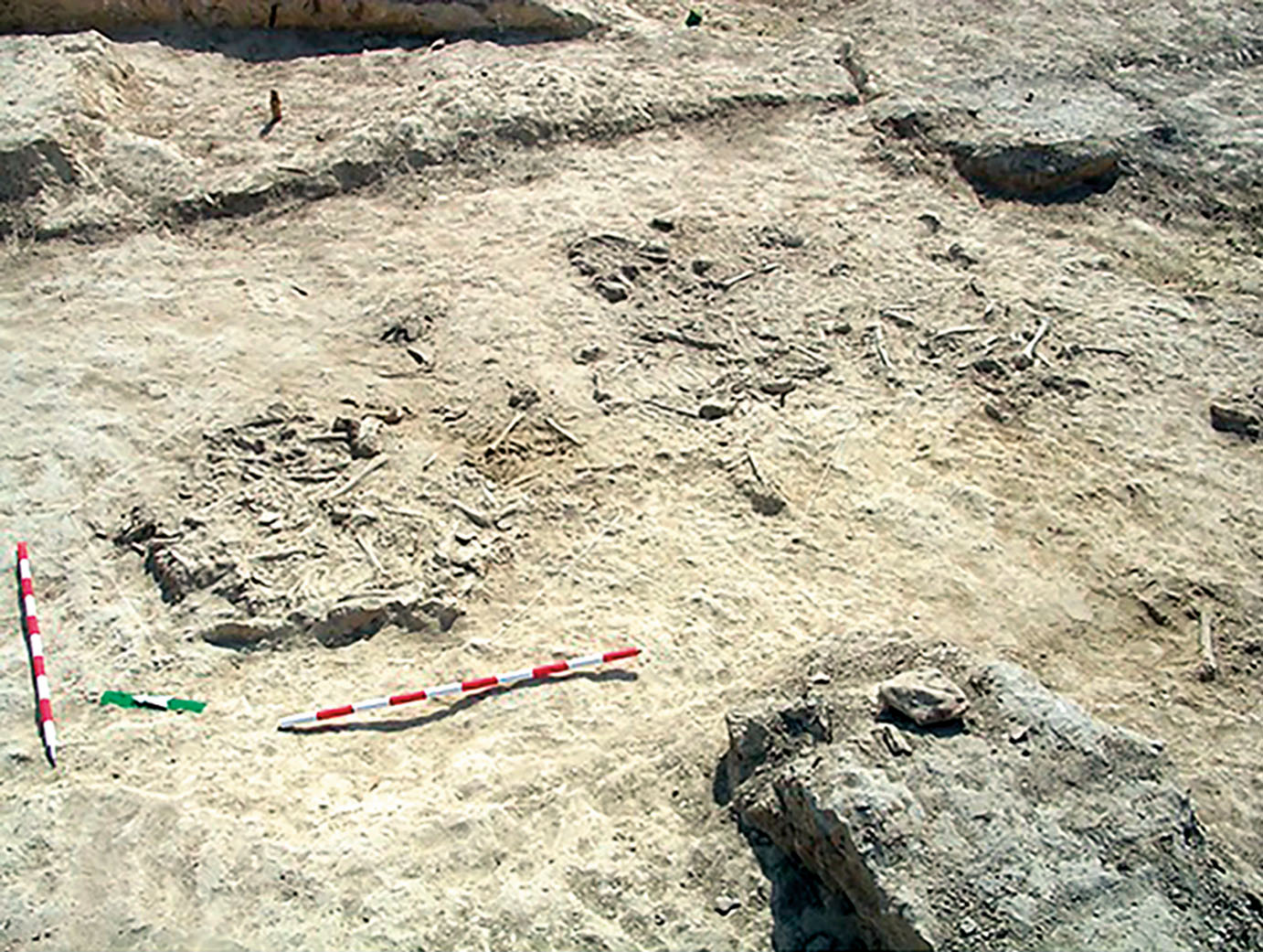
Human remains in the top of Structure 30 at Calle Mariana de Pineda s/n. Photo: Ana Pajuelo Pando

The Calle Mariana de Pineda s/n sector is located on the northwestern boundary of Valencina, on the western side of what some authors have labelled the ‘residential’ or ‘domestic’ area of the site (Fig. 2). Excavation in 2006 in advance of building works, comprising 4700 m^2^, revealed 56 prehistoric features, some of which were not fully excavated since they were below the level affected by the new constructions (Moro Berraquero et al. 2009; Pajuelo Pando and López Aldana 2013b). These features were of varying sizes, and most were circular in plan. Two structures, 16 and 30, yielded human bones. Structure 30, of roughly circular plan and at least 3 m in diameter, contained a deposit of four primary inhumations (including two adults and two infants) as well as a secondary deposit with an MNI of eight (including seven adults and one non-adult) (Magariño Sanchez 2006) (Fig. 24). Structure 30 partly cuts a V-shaped ditch, Structure 1. Abundant material was found, including typical Copper Age pottery, lithics, fragments of grinding stones and animal bones.

#### Results and Models

The radiocarbon dating at Calle Mariana de Pineda s/n concentrated on providing a robust date estimate for the construction of burial Structure 30, which cuts a ditch (Structure 1). Two previous results are available (Sac-2214 and -2216; Table 3), one from each structure, and four more results have been obtained within the ToTL project in order to refine the date estimates. The two results from Structure 1 (Sac-2214 and SUERC-53952) are from disarticulated animal bones, though sample 1.139 is a cow phalanx that was noted to have an undegraded epiphysis and so was probably protected by the epiphyseal plate when it entered the ground (that is, in a semi-fleshed state). A sample of bone from one of the articulating human individuals in Structure 30 produced Sac-2216, while a further three dates (OxA-30340, OxA-32305, and SUERC-60400) are available from teeth and bone of three articulated human individuals found in the structure. The model is relatively simple, placing the two results from Structure 1 into an unordered group and the four results from Structure 30 into a second unordered group. It then uses the relative sequence between the two structures to estimate the date when Structure 30 was constructed.

**Fig. 25:**
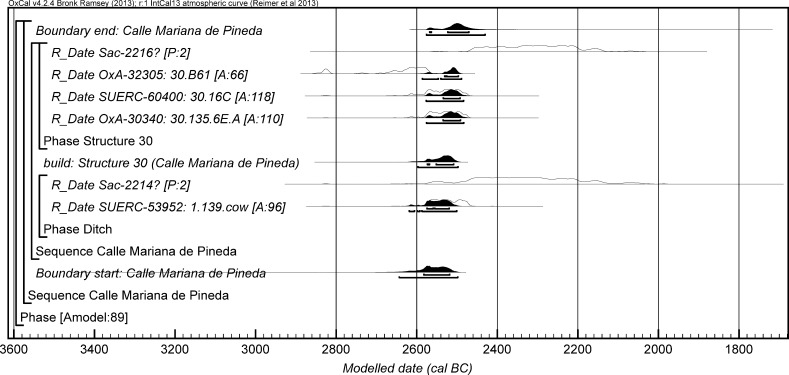
Chronological model for the activity associated with the ditch (Structure 1) and Structure 30 at Calle Mariana de Pineda s/n. The format is as described in Fig. 4. The large square ‘brackets’ down the left-hand side along with the OxCal keywords define the overall model exactly

The initial model for Calle Mariana de Pineda s/n that utilises all the radiocarbon dates and the stratigraphy between the two structures has poor agreement (Amodel = 38; model not shown). In this model, both of the results from the radiocarbon laboratory in Sacavém, Portugal (Sac-), appear to be too recent for their contexts, given the other dates and the stratigraphy. There is not enough technical information about these results to adequately critique their quality, and so they have both been excluded from the modelling. The revised model (Fig. 25) shows good overall agreement (Amodel = 89; Fig. 25).

**Fig. 26:**
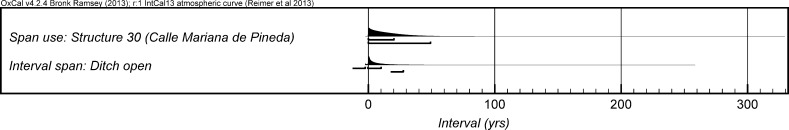
Probability distributions for the number of years over which burial activity associated with Structure 30 at Calle Mariana de Pineda s/n took place and for how long the ditch (Structure 1) may have been open. The distributions are derived from the model defined in Fig. 25

The model estimates that Structure 30 was constructed in *2600*–*2495* *cal BC* (*95%* *probability*; *build: Structure 30 (Calle Mariana de Pineda)*; Fig. 25), *2575*–*2565* *cal BC* (*3%* *probability*) or *2555*–*2505* *cal BC* (*65%* *probability*). The structure was used for *1*–*50* *years* (*95%* *probability*; *use: Structure 30 (Calle Mariana de Pineda)*; Fig. 26), probably for *1*–*25* *years* (*68%* *probability*). The burial activity in the structure ended in *2580*–*2430* *cal BC* (*95%* *probability*; *end: Calle Mariana de Pineda)*; Fig. 25), probably in *2570*–*2560* *cal BC* (*2%* *probability*) or *2525*–*2470* *cal BC* (*65%* *probability*).

It is possible, using the Interval command in OxCal, to calculate the period of time that elapsed between the deposition of the cow phalanx 1.139 in the ditch and the construction of Structure 30. The model estimates that the ditch was open for as many as *1*–*30* *years* (*95% probability*; *span: Ditch open*; Fig. 26), and probably *1*–*15* *years* (*68% probability*). It should be stressed here that we cannot be sufficiently certain of the overall temporal relationship between the deposition of the bone, the filling of the ditch, and the cutting of Structure 30 to say with confidence how long the ditch remained open. We can assume the cow bone was deposited shortly after the digging or last cleaning of the ditch and Structure 30 was constructed after the ditch was completely filled. The ditch appeared to have filled slowly (fine sand being present in the fills), perhaps with periods of faster sedimentation (levels with charred material and associated material culture).

### Calle Trabajadores N^os^ 14–18

The Calle Trabajadores N^os^ 14–18 sector is located right in the centre of Valencina, more or less in the middle of what according to some interpretations (Cruz-Auñón Briones and Arteaga Matute 1999, p. 606) would have been the ‘residential’, ‘domestic’ or ‘productive’ area of the site (Fig. 2). In 2008, and in advance of construction, the excavation of an area of c. 300 m^2^ led to the discovery of 30 negative features, predominantly circular in plan, and of varying sizes and depths (López Aldana and Pajuelo Pando 2013).

**Fig. 27:**
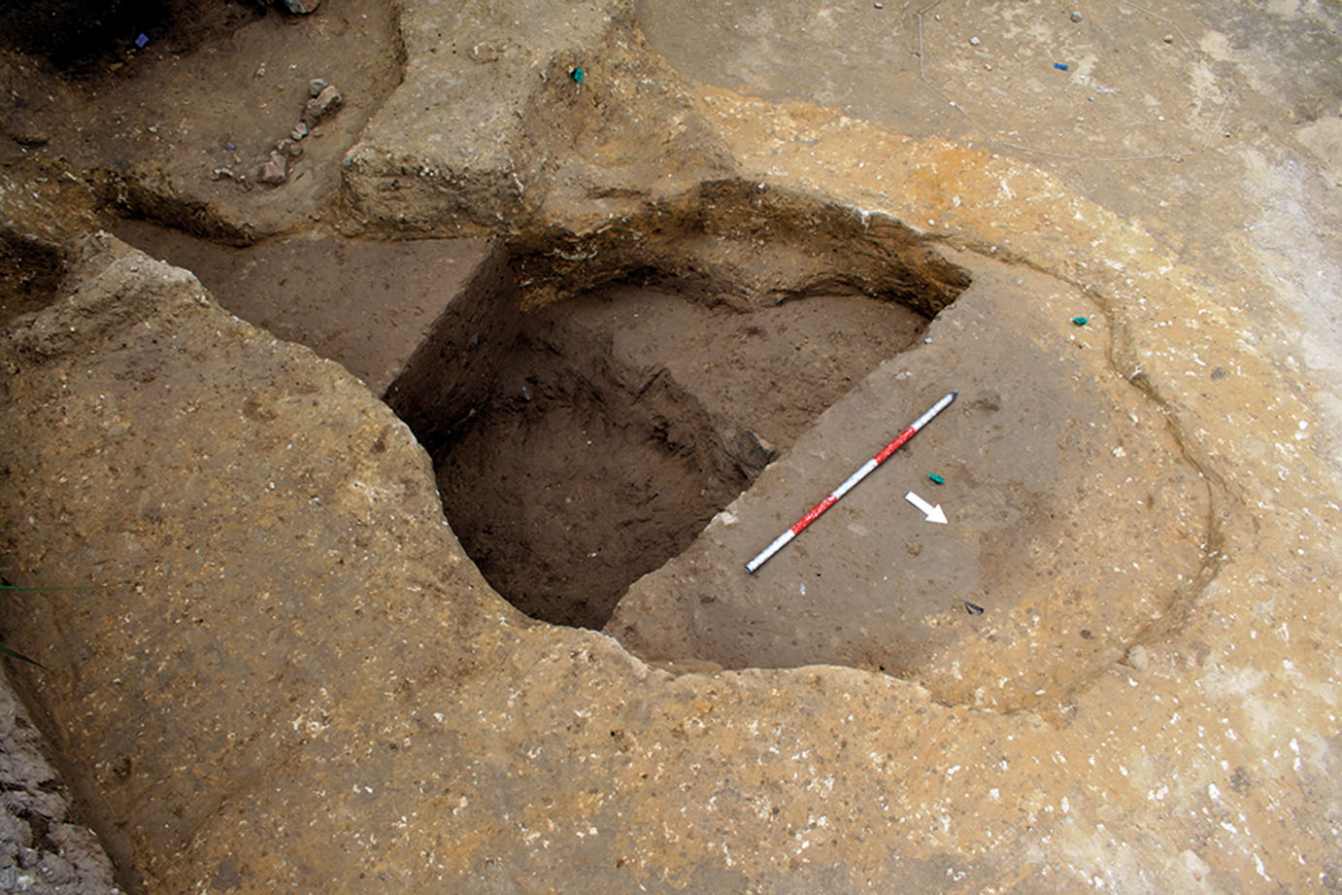
Excavation of Structure 136 at Calle Trabajadores N^os^ 14–18. Photo: Pedro López Aldana

As part of our study, radiocarbon dates have been obtained for Structures 1, 77, 90 and 136. Structure 77 was a shallow ditch, while Structure 90 was a circular, shallow pit, both containing human remains. The adjacent Structure 1 was only c. 30 cm deep (its upper part had probably been destroyed by earlier twentieth-century urban works), but with a diameter of c. 4 m. In the upper fill of Structure 1, nine human skulls were found together with articulated limbs, disarticulated axis bones, and the articulated lower front leg of a pig. Structure 1 yielded an MNI of 12, including three adults and nine sub-adults. Structure 136 was exceptionally large, 2.4 m wide at its opening and c. 4 m wide at the base, and 3.3 m deep (Fig. 27). The lower fill of Structure 136 consisted of a series of horizontal layers with little material, but its upper part contained a mixture of deposits, with a lot of material including animal bone and pottery of Copper Age forms as well as a human cranium at the top of the upper fill. Among the many elements of material culture found inside Structure 136 the excavators report that, halfway through the infill, they found a fragment of a cylinder ‘idol’ with oculi, half a plain Bell Beaker vessel c. 12 cm high, and a remarkable zoomorphic figurine portraying a bovid (López Aldana and Pajuelo Pando 2013, p. 165). As is often the case in Valencina, the specific function of Structure 136 could not be established at the time of the excavation, although it seems clear that its abandonment must have occurred when Bell Beaker pottery was already in circulation. As already noted, there was one Bell Beaker pot in this upper fill, and a further 300 Bell Beaker sherds at the top (Inácio et al. 2012, 2017; Pajuelo Pando and López Aldana 2016). Other material from Structures 1, 77, 90 and 136 included typical Copper Age pots, some lithics, and a few fragments of querns and grinding stones.

#### Results and Models

Radiocarbon dating results are available for four structures with human remains (1, 77, 90 and 136). While Structures 77, 90 and 136 have been dated with one sample each of human and animal bone, Structure 1 has a total of 11 results from two disarticulated human skulls (1.G and 1.L), three axis vertebrae of three different individuals (1.2.axis1, 1.2.axis2 and 1.2.axis3) and two articulated individuals (1.5 and 1.10). Two further results are available from samples of articulating pig and disarticulated sheep.

There are two results (OxA-30342 and SUERC-60391) on an axis (1.2.axis1) that are statistically consistent (Table 3) and have been combined prior to calibration using the R_Combine function in OxCal to form mean 1.2.axis1. The two results (OxA-30341 and SUERC-53954) from a second axis in Structure 1 (1.2.axis2) are also statistically consistent (Table 3) and have been combined to form mean 1.2.axis2. From Structure 77, there are two results (OxA-30343 and SUERC-60396) from a disarticulated sheep/goat radius (77.146.sheep1) that are statistically consistent (Table 3) and have been combined to form mean 77.146.sheep1. Finally, the two results (OxA-30379 and SUERC-60395) from a human cranium (90.155.cranium) in Structure 90 are statistically consistent (Table 3) and have been combined prior to calibration to form mean 90.155.cranium.

The model for Structure 1 only assumes that the material within the pit is all related to a general phase of use of the feature with no stratigraphic relationships between any of the samples dated. Furthermore, the four structures are all combined into a single model that makes a similar assumption that the activity associated with the dated samples represents a relatively continuous period of unknown duration. The excavators interpreted the skull (136.135.cranium) from Structure 136 as a potentially curated object, which the radiocarbon result (SUERC-53957) would appear to corroborate. Therefore, this result is excluded from the modelling as the death of the individual represented probably is not temporally related to the time of its deposition.

**Fig. 28:**
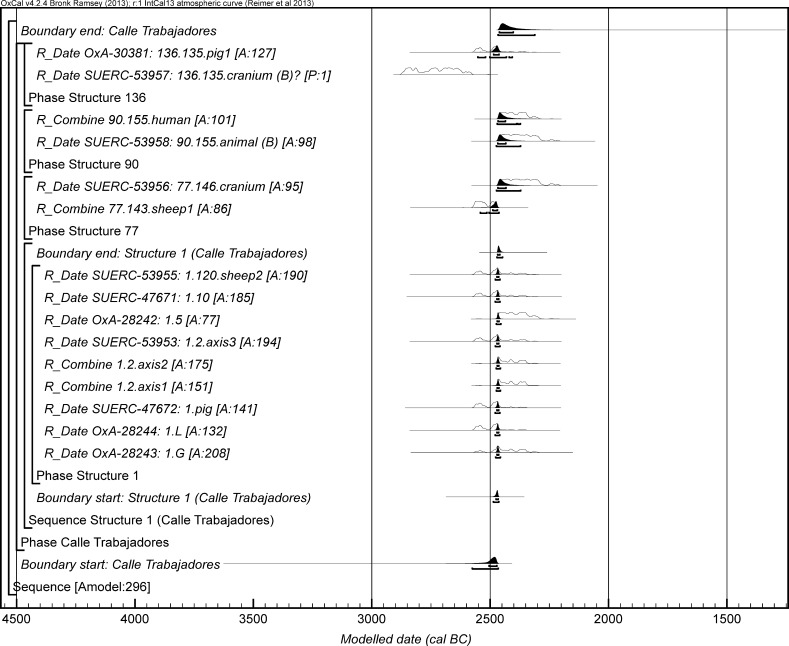
Chronological model for the activity associated with the four dated structures in the Calle Trabajadores N^os^ 14–18. The format is as described in Fig. 4. The large square ‘brackets’ down the left-hand side along with the OxCal keywords define the overall model exactly

**Fig. 29:**
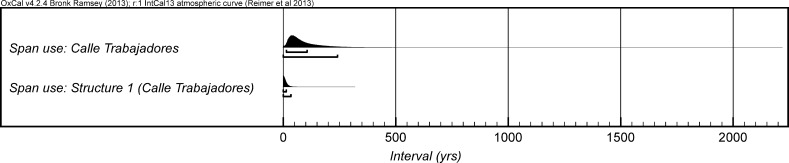
Probability distribution for the number of years over which burial activity associated with the four structures at Calle Trabajadores N^os^ 14–18 took place. The distributions are derived from the model defined in Fig. 28

The model has good agreement between the radiocarbon dates and the archaeological information (Amodel = 296; Fig. 28). It estimates that burial activity associated with all four dated structures at Calle Trabajadores N^os^ 14–18 began in *2580*–*2465* *cal BC* (*95%* *probability*; *start: Calle Trabajadores*; Fig. 28), probably in *2505*–*2470* *cal BC* (*68%* *probability*). The span of the burial activity in these structures is *1*–*245* *years* (*95%* *probability*; *use: Calle Trabajadores*; Fig. 29), probably *10*–*110* *years* (*68%* *probability*). The burial activity associated with all four structures ended in *2470*–*2310* *cal BC* (*95%* *probability*; *end: Calle Trabajadores*; Fig. 28), probably in *2465*–*2400* *cal BC* (*68%* *probability*).

The burials in Structure 1 began in *2490*–*2460* *cal BC* (*95%* *probability*; *start: Structure 1 (Calle Trabajadores)*; Fig. 28), probably in *2480*–*2465* *cal BC* (*68%* *probability*). The burials in Structure 1 ended in *2475*–*2445* *cal BC* (*95%* *probability*; *end: Structure 1 (Calle Trabajadores)*; Fig. 28), probably in *2470*–*2455* *cal BC* (*68%* *probability*). The burials in Structure 1 spanned *1*–*35* *years* (*95%* *probability*; *use: Structure 1 (Calle Trabajadores)*; Fig. 29), probably *1*–*15* *years* (*68%* *probability*).

After excluding the potentially curated skull (136.135.cranium), all the radiocarbon measurements are statistically consistent (T′ = 24.2; T′ (5%) = 27.7; ν = 17) and could be the same radiocarbon age. Therefore, the interpretation by the excavators that the activity in this area might have been a single event is compatible with the radiocarbon results.

## Other Dated Monuments and Features

Alongside the dates obtained in collaboration with the ToTL project for the features and monuments from the sectors described above, on the basis of samples selected by the rigorous criteria already noted, a number of other unpublished dates are available from other features and structures, gathered principally in the course of rescue excavations over the last three decades. Some are on less than ideal samples, and numbers of samples are often low, so there is a clear methodological lesson here for future dating programmes. Nonetheless, these measurements and the associated evidence contribute to refining the chronology of Copper Age Valencina de la Concepción and to reassessing its overall character. In the case of the Plan Parcial Matarrubilla sector, already published dates (Nocete Calvo et al. 2008) are formally modelled.

### Calle Ruiseñor N^o^ 20

**Fig. 30:**
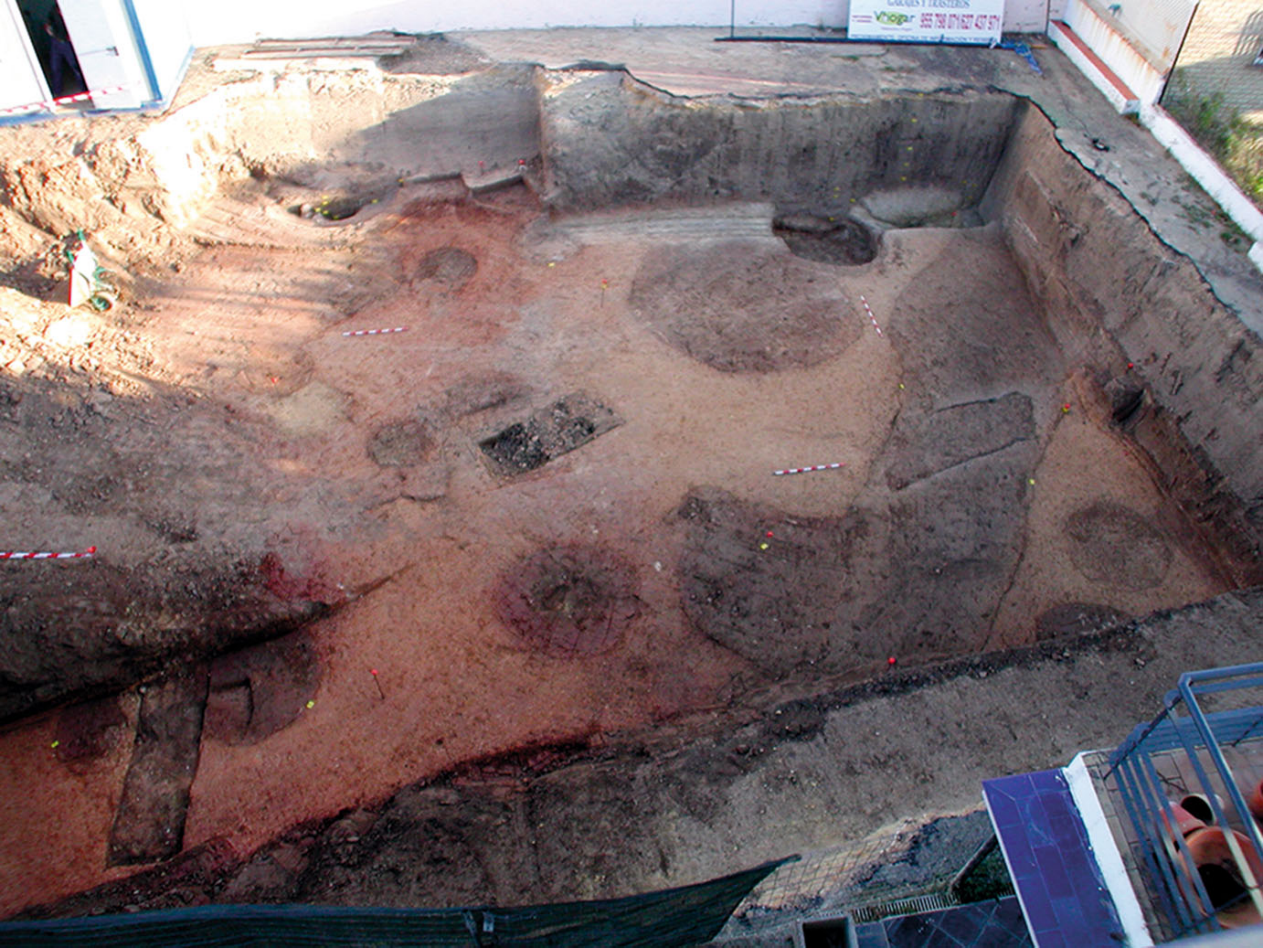
Overview of excavation at Calle Ruiseñor N^o^ 20. Photo: Miguel Ángel de Dios Pérez

The Calle Ruiseñor Nº 20 sector is located in the central part of the site, some 500 m to the southeast of Calle Trabajadores N^os^ 14–18 (Fig. 2). Excavation in advance of development carried out between September and November 2007, which was not followed by any post-excavation study and remains unpublished, led to the discovery of 20 structures, including seven large pits, ten smaller ones, two shafts and one ditch; the excavator interpreted the large and small pits as ‘hut-floors’ and ‘silos’ respectively (De Dios Pérez 2008) (Fig. 30). Pit 62 was interpreted as a ‘silo’ that had later been re-used as a burial pit for an individual inhumation. UE 67, a negative feature with a poly-lobulate plan, was interpreted as a hut floor with a red-clay floor, an inner ‘silo’ and a small ‘bench’. Another structure (number not specified) was interpreted as a poorly preserved hut-floor that showed a segment of a circle made with sandstone and slate. Structure 10 (also interpreted as a ‘hut-floor’) included a series of post-holes to support the roof. Structure 13 was a V-section ditch 3 m deep; two samples of charred material from this ditch were obtained and then radiocarbon dated. Among the finds, which were remarkably scarce, the excavator noted a single pot, very few bone tools and three stone arrowheads.

There are six radiocarbon results on unidentified charcoal from a range of contexts associated with deposits in the ditch (CNA-811 and -817), one of the ‘huts’ (CNA-812, -816 and -818) and a ‘silo’ (CNA-815) (Table 2). Although the excavator has attached phasing to each measurement, the material was unidentified and the descriptions of the contexts do not allow for a robust assessment of the taphonomic relationship between samples and contexts, so no stratigraphic relationships have been modelled. Furthermore, Pit 62 (one of the ‘silos’) was interpreted as being reused at a later date, and the radiocarbon date (CNA-815) from this feature is considerably later than the remaining dates. Erring on the side of caution, this result has been excluded from the modelling, as it might be related to the reuse and not the primary activity considered here. In an attempt to account for the possibility that the dated material included some inbuilt age, we have employed the exponential charcoal outlier model proposed by Dee and Bronk Ramsey (2014) (Outlier_Model (“Charcoal”,Exp(1,-10,0),U(0,3),”t”); with a prior probability of 1 for each measurement on a sample of unidentified charcoal). We also assume that the dated material derives from separate events, rather than a single event with charcoal redistributed throughout the site.

**Fig. 31:**
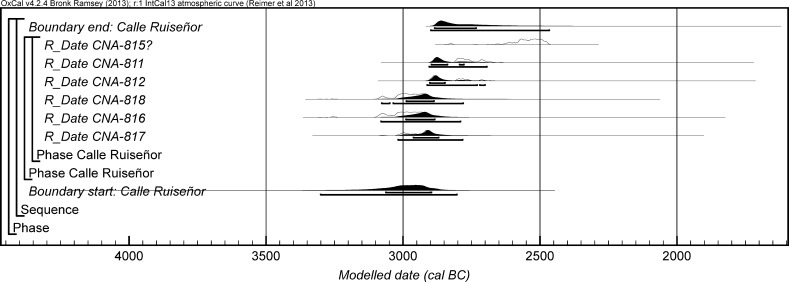
Chronological model for the activity at the Calle Ruiseñor N^o^ 20. The format is as described in Fig. 4. The large square ‘brackets’ down the left-hand side along with the OxCal keywords define the overall model exactly

**Fig. 32:**
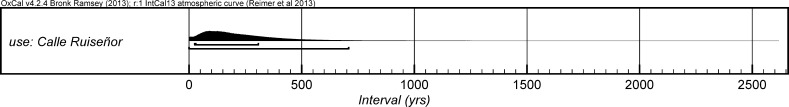
Probability distribution for the number of years over which activity at Calle Ruiseñor N^o^ 20 took place. The distribution is derived from the model defined in Fig. 31

This model is shown in Fig. 31. It estimates that the activity in this sector began in *3305*–*2800* *cal BC* (*95%* *probability*; *start: Calle Ruiseñor*; Fig. 31), probably in *3065*–*2895* *cal BC* (*68%* *probability*). The activity ended in *2900*–*2465* *cal BC* (*95%* *probability*; *end: Calle Ruiseñor*; Fig. 31), probably in *2885*–*2730* *cal BC* (*68%* *probability*). The overall span of activity represented is *1*–*710* *years* (*95%* *probability*; *use: Calle Ruiseñor*; Fig. 32), probably *25*–*310* *years* (*68%* *probability*). (A model which assumes that the dated charcoal at Calle Ruiseñor did not have a large inbuilt age was constructed as a sensitivity analysis. This approach shifts the posterior distribution for start of activity at Calle Ruiseñor earlier by less than a decade, but shifts the posterior distribution for the end of activity on the site earlier by about 40 years.)

### El Algarrobillo

**Fig. 33:**
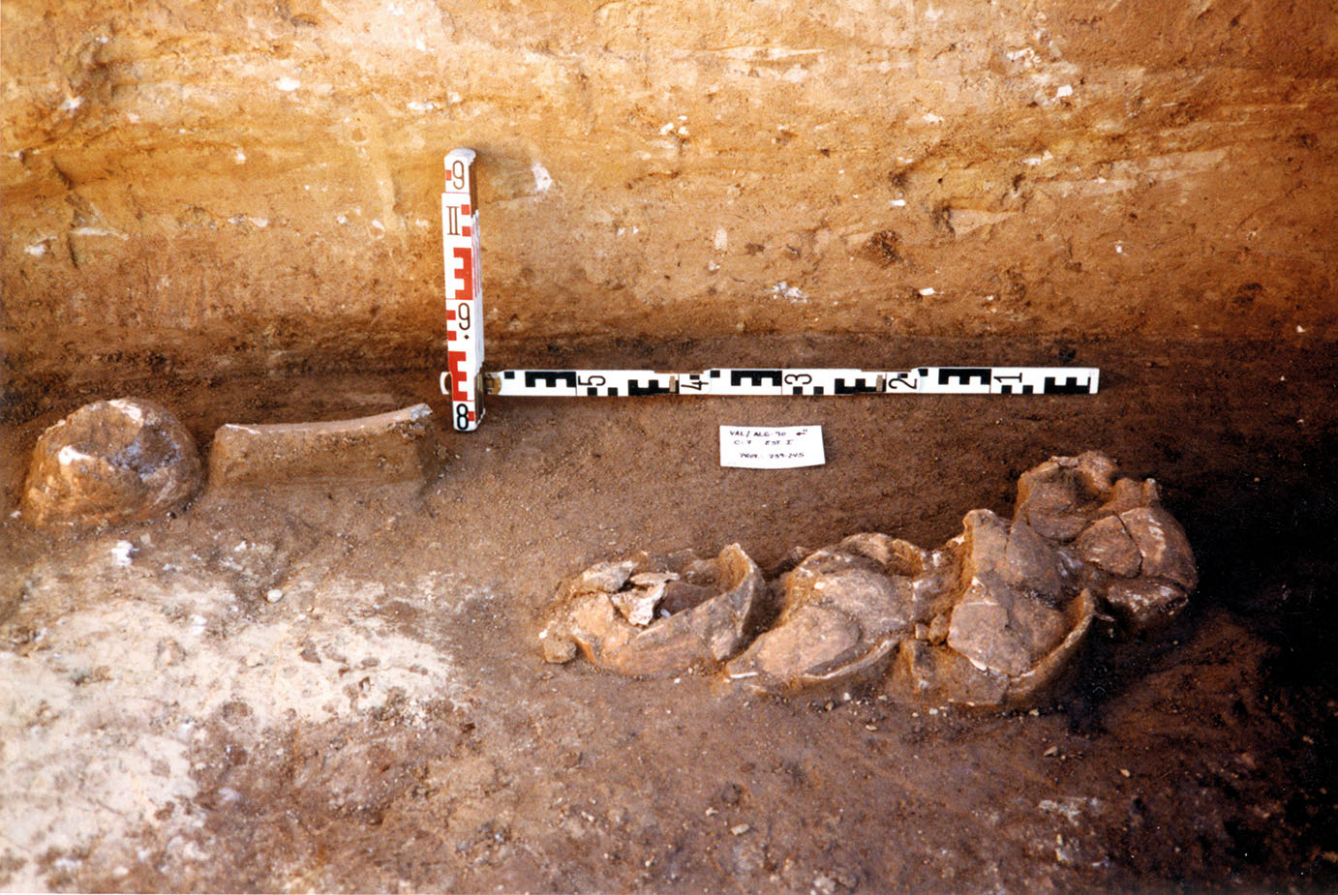
El Algarrobillo: human skulls in Structure 1. Photo: Isabel Santana Falcón

Excavations in this sector, located roughly on the central-western side of the site (Fig. 2), took place in the early 1990s following geophysical survey (Santana Falcón 1993). A variety of cut features (Costa Caramé et al. 2010, p. 90) and an MNI of 19 were found, including six young adults and eight adults (Díaz-Zorita Bonilla 2017, p. 96) (Fig. 33).

One of the features (Structure 1) was circular in plan (4 m in diameter) and 1.35 m deep. The remains of nine individuals were found in the fill. At the base, there was a level of compacted earth, over which there was a hearth. There were two skulls to the south of the hearth, and another to the northeast, associated with a pelvis, two femurs, ribs and phalanges; a fourth skull to the northwest was associated with a carinated bowl and a cow jaw. Five further human skulls lay at a higher level. There were also pottery sherds, six flint blades, slag, two fragments of metallic artefacts and abundant faunal remains.

Feature E.I consisted of two circular negative structures connected by a corridor with three niches. The whole structure was oriented NE–SW, 3.4 m long and up to 0.9 m wide. One of the niches contained a pot and a large flint blade. In front of it, in the corridor, were the remains of a secondary adult inhumation. One of the negative structures held the remains of a minimum of six individuals, consisting of skulls and long bones, some of them articulated. Associated with these were various fragments of polished stones and sherds of a pottery plate.

Feature E.II, 1 m in diameter and 0.35 m deep, contained faunal remains and pottery fragments from forms typical for the Copper Age. Another circular pit only 0.9 m deep contained abundant pottery and bone fragments, as well as two fragmentary grinding stones.

**Fig. 34:**
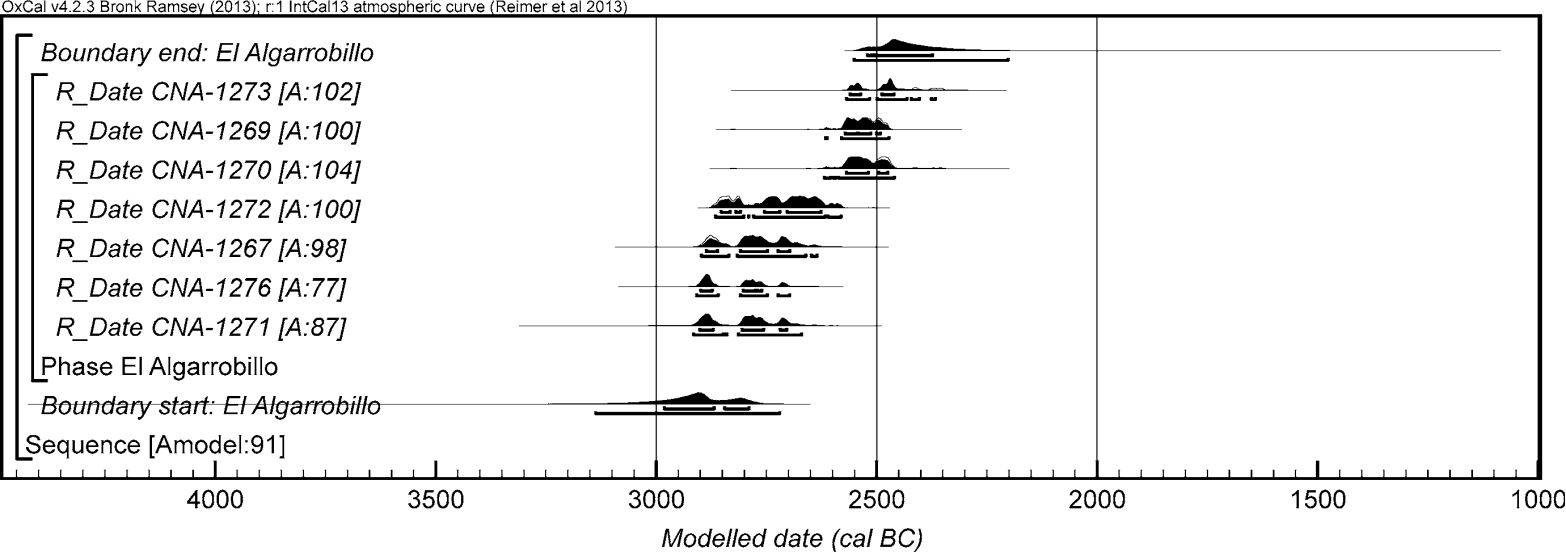
Chronological model for activity in the El Algarrobillo sector. The format is as described in Fig. 4. The large square ‘brackets’ down the left-hand side along with the OxCal keywords define the overall model exactly

**Fig. 35:**
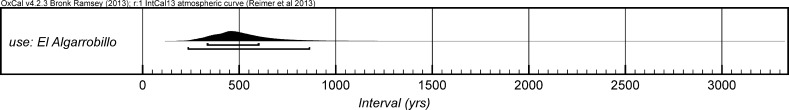
Probability distribution for the number of years over which activity in El Algarrobillo took place. The distribution is derived from the model defined in Fig. 34

A total of seven radiocarbon results are available on bone from seven individuals (Table 2). The chronological model has no stratigraphic relationships between the samples and only assumes that the deposition of the individuals occurred relatively uniformly over a period of unknown duration. The model (Fig. 34) has good agreement with the radiocarbon dates (Amodel: 91). It estimates that the activity associated with Structure 1 began in *3140*–*2720* *cal BC* (*95%* *probability*; *start: El Algarrobillo*; Fig. 34), probably in either *2985*–*2865* *cal BC* (*51%* *probability*) or *2845*–*2785* *cal BC* (*17%* *probability*). The activity ended in *2555*–*2200* *cal BC* (*95%* *probability*; *end: El Algarrobillo*; Fig. 34), probably in *2525*–*2370* *cal BC* (*68%* *probability*). The activity spanned *235*–*865* *years* (*95%* *probability*; *use: El Algarrobillo*; Fig. 35), probably *330*–*605* *years* (*68%* *probability*).

**Fig. 36:**
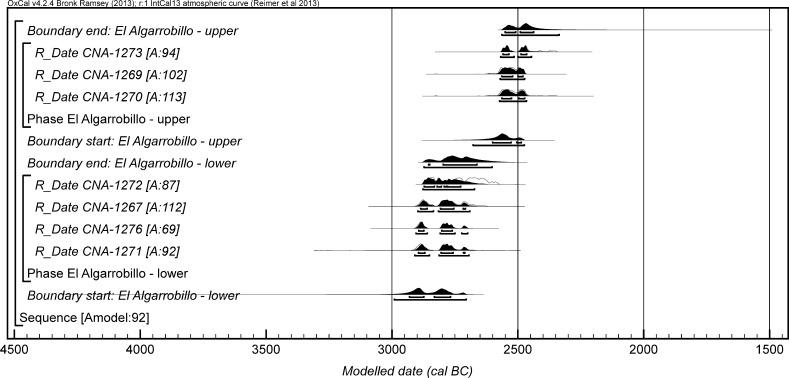
Alternative chronological model for activity in the El Algarrobillo sector, assuming two distinct phases of activity. The format is as described in Fig. 4. The large square ‘brackets’ down the left-hand side along with the OxCal keywords define the overall model exactly

**Fig. 37:**
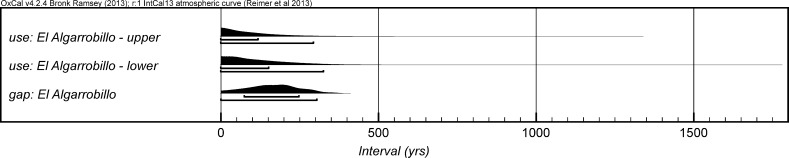
Probability distributions for the number of years over which activity for the two potential phases at El Algarrobillo took place and for the gap between them. The distributions are derived from the alternative model defined in Fig. 36

An alternative model that places CNA-1267, 1271, -1272 and -1276 in the lower level and CNA-1269, -1270 and -1273 in the upper level has good agreement (Amodel: 92; Fig. 36). This alternative model estimates that the activity associated with the lower level of Structure 1 began in *2995*–*2705* *cal BC* (*95%* *probability*; *start: El Algarrobillo*—*lower*; Fig. 36), probably in either *2935*–*2870* *cal BC* (*33%* *probability*) or *2835*–*2765* *cal BC* (*35%* *probability*). The lower level activity ended in *2875*–*2600* *cal BC* (*95%* *probability; end: El Algarrobillo*—*lower*; Fig. 36), probably in either *2855*–*2850* *cal BC* (*1%* *probability*) or *2800*–*2660* *cal BC* (*67%* *probability*). The overall span of the activity in the lower level was *1*–*330* *years* (*95%* *probability; use: El Algarrobillo*—*lower*; Fig. 37), probably *1*–*155* *years* (*68%* *probability*).

The activity associated with the upper level of Structure 1 began in *2680*–*2470* *cal BC* (*95%* *probability*; *start: El Algarrobillo*—*upper*; Fig. 36), probably in either *2605*–*2525* *cal BC* (*60%* *probability*) or *2505*–*2485* *cal BC* (*8%* *probability*). The activity ended in *2565*–*2330* *cal BC* (*95%* *probability*; *end: El Algarrobillo*—*upper*; Fig. 36), probably in either *2555*–*2505* *cal BC* (*27%* *probability*) or *2495*–*2435* *cal BC* (*41%* *probability*). The overall span of the activity in the upper level was *1*–*295* *years* (*95%* *probability*; *use: El Algarrobillo*—*upper*; Fig. 37), probably *1*–*120* *years* (*68%* *probability*).

In this scenario, there may have been a gap of *1*–*305* *years* (*95%* *probability*; *gap: El Algarrobillo*; Fig. 37), probably of *70*–*250* *years* (*68%* *probability*) between phases of burial in Structure 1.

Given the lack of information about the provenance of the dated samples from this sector, the first model is perhaps to be preferred, simply because it is more conservative.

### La Alcazaba

The sector of La Alcazaba, located broadly in the centre of the site (Fig. 2), was excavated in 1996 (Cruz-Auñón and Arteaga Matute 1996). According to the excavators, two of the three pits found (Structures 18 and 19), were of Copper Age date, while the third one belonged to the Early Bronze Age.

Structure 18 was a bell-shaped pit c. 0.5 m deep and 1.33 m in maximum diameter, in which some fragments of material culture were found. Structure 19 was a pit (0.53 m deep and 1.7 m in diameter) containing commingled human remains mixed with faunal remains and ceramics. The MNI was seven: one subadult (Infant I) and six adults (four young adults, one of 20–30 and one of 30–40 years of age) (Díaz-Zorita Bonilla 2017, p. 53).

The right humerus was dated from four of the individuals in Structure 19 (Table 2). Since the remains were commingled, they were simply treated as belonging to a phase of relatively continuous activity with an unknown duration. Since the remains were commingled and were recovered disarticulated, we cannot be absolutely certain that these individuals were placed into the structure shortly following their death. However, the radiocarbon dates do form a tight grouping and are statistically indistinguishable (T′ = 3.3; T′ (5%) = 7.8; ν = 3), which might suggest that their deaths occurred very closely in time.

**Fig. 38:**
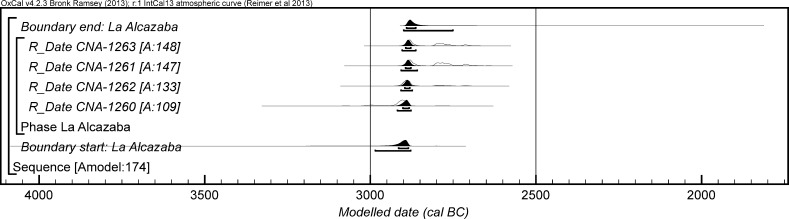
Chronological model for activity in the La Alcazaba sector. The format is as described in Fig. 4. The large square ‘brackets’ down the left-hand side along with the OxCal keywords define the overall model exactly

**Fig. 39:**
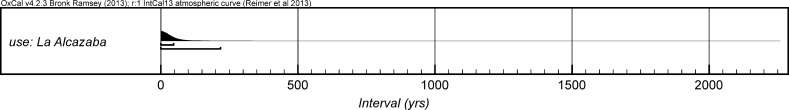
Probability distribution for the number of years over which activity at La Alcazaba took place. The distribution is derived from the model defined in Fig. 38

The radiocarbon dates have good agreement with the model (Amodel: 174; Fig. 38). Assuming that the bodies entered the structure shortly after death, the model estimates that burial began in *2985*–*2875* *cal BC* (*95%* *probability*; *start: La Alcazaba*; Fig. 38), probably in *2915*–*2885* *cal BC* (*68%* *probability*). The burials ended in *2900*–*2750* *cal BC* (*95%* *probability*; *end: La Alcazaba*; Fig. 38), probably in *2895*–*2860* *cal BC* (*68%* *probability*). The overall span of burial was *1*–*220* *years* (*95%* *probability*; *use: La Alcazaba*; Fig. 39), probably *1*–*50* *years* (*68%* *probability*).

### PP-Matarrubilla

This sector is in the centre of the site (Fig. 2). Between 2002 and 2004 rescue excavations covering almost 9 ha (88,000 m^2^) documented a broad variety of features. There were 198 negative structures concentrated in four groups (Sectors I–IV), some with remains of adobe, scattered to the north of the E–W ditch. Small circular pits were filled with what the excavator interprets as ‘garbage’ (Queipo de Llano Martínez, 2010, p. 3210). Larger poly-lobulate structures (3–4 m diameter and 1 m deep) contained faunal remains, marine molluscs and carbonised cereals and legumes. In the southern part, there was a 500 m-long segment of a ditch running E–W with a V section (3–7 m deep and 4.6–6 m wide). Both a context at the base of this ditch and its uppermost fill were later interpreted as smelting rubbish dumps, the latter associated with seven tuyeres (Nocete Calvo et al. 2008). Two other 150 m-long ditch segments, rectilinear and perpendicular to the previous one, were also found. The remaining negative structures recorded at the sector (some 84% of the total, of varying shapes and sizes, but usually not more than 0.6 m deep) all showed ‘strongly thermally altered walls and bases’ (Nocete Calvo et al. 2008, p. 719). These contained minerals, copper slag, remains of tools related to metallurgical activities, and what have been interpreted as ‘smelting furnaces’ (Nocete Calvo et al. 2008).

In Sector IV there was evidence for diverse activity. There was storage of minerals, grinding and reduction of minerals, and reduction ‘furnaces’. Overall 14,224 g of copper minerals were recorded. There were combustion structures (furnaces) with abundant slag (overall 16,395 g). Copper smelting is proposed, on the basis of 185 crucibles, as well as moulds and tongs. Thirteen copper objects, including knives, punches, needles, hooks and saws, were found, and there were stone tools said to be related to the manufacturing process (Nocete Calvo et al. 2008). Metallurgical activity and its confinement to the north of the long ditch, plus an alleged absence of subsistence or food-preparation activities, led Nocete Calvo et al. (2008) to suggest that this was a large ‘smelting quarter’.

There are 18 published radiocarbon dates for this sector (Table 2). Samples for 11 determinations were identified as *Quercus ilex* charcoal, with the remaining samples identified as sheep/goat bone. Seven of the charcoal samples were obtained on fragments of charcoal that were embedded within copper slag. To account for the possibility that the dated fragments of oak charcoal included some inbuilt age, we have employed the exponential charcoal outlier model proposed by Dee and Bronk Ramsey (2014) (Outlier_Model(“Charcoal”,Exp(1,-10,0),U(0,3),”t”); with a prior probability of 1 for each measurement on a charcoal sample). Two dates (Ua-36023 and -36043) are more than 200 years later than the latest of the remaining 16 dates. This raises two scenarios. Either the 16 samples that produced a coherent set of dates are all residual, by as much as 500 years, leaving the later samples to accurately date the site, or those two dates are from more recent activity. The model was constructed on the basis of the latter scenario, and the two later results are excluded.

**Fig. 40:**
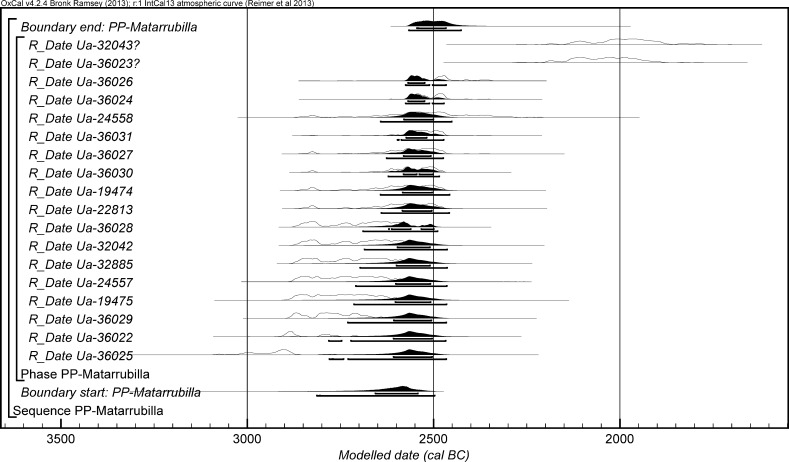
Chronological model for activity in the PP-Matarrubilla sector. The format is as described in Fig. 4. The large square ‘brackets’ down the left-hand side along with the OxCal keywords define the overall model exactly

**Fig. 41:**
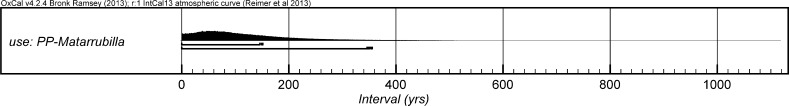
Probability distribution for the number of years over which activity at PP-Matarrubilla took place. The distribution is derived from the model defined in Fig. 40

There is no stratigraphic relationship between any of the samples, so they are modelled as simply relating to a relatively continuous phase of activity. The model is shown in Fig. 40, and estimates that the activity at PP-Matarrubilla began in *2815*–*2495* *cal BC* (*95%* *probability*; *start: PP*-*Matarrubilla*; Fig. 40), probably in *2660*–*2540* *cal BC* (*68%* *probability*). The dated activity ended in *2570*–*2425* *cal BC* (*95%* *probability*; *end: PP*-*Matarrubilla*; Fig. 40), probably in *2545*–*2465* *cal BC* (*68%* *probability*). The overall span for the modelled activity is *1*–*360* *years* (*95%* *probability*; *use: PP*-*Matarrubilla*; Fig. 41), probably *1*–*155* *years* (*68%* *probability*). (A model which assumes that the dated oak charcoal at PP-Matarrubilla did not have a large inbuilt age was constructed as a sensitivity analysis. This approach strongly affects the posterior distributions produced by the model. It shifts the posterior distribution for the beginning of activity at PP-Matarrubilla earlier by over 200 years, and the estimate for the end of activity here later by two or three decades. Without botanical identification of the age as well as the species of the dated material, it is, of course, possible that all the dated charcoal at Plan Parcial Matarrubilla consisted of twigs, but the results from the exponential charcoal outlier model illustrated in Fig. 40 suggest that this is unlikely. The difference between the alternative models is stark: persistent activity covering 300 or 400 years over much of the first half of the third millennium cal BC, or activity covering a century or so centring on the 26th century cal BC. Modelling the possibility of inbuilt age in wood samples is possible, but full botanical identification and the selection of short-lived material for dating are much to be preferred.)

### La Gallega

The La Gallega sector, located in the northeast part of the site (Fig. 2), was excavated in 1990–1991 (Martín Espinosa and Ruiz Moreno 1992). Twenty-three negative structures with depths of around two metres and varied morphologies were recorded. One group is composed of a series of circular structures with depths of 1.5–2 m, showing semi-circular or inverted bell sections, flat or concave bases, and filled with faunal remains and pottery sherds. A second group of pits show bucket-like shapes, 1 m in diameter, and flat bases. There was a third group of shallow structures of smaller dimensions (c. 0.2–0.5 m diameter), which occasionally appear joined to each other or to other structures. Pit 10, showing an elongated plan orientated N–S, with rounded ends, slightly bent walls, a flat base, and two distinct levels of infill, yielded two individuals, an old male and an infant (c. 7 years) (Alcázar Godoy et al. 1992, p. 23; Díaz-Zorita Bonilla 2017, p. 96). A broad variety of ceramic forms were recovered (plates, platters and pots), as well as clay loomweights, lithic artefacts (flint arrowheads and small blades, two polished axes and fragments of grinding stones, among others), a few bone artefacts (pin, punches and spatula), and copper artefacts (among others two knives, a blade fragment, a small flat axe, pins, punches, and various fragments of slag). Additionally, two ‘idols’, one made of bone and a plaque, faunal remains, as well as building material, were recovered.

**Fig. 42:**
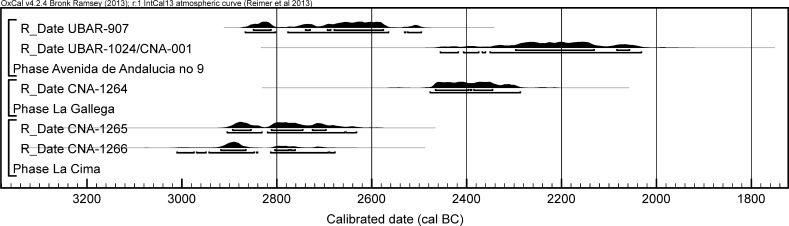
Probability distributions for the simple calibrated dates from La Cima, La Gallega and Avenida de Andalucía N^o^ 9

There is a single radiocarbon result (CNA-1264; 3905 ± 35 BP) from the sector, from a fragment of human occipital bone recovered in Pit 10. The result calibrates to 2480–2285 cal BC (95% probability; Stuiver and Reimer 1993; Fig. 42; Table 2).

### La Cima

The sector of La Cima is roughly in the middle of the site (Fig. 2). Excavation was carried out in 1989–1990 (Ruiz Moreno 1991) and consisted of eight trenches measuring 4–16 m^2^, some of which were enlarged at a later stage of the intervention.

Trenches 1–4, 5, 7 and 8 provided almost no material culture, with the exception of rock-crystal artefacts recovered from Trench 8 (cores, debris and small blades) and coarse pottery sherds from Trench 5, where a pebble-stone pavement was also detected. In Trench 6, in the centre of a patch of dark earth, a skull and a number of disarticulated bones belonging to an infant (Alcázar Godoy et al. 1992) and large quantities of pottery sherds were recorded. From Level 4 on, the trench provided large quantities of pottery and lithic artefacts, as well as osteological remains, large fragments of slate, and clay lumps. Consequently, it was subdivided into five areas (A–E). In Sub-areas E and D there were the remains of a pair of flexed legs and a pelvis (sub-area E), and a skull, ribs and arms (sub-area D), all belonging to the same individual (a young adult woman between 18 and 25 years of age) (Alcázar Godoy et al. 1992; Díaz-Zorita Bonilla 2017, p. 59). The human remains and large fragments of slate might conceivably be the remains of a possible megalithic or semi-megalithic construction that could have passed unrecognised during the excavation (García Sanjuán and Díaz-Zorita Bonilla 2013, p. 394).

There are two radiocarbon dates (CNA-1265 and -1266; Table 2) on human bone from Level 9 of Structure C-6. The material dated included a skull and left humerus from two different individuals. The calibrated results (Fig. 42) suggest that the associated activity dates to some point in the 29th or 28th century cal BC.

### Avenida de Andalucía N^o^ 9

This plot, some 800 m^2^ in extent, lies in the middle of the northern part of the site (Fig. 2). It was excavated in 2006 in advance of development (Sardá Piñero 2013). Three large parallel ditches running E–W were found. The widest ditch was excavated to a depth of 1.8 m, but its base was not reached. A sample of unidentified bulk charcoal from a depth of 1.8 m was dated to 2460–2415 cal BC (3% probability; UBAR-1024; Stuiver and Reimer 1993; Fig. 42; Table 2) or 2410–2360 cal BC (4% probability), or 2355–2030 cal BC (88% probability), probably in 2300–2130 cal BC (62% probability) or 2085–2055 cal BC (6% probability).

These ditches enclose or cut a series of other features. Circular structure CUE39, 1.2 m in diameter at its top and almost 2 m lower down, was only excavated to a depth of c. 1 m. Material included bone pins and needles, arrowheads, clay loomweights, faunal remains (including an antler of a young deer) and a betyl idol. A bone sample from the infill (UE 156, 06/44/156) was dated to 2870–2800 cal BC (20% probability; UBAR-907; Stuiver and Reimer 1993; Fig. 42; Table 2) or 2780–2560 cal BC (70% probability), or 2535–2495 cal BC (5% probability), probably in 2850–2810 cal BC (15% probability) or 2740–2725 cal BC (3% probability), or 2695–2685 cal BC (2% probability), or 2680–2575 cal BC (48% probability).

There were other features of varying size. Some contained numerous clay lumps and adobe fragments, many with wattle imprints. Overall they produced abundant faunal remains, mainly of pig (some with signs of burning) but also of bovids and ovicaprids. Structure CUE29, a poly-lobulate feature cut to various depths in the local marls and with a maximum diameter of c. 5 m, had a fragmented grinding stone, a large pottery plate, a pot, abundant faunal remains, crescents and horns (idols?), lithic blades and arrowheads. At the base of structure CUE 45 there was an ‘idol’. Another feature had three human skulls (one from a male of undefined age, and another from a young female adult). Associated with these skulls there were animal bones, mainly from very young individuals (long bones of ovicaprines, one pig, and a tooth of a herbivore).

The two radiocarbon results obtained from this sector (Sardá Piñero 2013, p. 153) are very different. The bone sample (UBAR-907) dates to the first half of the third millennium cal BC, while the bulk charcoal date (UBAR-1024/CNA-001) calibrates across most of the second half of the third millennium cal BC.

### Cerro de la Cabeza

The probable tholos of Cerro de la Cabeza is near the top of a hill in the northernmost sector of Valencina (Fig. 2). After its extensive destruction in 1974 through soil extraction works for the construction of a nearby road, a rescue excavation was carried out (Fernández Gomez and Ruiz Mata 1978).

The largely destroyed megalithic structure showed only half of a small, circular chamber (2 m in diameter) excavated into the subsoiland and lined with slate slabs (c. 80–90 cm high). Despite its denomination as ‘tholos’, no conclusive evidence for the roofing system of the chamber could be gathered. Excavations in the chamber recovered scant human remains (a small fragment of a long bone and a premolar of a young individual); a few lithic artefacts (two flint arrowheads, two flint blades, two jasper arrowheads); a bone needle; 12 complete or almost complete pieces of pottery of typical Copper Age form; and two plates with burnished decoration, which is typically attributed to the Late Bronze Age in the Guadalquivir valley. Numerous pottery sherds were also collected from around the destroyed remains of the tomb, and a decorated slate plaque with oculus motifs—now the official icon of modern-day Valencina de la Concepción—was also recovered by the workers during the process of soil extraction at the tomb site.

**Fig. 43:**
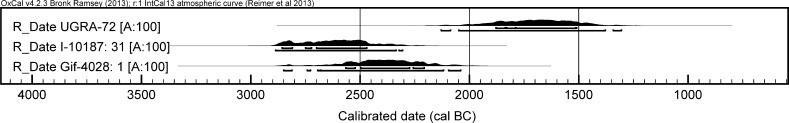
Probability distributions for the simple calibrated dates that are not modelled from Cerro de la Cabeza

The added interest of this sector is that a series of ‘idols’ of varied typology were recovered from the shafts and pits located in the area and excavated in 1976. Prominent among these are two anthropomorphic bone ‘idols’, a cylinder idol and a phalanx idol collected from Shaft 1 (6–10 m deep), two phalanx idols located in Shaft 31, and six ‘horned’ idols documented in various pits (Fernández Gómez and Oliva Alonso 1980). Two radiocarbon dates were obtained from charcoal retrieved from the lowest level of Shaft 1 (Gif-4028; Table 2) and the uppermost level of Shaft 31 (I-10187; Table 2) (Ruiz Mata and Oliva Alonso 1980, p. 43). These calibrate to the third millennium cal BC, while a third unprovenanced and very poorly documented result (UGRA-72; Table 2) dates to the second millennium cal BC (Fig. 43).

**Fig. 44:**
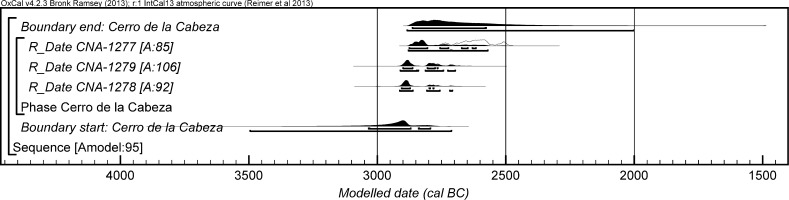
Chronological model for activity in the Cerro de la Cabeza sector. The format is as described in Fig. 4. The large square ‘brackets’ down the left-hand side along with the OxCal keywords define the overall model exactly

**Fig. 45:**
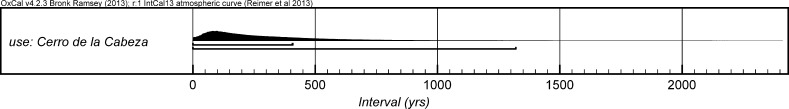
Probability distribution for the number of years over which activity at Cerro de la Cabeza took place. The distribution is derived from the model defined in Fig. 44

There are three radiocarbon results (CNA-1277–9) on human bone from Structure F1 and Ditches 1 and 2. These have been put into a simple chronological model which assumes that they belong to a phase of related activity. The model has good agreement (Amodel: 95) and estimates the related activity began in *3495*–*2710* *cal BC* (*95%* *probability*; *start: Cerro de la Cabeza*; Fig. 44), probably in either *3035*–*2865* *cal BC* (*61%* *probability*) or *2840*–*2790* *cal BC* (*7%* *probability*). The activity ended in *2885*–*2000* *cal BC* (*95%* *probability*; *end: Cerro de la Cabeza*; Fig. 44), probably either in *2865*–*2575* *cal BC* (*68%* *probability*). The activity spanned *1*–*1325* *years* (*95%* *probability*; *use: Cerro de la Cabeza*; Fig. 45), probably *1*–*410* *years* (*68%* *probability*). The low precision of the model outputs is directly attributable to the extremely low number of results in the model (Steier and Rom 2000).

### La Pastora

**Fig. 46:**
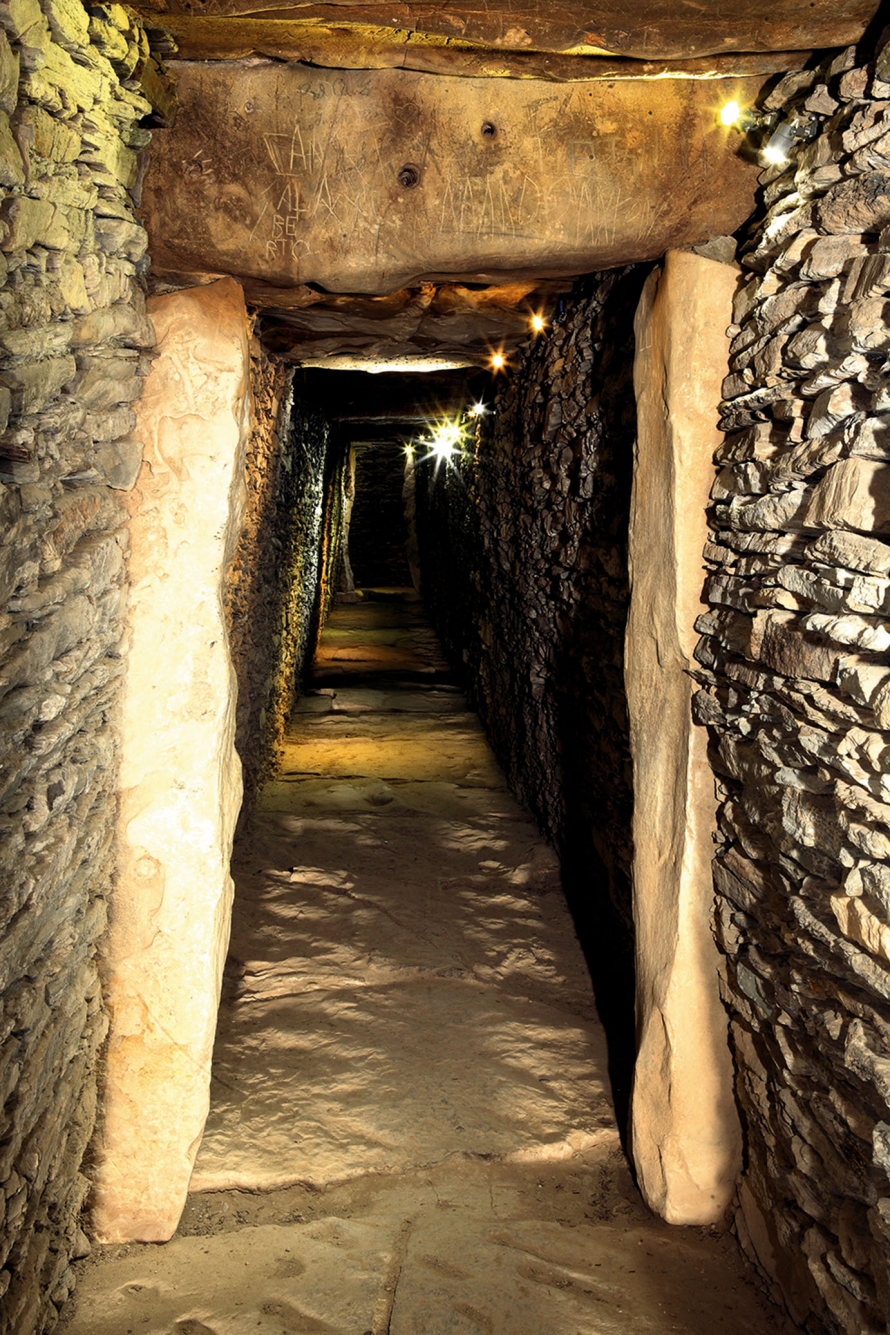
La Pastora corridor. Photo: Miguel Ángel Blanco de la Rubia by courtesy of the Cultural Board of the Andalusian Regional Government

Discovered in 1860, La Pastora is one of the most important megalithic monuments in Iberia. It consists of an unusually long corridor, approximately 42 m in length, leading to a small circular chamber, 2.5 m wide and 3 m high. The walls are of dry-stone masonry (sandstone and shale) with larger slabs forming the corridor roof while the chamber is covered by a corbelled roof (‘false dome’) (Fig. 46). A recent paper provides a good account of the research history of this monument (Ruiz Moreno 2013). Geophysical survey of its immediate vicinity has shown many features, including some large ditches (Vargas Jiménez, Meyer and Ortega Gordillo 2012). Its building materials have been geologically characterised, and attempts have been made to date its construction through radiocarbon analysis of the marine shells present in some of the corridor’s capstones (Cáceres Puro et al. 2014). A remarkable characteristic of La Pastora’s design is its anomalous astronomical orientation, which at 243° faces southwest, unlike the majority of southern Iberian megalithic monuments, which face sunrise (Hoskin 2001). This has interesting implications both in terms of chronology and the evolution of the megalithic architecture at Valencina, which are discussed further below.

Little is known about the human remains or the material culture left in this monument while it was in use. Francisco María Tubino, the first person to study it, donated a small number of artefacts to the Spanish National Archaeological Museum in Madrid, including three small gold foils, two beads (one amber, one variscite) as well as 17 copper spearheads that he claimed were found inside or around the monument. To the Seville Archaeological Museum he donated another 12 copper spearheads. The study of these exceptional spearheads suggests that although they were made of local copper ore (Hunt Ortiz et al. 2012), their morphology may have been inspired by Levantine prototypes (Gernez 2011). The excavations carried out in the 1960s in order to protect the outermost part of the corridor with a concrete casemate, and in the 1990s when a new entrance for visitors was made, remain largely unpublished.

Three radiocarbon results on shells from within bioerosion perforations in two of the calcareous sandstone passage capstones have been previously reported (Cáceres Puro et al. 2014, Table 1; Table 2). As these are the remains of organisms which live in the intertidal zone, they must have burrowed into the rock before it was removed from the shore and used in the construction of the tomb. These results therefore provide *termini post quos* for the construction of the passage. Three new radiocarbon results have been obtained as part of this study (Table 2). The first two results come from a human bone (an adult metatarsal, CNA-1283) and a perforated shell bead (CNA-2504) from the 1991 excavation. The third one belongs to a human skull (CNA-1284) from the outer part of the corridor found in the 1963 excavation.

The interpretation of these dates is complicated by the clearly time-transgressive reservoir ages of the coastal waters off Andalucía (Monge Soares and Matos Martins 2010; Matos Martins and Monge Soares 2013; Monge Soares et al. 2016). The late fourth and third millennia cal BC is a particularly problematic time span as ocean circulation along the Andalucian coast changed rapidly (Matos Martins and Monge Soares 2013, Fig. 2). Since all the dated shells appear to date from a time when upwelling occurred along this stretch of coast, the Marine13 calibration curve (Reimer et al. 2013) and a mean ΔR correction of 180 ± 66 BP (Matos Martins and Monge Soares 2013, p. 1130) have been used. The two dates on shells from capstone 16 have been combined after calibration on the basis that both are likely to have lived very shortly before the rock was quarried, or the shells would not have survived (Cáceres Puro et al. 2014, p. 443). Both this combined date and that from the shell within capstone 6 are interpreted as *termini post quos* for both the construction of the monument, and for the phase of activity related to the use of the tomb represented by the other dated samples.

**Fig. 47:**
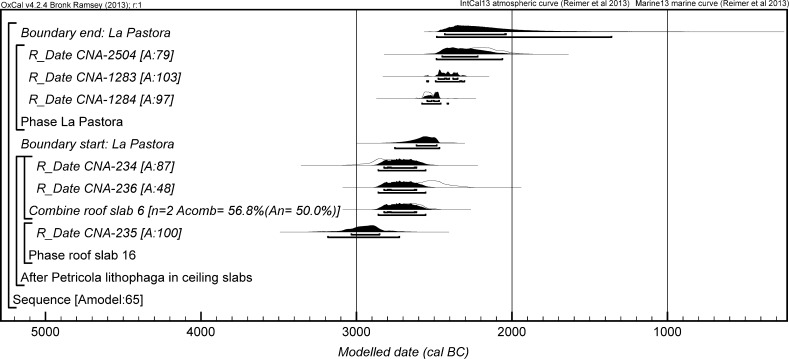
Chronological model for activity at La Pastora. The format is as described in Fig. 4. The large square ‘brackets’ down the left-hand side along with the OxCal keywords define the overall model exactly

**Fig. 48:**
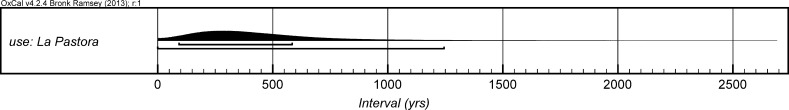
Probability distribution for the number of years over which activity at La Pastora took place. The distribution is derived from the model defined in Fig. 47

The model has good agreement (Amodel: 65), and estimates that the dated activity within the La Pastora tomb began in *2755*–*2465* *cal BC* (*95%* *probability*; *start: La Pastora*; Fig. 47), probably in *2615*–*2480* *cal BC* (*68%* *probability*). The activity ended in *2485*–*1360* *cal BC* (*95%* *probability*; *end: La Pastora*; Fig. 47), probably in *2435*–*2035* *cal BC* (*68%* *probability*). The duration of activity was *1*–*1245* *years* (*95%* *probability*; *use: La Pastora*; Fig. 48), probably *90*–*585* *years* (*68%* *probability*).

### Parcela Municipal

**Fig. 49:**
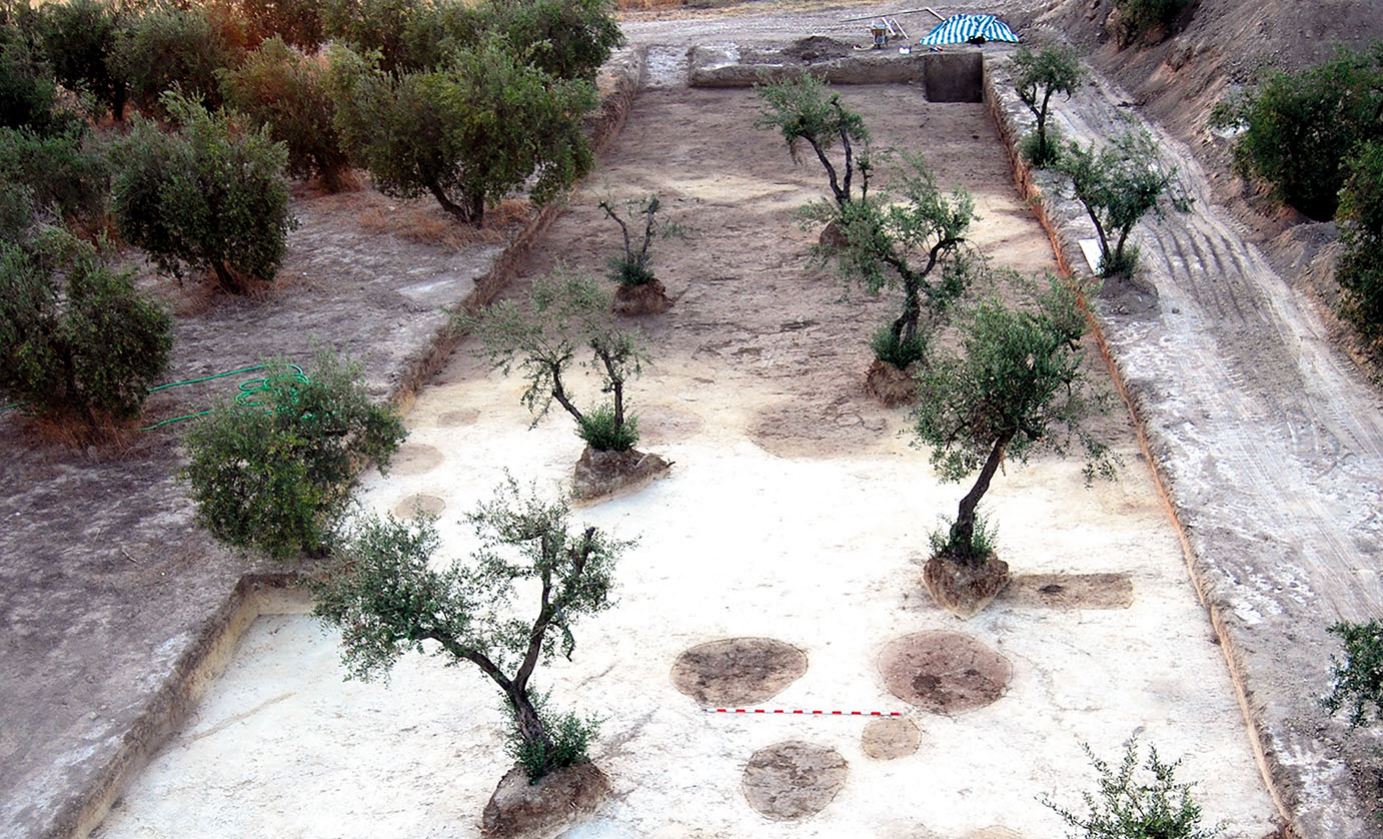
Overview of the 2011 excavation at Parcela Municipal, from the west, showing various circular pits in the foreground and the two parallel ditches in the background. Photo: Juan Manuel Vargas Jiménez

The excavations carried out in this sector in 2010 were meant as ground-testing of the results obtained in a previous magnetometer survey of the La Pastora area (Vargas Jiménez, Meyer and Ortega Gordillo 2012). Various negative features were found (Fig. 49). There were 23 circular pits, all about 1 m in diameter, with three types of infill: pits with very fine filling and scant material culture; pits with faunal remains (occasionally in anatomical connection) and well-preserved material culture; and a burial pit. A poly-lobulate feature (uc 54) was interpreted as a ‘hut-floor’. Two parallel ditches (186 and 206) were interpreted as corresponding to the outermost of Valencina’s hypothetical enclosures; Ditch186 has a maximum width of 7 m, while ditch 206 is 5.70 m at the widest, neither of them having been excavated to the bottom (a maximum depth of 2.50 m was reached in ditch 206).

**Fig. 50:**
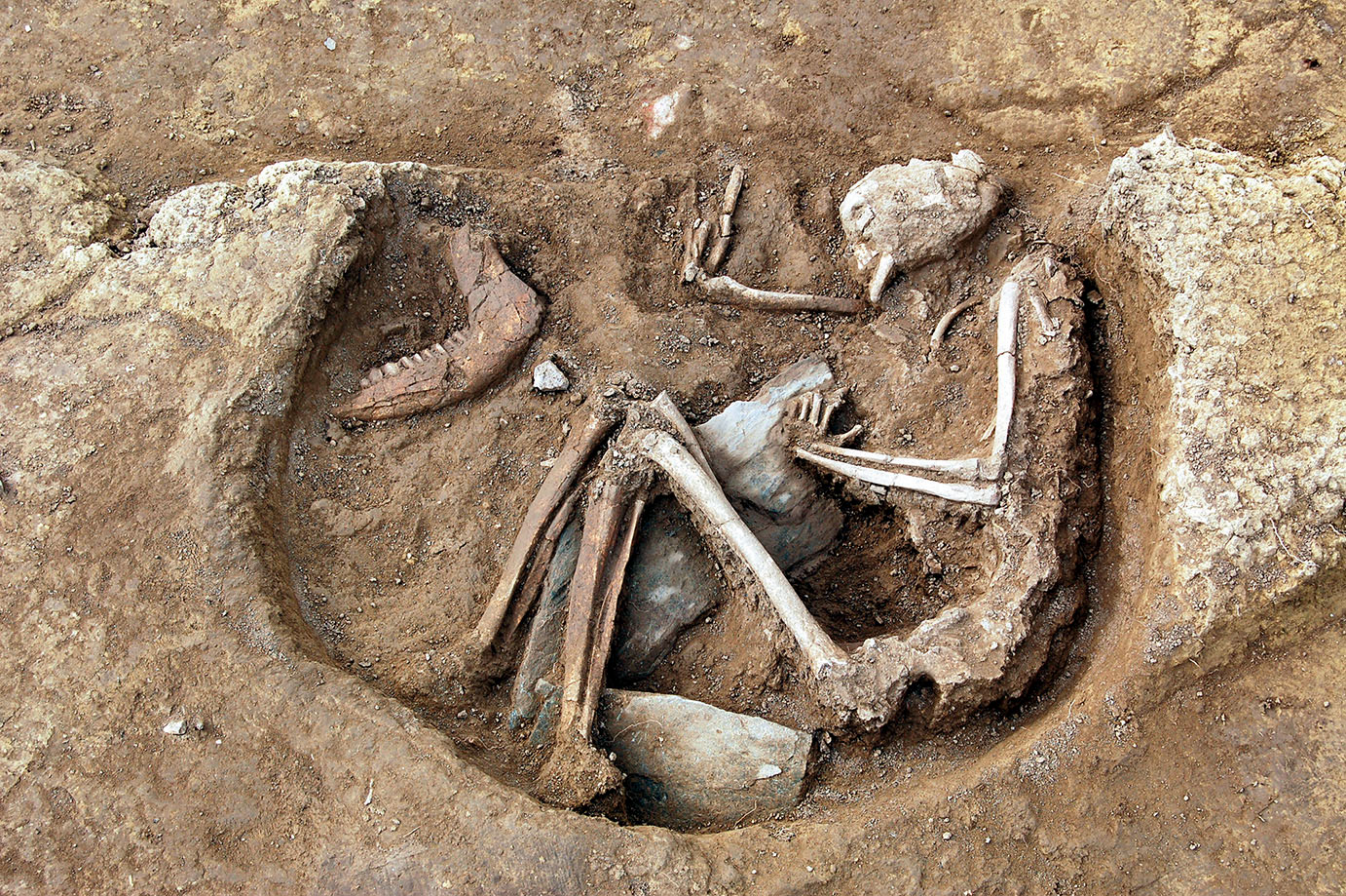
Detail of Parcela Municipal Structure 435. Photo: Juan Manuel Vargas Jiménez

There are seven radiocarbon measurements from samples of unidentified charcoal, animal bone and human bone (Table 2). Sample 105/412/29 (CNA-1098) is a fragment of charcoal from the lower layer of pit 105, which contained faunal remains in anatomical connection. Sample 91/86/29 (CNA-1101) was an unidentified fragment of charcoal taken from feature 91, also a circular pit that cuts the upper filling of feature 54. Both the samples of charcoal dated from feature 54 (CNA-1100 and -1497) were taken from the lowest stratigraphic deposit (54/248/50 and 54/243/44). Sample 186/187/50 (CNA-1496) is a fragment of animal bone recovered from the infill associated with a concentration of sun-dried mud (358) found on top of the filling of the more westerly ditch (N^o^ 186), and therefore marks a re-cutting of this ditch. Sample 206/401/32 (CNA-1099) was a fragment of charcoal taken from the upper part of the infill of ditch 206. Finally, sample 435/ROH437 (CNA-1499) corresponds to human bone from the individual inhumation found in Structure 435, in connection with half a cow mandible (Fig. 50).

**Fig. 51:**
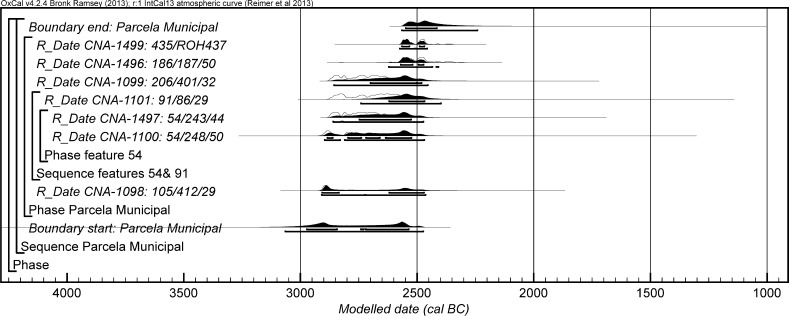
Chronological model for the activity at Parcela Municipal. The format is as described in Fig. 4. The large square ‘brackets’ down the left-hand side along with the OxCal keywords define the overall model exactly

**Fig. 52:**
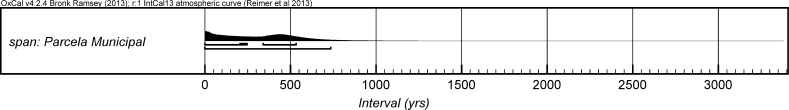
Probability distribution for the number of years over which activity at Parcela Municipal took place. The distribution is derived from the model defined in Fig. 51

The seven results have been put into a simple chronological model which assumes that they belong to a phase of related activity, and, to account for the possibility that the dated fragments of charcoal included some inbuilt age, we have employed the exponential charcoal outlier model proposed by Dee and Bronk Ramsey (2014) (Outlier_Model(“Charcoal”,Exp(1,-10,0),U(0,3),”t”); with a prior probability of 1 for each measurement on a charcoal sample). This model is shown in Fig. 51, and estimates that the dated activity with Parcela Municipal began in *3065*–*2470* *cal BC* (*95%* *probability*; *start: Parcela Municipal*; Fig. 51), probably in *2975*–*2535* *cal BC* (*68%* *probability*). The activity ended in *2570*–*2240* *cal BC* (*95%* *probability*; *end: Parcela Municipal*; Fig. 51), probably in *2550*–*2415* *cal BC* (*68%* *probability*). The duration of activity was *1*–*735* *years* (*95%* *probability*; *span: Parcela Municipal*; Fig. 52), probably *1*–*250* *years* (*38%* *probability*) or *340*–*535* *years* (*30%* *probability*). (A model which assumes that the dated fragments of unidentified charcoal at Parcela Municipal did not have large inbuilt age was constructed as a sensitivity analysis. This approach shifts the posterior distribution for the start of activity at Parcela Municipal earlier by more than 170 years, and shifts the posterior distribution for the end of that activity later by a decade or so. Again, model outputs are strongly affected by the character of the dated material.)

## A Sensitivity Analysis: The Possibility of Dietary Offsets

Diet-induced radiocarbon offsets can occur if a dated individual has taken up carbon from a reservoir not in equilibrium with the terrestrial biosphere (Lanting and van der Plicht 1998). If a subject consumed foods from a depleted source, such as marine fish or some freshwater fish, then the bone will take on some proportion of radiocarbon that is not in equilibrium with the atmosphere, making the radiocarbon age older than it would be if the individual had consumed a diet consisting of purely terrestrial resources. Assessing the proportion of the individual’s diet which is derived from such ^14^C-depleted reservoirs is consequently important for the calibration of the radiocarbon age of the skeleton, as calibration using a purely terrestrial calibration curve will produce anomalously early radiocarbon dates (cf. Ascough et al. 2007).

The most reliable method of checking for the presence of a reservoir offset in the human bone samples would be to date ‘perfect pairs’ of articulating herbivore and omnivore bone from the same closed context and to then compare the results. Unfortunately, no such samples have so far been retrieved from Valencina. For this reason, source-proportional dietary modelling was undertaken on the basis of carbon and nitrogen stable isotopic values, so that a personal calibration curve could be constructed for each dated human that would account for any potential reservoir effects arising from the diet of that individual.

Diet reconstruction for the dated humans from Valencina de la Concepción was undertaken using the Bayesian mixing model FRUITS v.2.0β (Food Reconstruction Using Isotopic Transferred Signals; Fernandes et al. 2014). A source-proportional dietary mixing model is constructed in FRUITS using the carbon and nitrogen stable isotopic values for each individual, and the isotopic averages and analytical uncertainties of likely food sources and diet-to-tissue isotopic offsets. The FRUITS model then produces estimates of the dietary proportion (and standard deviation) of each given food source for each consumer.

### Assumptions for the FRUITS Dietary Modelling

Stable isotope data (δ^13^C and δ^15^N) were produced by the Oxford Radiocarbon Accelerator Unit (OxA-) and the Scottish Universities Environmental Research Centre (SUERC-) for all samples of human or animal bone dated under the auspices of the ToTL project (Table 3). The methods used in these analyses and the reproducibility of these measurements are discussed above.

Possible food source types for the mixing model are based on the physical environment of the Valencina site (Fig. 2). Agriculture on the El Aljarafe plateau would have produced cereal crops, and faunal remains from the sites include a variety of domesticated herbivores. While no fish bones have been recovered from the site (which may be due either to issues of preservation or to archaeological collection methodologies), an assemblage of shellfish from the site, including scallops (Moreno Nuño 1995), suggests that aquatic foods may have been consumed. Additional protein food sources available at that time include freshwater fish from the River Guadalquivir and its marshes, and shellfish and marine fish from the Guadalete Estuary, which opens into the Gulf of Cadiz.

Mean isotopic values for four food groups (cereals, terrestrial protein, freshwater fish and marine fish) were used in modelling human diets in the Valencina population. The average isotopic values for cereals (δ^13^C = −22.81 ± 0.1‰; δ^15^N = 7.82 ± 0.15‰) include archaeobotanical barley and wheat from Bronze Age Terlinques, in southeast Spain (δ^13^C: Mora-González et al. 2016; δ^15^N: Mora-González pers. comm). The terrestrial herbivore values (δ^13^C = − 19.7 ± 0.2‰; δ^15^N = 6.4 ± 0.2‰) include sheep/goat, pig and cattle from the sites of Calle Mariana de Pineda s/n and Calle Trabajadores N^os^ 14–18 (Table 3), and from Montelirio and La Pastora (Fontanals-Coll et al. 2017, Table 2). As there are currently no data on archaeological remains of freshwater fish from the region, modern values from two Iberian ecological studies (Soto et al. 2016, Table 1; Martino et al. 2011, Fig. 2) served as a proxy. The δ^13^C values for modern freshwater fish have been offset by +0.85‰ (Böhm et al. 2002) for the Suess effect on δ^13^C, the anthropogenic atmospheric depletion of ^13^C since the Industrial Revolution (Suess 1958). This correction created working values for freshwater fish of δ^13^C = − 26.1 ± 1.2‰ and δ^15^N = + 16.4 ± 1.1‰. Marine food source isotopic values were derived from four species of marine fish from archaeological deposits in the Balearic Islands (δ^13^C = − 13.42 ± 0.2‰ and δ^15^N = + 9.36 ± 0.2‰; Garcia-Guixé et al. 2010, Table 1).

Isotopic fractionation during tissue building requires a diet-to-tissue offset for the FRUITS modelling. The offsets in this model were 4.8 ± 0.2‰ for δ^13^C and 5.5 ± 0.5‰ for δ^15^N (Fernandes et al. 2014). Lastly, the weight and concentration for each of the four diet sources in the model were set at 100%.

### Dietary Analysis of Human Remains

There are 65 humans with isotopic values from Copper Age Valencina whose dietary proportions of freshwater and marine fish have been modelled individually (Table 4). These people come from seven sectors: Calle Dinamarca N^os^ 3–5, Calle Mariana de Pineda s/n, Calle Trabajadores N^os^ 14–18, IES, La Huera, Montelirio tholos, and PP4-Montelirio. There are 28 dated individuals without stable isotopic analysis. For those from Calle Mariana de Pineda s/n, Montelirio tholos and PP4-Montelirio, we have taken an average of the FRUITS proportional diet estimates for each site (Table 5). For the remaining sectors with no stable isotopic analyses (Cerro de la Cabeza, El Algarrobillo, La Alcazaba, La Cima, La Gallega, La Pastora and Parcela Municipal), we have used the overall average of the proportional diet estimates for Valencina (Table 5).

**Table 4:** FRUITS proportional diet modelling results for individuals with measured isotopic ratios from the Valencina complex (see Table 3 for isotopic values and details of sampled skeletons)

Laboratory number	Cereals	Terrestrial protein	Freshwater fish	Marine fish
La Huera				
OxA-28234	52.4% ± 18.8%	16.5% ± 13.6%	26.2% ± 8.3%	4.9% ± 4.0%
SUERC-47677	78.6% ± 12.9%	18.9% ± 12.8%	1.3% ± 1.3%	1.2% ± 1.2%
SUERC-47678	85.3% ± 10.4%	11.6% ± 9.9%	1.7% ± 1.7%	1.3% ± 1.3%
SUERC-60397 & OxA-32263	78.1% ± 13.1%	19.6% ± 13.0%	1.1% ± 1.1%	1.2% ± 1.2%
OxA-28235 & SUERC-47679	72.5% ± 15.5%	25.0% ± 15.4%	1.2% ± 1.2%	1.3% ± 1.3%
OxA-28236–7	75.1% ± 14.2%	22.3% ± 14.1%	1.2% ± 1.2%	1.4% ± 1.3%
OxA-28238	81.4% ± 12.1%	15.6% ± 11.7%	1.6% ± 1.6%	1.4% ± 1.4%
OxA-30330	78.8% ± 13.2%	18.7% ± 13.1%	1.3% ± 1.2%	1.2% ± 1.2%
OxA-30331	71.9% ± 15.5%	24.9% ± 15.3%	1.6% ± 1.6%	1.7% ± 1.6%
OxA-30332 & SUERC-53937	78.4% ± 13.1%	19.5% ± 13.0%	1.0% ± 1.0%	1.1% ± 1.0%
OxA-30333 & SUERC-53942	80.7% ± 12.6%	17.1% ± 12.4%	1.1% ± 1.1%	1.1% ± 1.1%
OxA-30334	83.2% ± 10.8%	14.3% ± 10.7%	1.3% ± 1.3%	1.2% ± 1.2%
SUERC-47680 & OxA-28323	80.7% ± 13.8%	16.9% ± 13.5%	1.3% ± 1.3%	1.1% ± 1.1%
SUERC-47681	80.6% ± 12.7%	16.7% ± 12.5%	1.3% ± 1.3%	1.4% ± 1.3%
SUERC-53938	84.4% ± 10.4%	13.4% ± 10.3%	1.1% ± 1.1%	1.1% ± 1.0%
SUERC-53943	88.1% ± 8.5%	9.2% ± 8.2%	1.5% ± 1.4%	1.1% ± 1.1%
SUERC-53944	80.5% ± 12.7%	16.9% ± 12.7%	1.3% ± 1.3%	1.3% ± 1.2%
Calle Dinamarca N^os^ 3–5				
SUERC-47667 & OxA-32307	77.7% ± 15.0%	20.2% ± 15.0%	1.0% ± 1.0%	1.1% ± 1.1%
SUERC-47668 & OxA-X-2633-40	79.9% ± 12.6%	18.0% ± 12.5%	1.0% ± 1.0%	1.1% ± 1.1%
OxA-28239	76.4% ± 14.8%	20.8% ± 14.6%	1.4% ± 1.4%	1.4% ± 1.4%
OxA-28240	79.5% ± 12.5%	17.5% ± 12.2%	1.5% ± 1.5%	1.4% ± 1.4%
OxA-30335	73.7% ± 14.9%	22.6% ± 14.6%	1.9% ± 1.8%	1.9% ± 1.8%
OxA-30337 & SUERC-60399	76.0% ± 14.7%	22.0% ± 14.5%	1.0% ± 1.0%	1.0% ± 1.0%
OxA-30338	74.6% ± 13.8%	21.8% ± 13.7%	1.8% ± 1.7%	1.8% ± 1.7%
OxA-30339 & SUERC-53948	84.0% ± 11.4%	13.4% ± 11.3%	1.3% ± 1.3%	1.3% ± 1.2%
OxA-32306, SUERC-60398 & OxA-30336	70.8% ± 7.1%	26.6% ± 6.8%	1.4% ± 1.2%	1.2% ± 1.0%
SUERC-47669 & OxA-28241	79.9% ± 12.6%	18.0% ± 12.5%	1.0% ± 1.0%	1.1% ± 1.1%
SUERC-47670	83.3% ± 11.7%	14.7% ± 11.7%	1.0% ± 0.9%	1.0% ± 1.0%
SUERC-53945	82.2% ± 12.2%	15.4% ± 12.1%	1.2% ± 1.2%	1.1% ± 1.1%
SUERC-53946	88.3% ± 8.3%	9.3% ± 8.2%	1.3% ± 1.4%	1.1% ± 1.1%
SUERC-53947	80.2% ± 12.1%	17.5% ± 12.0%	1.1% ± 1.1%	1.2% ± 1.2%
I.E.S.				
OxA-28286	86.8% ± 9.6%	11.2% ± 9.6%	1.0% ± 1.0%	1.0% ± 0.9%
OxA-30381 & SUERC-53963	81.9% ± 11.8%	15.9% ± 11.6%	1.0% ± 1.0%	1.1% ± 1.1%
OxA-32308	90.2% ± 6.6%	7.0% ± 6.2%	1.7% ± 1.7%	1.1% ± 1.1%
OxA-32309	68.0% ± 18.5%	29.3% ± 18.4%	1.3% ± 1.2%	1.4% ± 1.4%
OxA-X-2586-22	85.9% ± 9.8%	11.3% ± 9.5%	1.6% ± 1.6%	1.3% ± 1.2%
SUERC-47676 & OxA-28287	88.0% ± 9.0%	9.9% ± 8.7%	1.2% ± 1.2%	1.0% ± 0.9%
SUERC-53962	75.6% ± 12.9%	22.6% ± 13.0%	0.9% ± 0.9%	0.9% ± 0.8%
SUERC-53964	89.2% ± 8.5%	8.8% ± 8.4%	1.1% ± 1.1%	0.9% ± 0.9%
PP4-Montelirio				
SUERC-60401	81.2% ± 11.7%	15.8% ± 11.6%	1.5% ± 1.4%	1.6% ± 1.6%
OxA-32370	74.8% ± 14.3%	22.7% ± 14.2%	1.2% ± 1.2%	1.3% ± 1.3%
OxA-32299	85.6% ± 8.8%	9.5% ± 7.8%	3.2% ± 2.9%	1.8% ± 1.7%
OxA-32300	80.8% ± 13.0%	16.4% ± 12.7%	1.5% ± 1.5%	1.3% ± 1.3%
Montelirio tholos				
OxA-32303	81.4% ± 12.1%	15.6% ± 11.7%	1.6% ± 1.6%	1.4% ± 1.4%
SUERC-47682	80.1% ± 13.3%	17.0% ± 13.0%	1.5% ± 1.5%	1.4% ± 1.4%
OxA-32301	80.1% ± 13.1%	17.5% ± 13.1%	1.2% ± 1.2%	1.3% ± 1.3%
OxA-28245, OxA-32304 & SUERC-60405	81.9% ± 12.4%	15.7% ± 12.1%	1.2% ± 1.3%	1.1% ± 1.1%
OxA-32302	71.9% ± 15.3%	24.2% ± 15.1%	2.1% ± 2.1%	1.9% ± 1.8%
OxA-30439	76.0% ± 14.0%	20.9% ± 13.9%	1.6% ± 1.5%	1.5% ± 1.5%
OxA-X-2535-32	74.2% ± 14.8%	22.9% ± 14.6%	1.4% ± 1.4%	1.5% ± 1.5%
OxA-30385	83.2% ± 10.2%	13.0% ± 9.7%	2.1% ± 2.1%	1.7% ± 1.6%
SUERC-47686	81.4% ± 12.1%	15.6% ± 11.7%	1.6% ± 1.6%	1.4% ± 1.4%
Calle Mariana de Pineda				
OxA-30340	68.7% ± 19.3%	29.3% ± 19.3%	1.0% ± 1.0%	1.1% ± 1.1%
OxA-32305	71.9% ± 15.5%	24.9% ± 15.3%	1.6% ± 1.6%	1.7% ± 1.6%
SUERC-60400	86.4% ± 9.6%	11.3% ± 9.5%	1.1% ± 1.1%	1.1% ± 1.1%
Calle Trabajadores N^os^ 14–18				
OxA-30341 & SUERC-53954	84.3% ± 12.6%	13.2% ± 11.2%	1.4% ± 1.3%	1.1% ± 1.1%
OxA-30342 & SUERC-60391	65.1% ± 15.3%	32.8% ± 15.3%	1.0% ± 1.0%	1.1% ± 1.1%
OxA-28242	88.5% ± 8.2%	9.6% ± 8.0%	1.0% ± 1.1%	0.9% ± 0.9%
OxA-28243	82.8% ± 11.6%	14.7% ± 11.4%	1.3% ± 1.3%	1.2% ± 1.1%
OxA-28244	91.5% ± 5.8%	5.5% ± 5.2%	2.1% ± 2.1%	1.0% ± 0.9%
SUERC-47671	85.2% ± 10.2%	12.1% ± 10.0%	1.4% ± 1.4%	1.2% ± 1.2%
SUERC-53953	76.2% ± 15.1%	21.8% ± 15.0%	1.0% ± 1.0%	1.1% ± 1.0%
OxA-30379, OxA-30400 & SUERC-60395	77.9% ± 13.2%	19.8% ± 13.0%	1.1% ± 1.2%	1.2% ± 1.2%
SUERC-53956	78.1% ± 14.3%	19.4% ± 14.2%	1.2% ± 1.2%	1.3% ± 1.2%
SUERC-53957	77.0% ± 14.2%	20.3% ± 14.0%	1.4% ± 1.4%	1.3% ± 1.3%

**Table 5:** FRUITS proportional diet modeling results for individuals without measured isotopic ratios from the Valencina complex (see Tables 2, 3 for isotopic values and details of sampled skeletons)

Site	Laboratory number	Cereals	Terrestrial protein	Freshwater fish	Marine fish
Calle Mariana de Pineda s/n*	Sac-2216	75.7% ± 14.8%	21.8% ± 14.7%	1.2% ± 1.2%	1.3% ± 1.3%
Montelirio tholos*	CNA-585–6 & Ua-40803	78.9% ± 13.0%	18.0% ± 12.8%	1.6% ± 1.6%	1.5% ± 1.4%
Montelirio tholos*	Ua-40801*				
Montelirio tholos*	Ua-40802*				
PP4-Montelirio*	CNA-1291	80.6% ± 11.9%	16.1% ± 11.6%	1.8% ± 1.7%	1.5% ± 1.5%
PP4-Montelirio*	CNA-1300				
PP4-Montelirio*	CNA-1301				
PP4-Montelirio*	CNA-1303				
Cerro de la Cabeza**	CNA-1277	79.5% ± 12.5%	17.4% ± 12.2%	1.7% ± 1.5%	1.3% ± 1.3%
Cerro de la Cabeza**	CNA-1278				
Cerro de la Cabeza**	CNA-1279				
El Algarrobillo**	CNA-1267				
El Algarrobillo**	CNA-1269				
El Algarrobillo**	CNA-1270				
El Algarrobillo**	CNA-1271				
El Algarrobillo**	CNA-1272				
El Algarrobillo**	CNA-1273				
El Algarrobillo**	CNA-1276				
La Alcazaba**	CNA-1260				
La Alcazaba**	CNA-1261				
La Alcazaba**	CNA-1262				
La Alcazaba**	CNA-1263				
La Cima**	CNA-1265				
La Cima**	CNA-1266				
La Gallega**	CNA-1264				
La Pastora**	CNA-1283				
La Pastora**	CNA-1284				
Parcela Municipal**	CNA-1499				

*Average derived from values for individuals with measured isotopes at given site, as shown in Table 4

**Average for other sites with no isotopic values derived from the overall average for the Valencina complex

Mean isotopic values for juveniles/sub-adults (15 years old or younger; n = 6) are −19.5 ± 0.2‰ for δ^13^C and +10.0 ± 0.3‰ for δ^15^N. Mean isotopic values for all unsexed and sexed adults of the Valencina population (17 years and older; n = 59) are −19.2 ± 0.2‰ for δ^13^C and +9.3 ± 0.3‰ for δ^15^N.

While there are no significant differences in δ^13^C and δ^15^N between the juvenile and adult age cohorts, the minimum and maximum isotopic values over the entire sample population do differ by 7.5‰ in δ^15^N, indicating a notable variation in the type and amount of animal protein in the diet, as illustrated by the minimum and maximum proportional values of terrestrial animal and fish (Table 4).

The diets of adults, juveniles and sub-adults (including individuals of unknown age and sex, which on the basis of scanning during sampling for radiocarbon dating probably all fall into these categories) generally indicate a substantial reliance on crops, followed by varying amounts of terrestrial protein from herbivores. The overall population has a mean dietary proportion of 79.5 ± 12.5% cereals (minimum 52.4 ± 5.8%, maximum 91.5 ± 19.3%). Terrestrial herbivores make up an average of 17.4 ± 12.2% of diets, but the variation in the diet proportion is between a low of 5.5 ± 5.2% and a high of 32.8 ± 19.3%. The large associated errors on the estimates probably arise from the small difference (1.4‰) between the baseline δ^15^N values of cereals and the baseline δ^15^N values for terrestrial herbivores in the FRUITS modelling.

Both freshwater and marine fish appear to have been an insignificant part of the diet, with the human population averages for freshwater and marine fish being 1.7 ± 1.5% and 1.3 ± 1.3%, respectively. The exceptions are a 12–15-year-old female from La Huera (OxA-28234; δ^13^C = − 18.5 ± 0.2‰, δ^15^N = 15.1 ± 0.3‰) with an estimated proportion of 26.2 ± 8.3% freshwater fish and 4.9 ± 4.0% marine fish. A second individual with enriched δ^15^N, a young adult from PP4-Montelirio (OxA-32299; δ^13^C = −19.1 ± 0.2‰, δ^15^N = 11.7 ± 0.3‰) had an estimated proportion of 3.2 ± 2.9% freshwater fish and 1.8 ± 1.7% marine fish.

The FRUITS estimated diet proportions for Valencina as a whole describe diets that are similar to those reported for the Montelirio tholos by Fontanals-Coll et al. (2016), where the diets’ protein component was largely based on meat and C_3_ plant protein from cereals and pulses. FRUITS modelling also determined that, except in two specific instances, freshwater or marine fish in Valencina diets was probably negligible.

### Mixed-Source Radiocarbon Calibration and Chronological Modelling

The construction of personal calibration curves to account for the proportion of non-terrestrial resources consumed by each dated individual is particularly complex at Valencina. We have no data for assessing the potential radiocarbon reservoirs of either the waters of the Guadalquivir or of the marshland at its confluence with the sea. The reservoir ages of the coastal waters off Andalucía are relatively well understood, but are clearly time-transgressive (Monge Soares and Matos Martins 2010; Matos Martins and Monge Soares 2013; Monge Soares et al. 2016). In these circumstances, we have combined the estimated proportions of marine and freshwater resources estimated by the FRUITS model (which we take to cover any resources from the lagoon), and used a generic reservoir age of 600 ± 100 BP. (This reservoir offset is likely to encompass both the marine reservoir at the time the tholos was in use—when the mean ΔR correction along the Andalucian coast was 180 ± 66 BP [Matos Martins and Monge Soares 2013, p. 1130]—and a plausible average offset of freshwater resources [cf. Keaveney and Reimer 2012; Bonsall et al. 2015].) We use this reservoir, offset from the atmospheric calibration dataset (Reimer et al. 2013), and the Mix_Curves function of OxCal v4.2 (Bronk Ramsey 2001, amended following Jones and Nicholls 2001). For each dated individual, we have constructed a personal calibration curve, which incorporates the reservoir in the proportion suggested by the combined dietary estimate for freshwater and marine resources provided by the FRUITS model in that particular individual (Tables 4 and 5). So, for example, OxA-30330 (2229, Individual 20 from La Huera) has been calibrated using a calibration curve including a component of 2.5 ± 2.4% non-terrestrial resources (note that the proportion of any curve is constrained to be 0–100%). The remainder of diet sources will be in equilibrium with the contemporary atmosphere and have been calibrated using IntCal13 (Reimer et al. 2013).

**Fig. 53:**
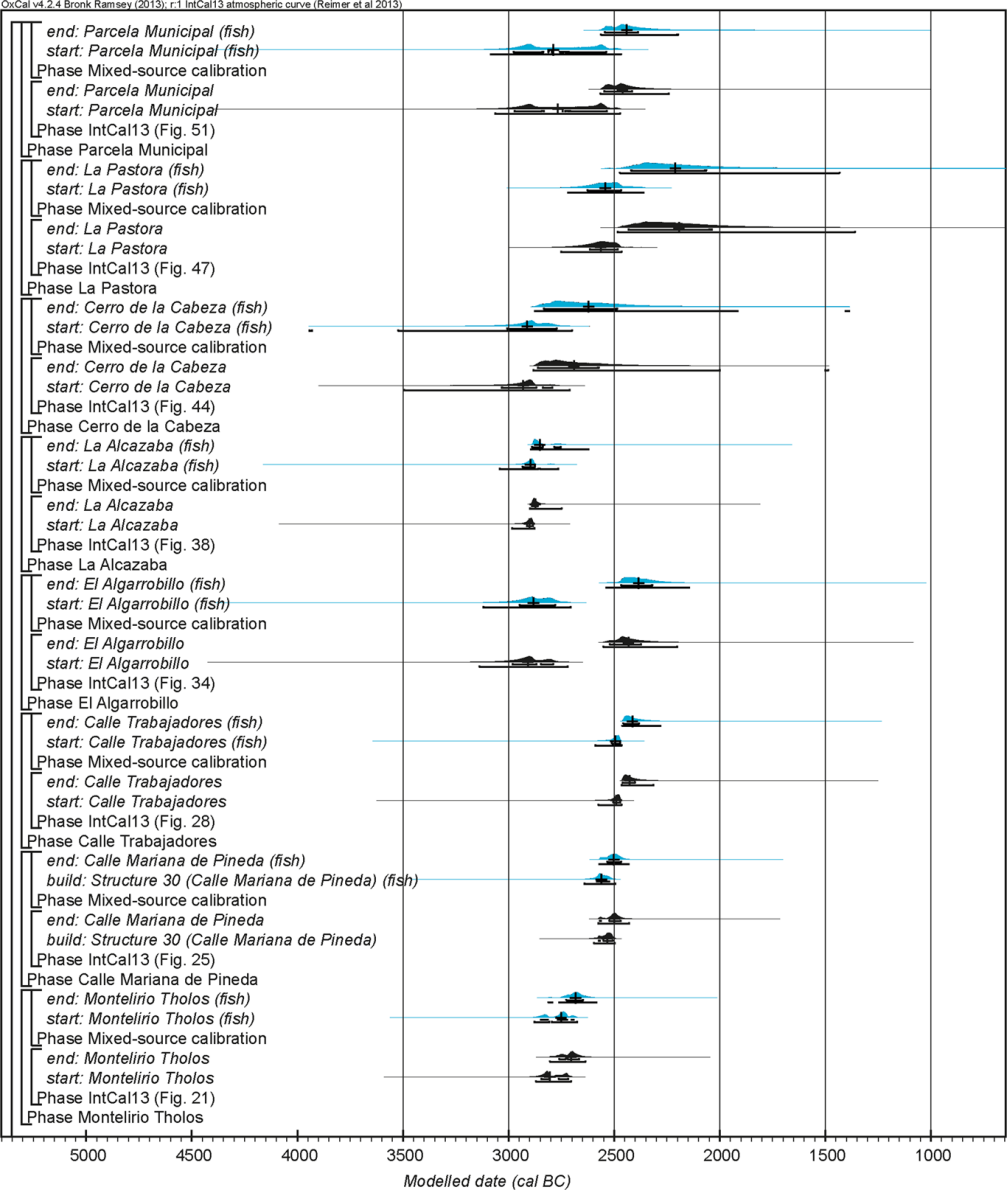

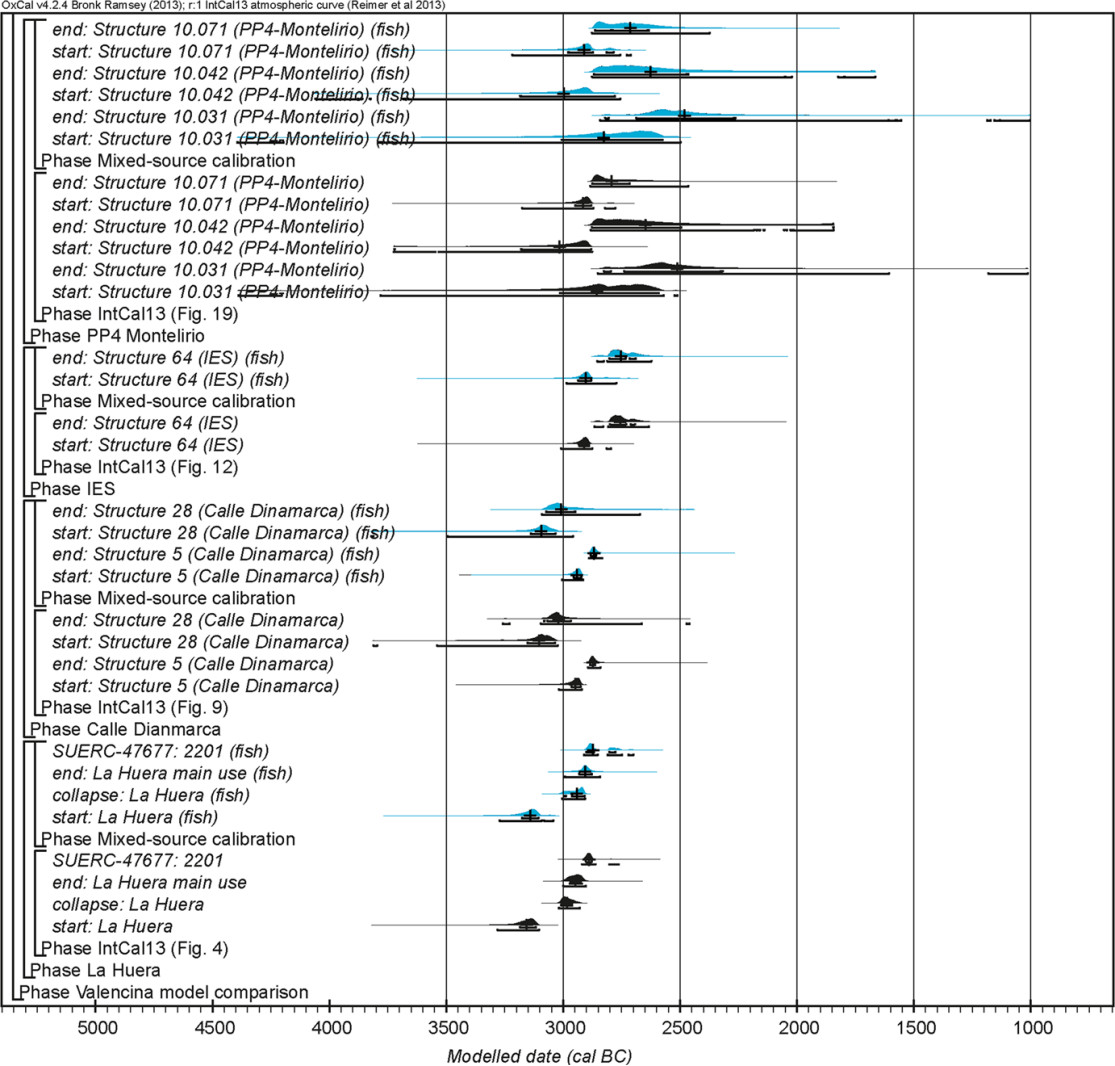
Key parameters from the chronological models from the Valencina complex which include dates on human bone, calculated using fully terrestrial calibration (IntCal13) (in black) and mixed-source calibration to allow for potential dietary reservoir effects (in blue; see text and Tables 4 and 5), derived from the models defined in Figs. 4, 9, 12, 19, 21, 25, 28, 34, 38, 44, 47 and 51 (Color figure online)

We have recalculated the preferred model for each site that has measurements on samples of human bone using the mixed-source calibration derived from the dietary modelling. In all cases the radiocarbon dates that have been calculated making allowance for potential dietary reservoir effects have good overall agreement with the prior information included in the models (Amodel > 60). The posterior distributions of key parameters from each of these models, calculated using both the fully terrestrial and mixed-source calibrations, are shown in Fig. 53 (and the Highest Posterior Density intervals are given in Table 6).

**Table 6:** Highest Posterior Density intervals for key parameters from the preferred chronological models from the Valencina complex, calculated using (a) fully terrestrial calibration (IntCal13) and (b) mixed-source calibration to allow for potential dietary reservoir effects (see text and Tables 4, 5)

Parameter	Terrestrial calibration		Mixed-source calibration		Difference between medians
	*Highest Posterior Density interval*		*Highest Posterior Density interval*		
	*95% probability*	*68% probability*	*95% probability*	*68% probability*	
La Huera (cf. Fig. 4)					
start: La Huera	*3285–3100 cal BC*	*3190–3115 cal BC*	*3275–3090 cal BC (91%)* or *3085–3040 cal BC (4%)*	*3180–3105 cal BC*	14
*collapse: La Huera*	*3020–2925 cal BC*	*3010–2960 cal BC*	*3010–2905 cal BC*	*3000–2990 cal BC (4%)* or *2965–2910 cal BC (64%)*	42
*end: La Huera main use*	*3000–2900 cal BC*	*2975–2920 cal BC*	*2995–2840 cal BC*	*2935–2875 cal BC*	50
*SUERC-47677: 2201*	*2920–2860 cal BC (88%)* or* 2805–2760 cal BC (7%)*	*2905–2875 cal BC*	*2915–2850 cal BC (58%)* or* 2815–2745 cal BC (33%)* or* 2725–2700 cal BC (4%)*	*2905–2865 cal BC (49%)* or* 2805–2775 cal BC (19%)*	13
Calle Dinamarca N^os^ 3–5 (cf. Fig. 9)					
*start: Structure 5 (Calle Dinamarca)*	*3020–2920 cal BC*	*2970–2925 cal BC*	*3010–2915 cal BC*	*2960–2920 cal BC*	7
*end: Structure 5 (Calle Dinamarca)*	*2900–2840 cal BC*	*2890–2860 cal BC*	*2895–2830 cal BC*	*2885–2855 cal BC*	4
*start: Structure 28 (Calle Dinamarca)*	*3545–3020 cal BC*	*3155–3030 cal BC*	*3500–2955 cal BC*	*3140–3030 cal BC*	9
*end: Structure 28 (Calle Dinamarca)*	*3260–3230 cal BC (1%)* or* 3100–2660 cal BC (94%)*	*3070–2965 cal BC*	*3095–2675 cal BC*	*3075–2950 cal BC*	11
IES (cf. Fig. 12)					
*start: Structure 64 (IES)*	*3010–2875 cal BC (94%)* or* 2820–2795 cal BC (1%)*	*2935–2890 cal BC*	*2990–2775 cal BC*	*2940–2880 cal BC*	8
*end: Structure 64 (IES)*	*2870–2830 cal BC (6%)* or* 2810–2630 cal BC (89%)*	*2805–2730 cal BC (59%)* or* 2715–2690 cal BC (9%)*	*2860–2825 cal BC (4%)* or* 2815–2620 cal BC (91%)*	*2805–2730 cal BC (55%)* or* 2720–2690 cal BC (13%*)	4
PP4-Montelirio (cf. Fig. 19)					
*start: Structure 10.031(PP4-Montelirio)*	*4390–4190 cal BC (3%)* or* 3795–2570 cal BC (92%)*	*3015–2590 cal BC*	*4395–4195 cal BC (3%)* or* 3785–2495 cal BC (92%)*	*3005–2575 cal BC*	30
*end: Structure 10.031(PP4-Montelirio)*	*2855–1580 cal BC (92%)* or* 1195–1010 cal BC (3%)*	*2830–2790 cal BC (3%)* or* 2745–2320 cal BC (65%)*	*2845–1560 cal BC (92%)* or* 1190–1000 cal BC (3%)*	*2825–2805 cal BC (1%)* or* 2690–2260 cal BC (67%)*	31
*start: Structure 10.042 (PP4-Montelirio)*	*3725–3555 cal BC (9%)* or* 3515–2875 cal BC (86%)*	*3185–2880 cal BC*	*4065–3850 cal BC (6%)* or* 3685–2755 cal BC (89%)*	*3185–2775 cal BC*	17
*end: Structure 10.042 (PP4-Montelirio)*	*2885–2155 cal BC (86%)* or* 2040–1840 cal BC (9%)*	*2880–2490 cal BC*	*2880–2010 cal BC (90%)* or* 1825–1665 cal BC (5%)*	*2870–2465 cal BC*	23
*start: Structure 10.071 (PP4-Montelirio)*	*3175–2870 cal BC (93%)* or* 2825–2775 cal BC (2%)*	*2950–2880 cal BC*	*3220–2755 cal BC*	*2980–2870 cal BC (60%)* or* 2820–2785 cal BC (8%)*	5
*end: Structure 10.071 (PP4-Montelirio)*	*2885–2465 cal BC*	*2880–2715 cal BC*	*2880–2375 cal BC*	*2865–2640 cal BC*	80
Montelirio tholos (cf. Fig. 21)					
*start: Montelirio Tholos*	*2875–2700 cal BC*	*2850–2805 cal BC (38%)* or* 2765–2715 cal BC (30%)*	*2880–2805 cal BC (28%)* or* 2800–2675 cal BC (67%)*	*2850–2815 cal BC (19%)* or* 2770–2720 cal BC (45%)* or* 2705–2690 cal BC (4%)*	53
*end: Montelirio Tholos*	*2805–2635 cal BC*	*2765–2730 cal BC (18%)* or* 2725–2665 cal BC (50%)*	*2815–2790 cal BC (2%)* or* 2765–2585 cal BC (93%)*	*2730–2645 cal BC*	22
Calle Mariana de Pineda s/n (cf. Fig. 25)					
*build: Structure 30 (Calle Mariana de Pineda)*	*2600–2495 cal BC*	*2575–2565 cal BC (3%)* or* 2555–2505 cal BC (65%)*	*2585–2490 cal BC*	*2570–2515 cal BC*	− 5
*end: Calle Mariana de Pineda*	*2580–2430 cal BC*	*2570–2560 cal BC (2%)* or* 2525–2470 cal BC (66%)*	*2575–2430 cal BC*	*2535–2465 cal BC*	− 2
Calle Trabajadores N^os^ 14–18 (cf. Fig. 28)					
*start: Calle Trabajadores*	*2580–2465 cal BC*	*2505–2470 cal BC*	*2595–2465 cal BC*	*2515–2470 cal BC*	− 4
*end: Calle Trabajadores*	*2470–2310 cal BC*	*2465–2400 cal BC*	*2465–2280 cal BC*	*2460–2380 cal BC*	13
El Algarrobillo (cf. Fig. 34)					
*start: El Algarrobillo*	*3140–2720 cal BC*	*2985–2865 cal BC (51%)* or* 2845–2785 cal BC (17%)*	*3125–2705 cal BC*	*2955–2780 cal BC*	28
*end: El Algarrobillo*	*2555–2200 cal BC*	*2525–2370 cal BC*	*2540–2140 cal BC*	*2470–2320 cal BC*	49
La Alcazaba (cf. Fig. 38)					
*start: La Alcazaba*	*2985–2875 cal BC*	*2915–2885 cal BC*	*3050–2860 cal BC (84%)* or* 2850–2760 cal BC (11%)*	*2935–2875 cal BC*	5
*end: La Alcazaba*	*2900–2750 cal BC*	*2895–2860 cal BC*	*2900–2625 cal BC*	*2890–2835 cal BC (56%)* or* 2790–2750 cal BC (12%)*	21
Cerro de la Cabeza (cf. Fig. 44)					
*start: Cerro de la Cabeza*	*3495–2710 cal BC*	*3035–2865 cal BC (61%)* or* 2840–2790 cal BC (7%)*	*3530–2700 cal BC*	*3010–2770 cal BC*	18
*end: Cerro de la Cabeza*	*2885–2000 cal BC*	*2865–2575 cal BC*	*2880–1910 cal BC*	*2835–2490 cal BC*	69
La Pastora (cf. Fig. 47)					
*start: La Pastora*	*2755–2465 cal BC*	*2615–2480 cal BC*	*2720–2360 cal BC*	*2630–2465 cal BC*	22
*end: La Pastora*	*2485–1360 cal BC*	*2435–2035 cal BC*	*2475–1415 cal BC*	*2425–2065 cal BC*	− 17
Parcela Municipal (cf. Fig. 51)					
*start: Parcela Municipal*	*3065–2470 cal BC*	*2975–2535 cal BC*	*3090–2465 cal BC*	*2980–2830 cal BC (32%)* or* 2755–2535 cal BC (36%)*	22
*end: Parcela Municipal*	*2570–2240 cal BC*	*2550–2415 cal BC*	*2565–2195 cal BC*	*2550–2385 cal BC*	20

In almost all cases the posterior distribution produced by the mixed-source models is compatible with those produced by the same model calculated using fully terrestrial calibration data. The differences between the medians of comparable parameters are given in Table 6. Most posterior distributions from the mixed-source models are slightly later than those from the fully terrestrial models, although this difference amounts to less than 25 years in over 70% of cases. In some of the other cases the medians of imprecise distributions shift more substantially (e.g. *end: Structure 10.071 (PP4*-*Montelirio)*; Fig. 53). This arises from the greater uncertainties introduced into the model by the estimates of dietary proportions, which mean that models that include insufficient data to adequately assess the statistical scatter on a group of related measurements are even less adequately constrained by the mixed-source modelling. In a few other cases (e.g. *collapse: La Huera; start: Montelirio Tholos*; Fig. 53), the mixed-source calibration shifts the balance of probabilities within a distribution, suggesting that a date about half a century later is more probable using the mixed-source calibration.

Given the small proportions of non-terrestrial dietary resources suggested by the FRUITS modelling, the uncertainties on those estimates, and our currently limited information about the stable isotopic baseline of food resources in this region and the local reservoirs in both the Guadalquivir and the marshland at its confluence with the sea, at present the modelling using fully terrestrial calibration probably provides a more robust estimate of the chronology of Valencina. This analysis demonstrates, however, that in most cases dietary reservoir effects are unlikely to shift this chronology later by more than a few decades, although for a few specific cases more substantial shifts of up to half a century may be possible.

## Discussion

### New Approaches to Old Themes: The Temporality and Character of Valencina de la Concepción

#### The Timing and Range of Activity

**Fig. 54:**
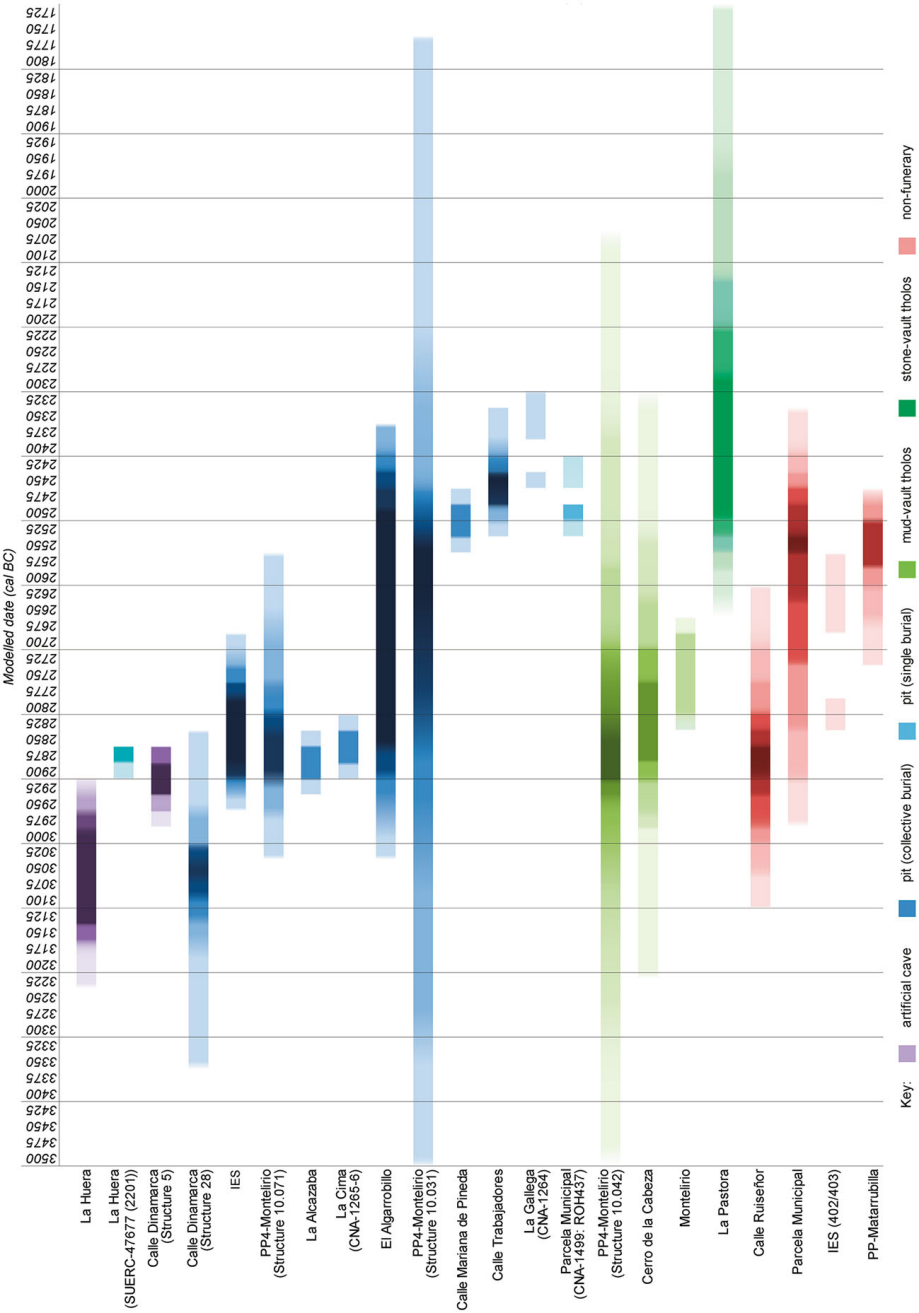
Schematic diagram showing the periods of use of the different sectors and structures at Valencina de la Concepción

It is striking that the two oldest of the 17 radiocarbon dated sectors in Valencina—La Huera and Calle Dinamarca N^os^ 3–5—are funerary deposits (Fig. 54; Supplementary Table S1). This observation seems of significance in determining the role of burial practices in the foundation of the site and its character as a whole. This earliest activity at Valencina probably dates to the last two centuries of the fourth millennium cal BC and does not seem to express itself in the form of structures devoted to dwelling, production or storage, but in burial deposits. Evidence also points to the use of La Huera and Calle Dinamarca N^os^ 3–5 as persistent places of burial for over a century—a more prolonged use than apparent for many other sectors of the site. It is, of course, possible that early non-funerary contexts await discovery, but on current evidence, the complex appears to have originated as a place for recurring funerary activity rather than as a permanent settlement.

**Fig. 55:**
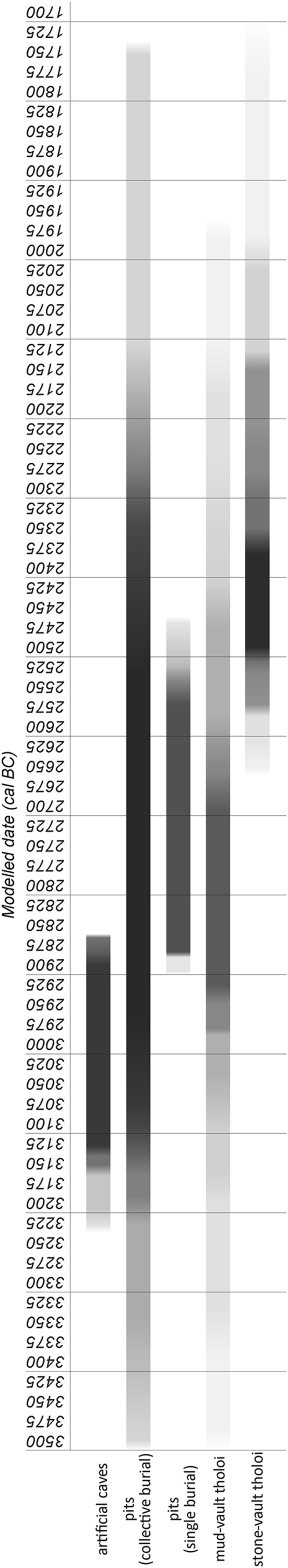
Schematic diagram showing the periods of use of different funerary practices at Valencina de la Concepción

Figure 54 is a schematic summary diagram showing the probability that the different dated elements of Valencina were in use in any given 25-year period (the darker the shading the more probable that an element of the site was in use at that time). Figure 55 then illustrates the currency of different forms of funerary practice at the site. This is based on the first dated event and last dated event for each type of funerary practice from the sectors shown on Fig. 54 (and so incorporates large uncertainties from, for example, the imprecisely dated features in the PP4-Montelirio sector). Nonetheless, it is clear that the first burials at the site probably occurred towards the end of the 32nd century cal BC, and that this took the form of collective burial either in pits or in artificial caves (Table 7). The use of artificial caves had ceased by the middle of the 29th century cal BC, around the time when the first single inhumations in pits are recorded on the site (since we have radiocarbon dates from only two such burials, this finding must be treated with considerable caution). The first tholos tombs were probably constructed at the very end of the 30th century cal BC or during the 29th century cal BC. These appear to have been constructed with vaults of sun-dried mud, an architecture that may have been replaced by stone corbelling during the 26th century cal BC. It is not clear whether these architectural methods of roofing were in overlapping use, or whether stone corbelling marked the ‘reinvention’ of an earlier tradition. Collective burial in pits and the making of a stone-vault at La Pastora continued until the end of funerary activity on the site in the 24th or earlier 23rd century cal BC.

**Table 7:** Probabilities of the order of the first and last dated occurrences of different funerary practices at Valencina de la Concepción

	*first: artificial cave*	*last: artificial cave*	*first: collective burial*	*last: collective burial*	*first: single burial*	*last: single burial*	*first: mud-vault tholos*	*last: mud-vault tholos*	*first: stone-vault tholos*	*last: stone-vault tholos*
*first: artificial cave*	–	100	50	100	100	100	60	100	100	100
*last: artificial cave*		–	0	100	18	100	0	100	100	100
*first: collective burial*			–	100	100	100	65	100	100	100
*last: collective burial*				–	0	0	0	25	0	65
*first: single burial*					–	100	2	100	100	100
*last: single burial*						–	0	49	26	100
*first: mud-vault tholos*							–	100	100	100
*last: mud-vault tholos*								–	41	80
*first: stone-vault tholos*									–	100
*last: stone-vault tholos*										–

The table gives the probability that the parameter down the left-hand column is earlier than the parameter along the top row. For example, the probability that *first: artificial cave* is earlier than *first: mud-vault tholos* is *60%*

Burial practices seem to define not only the initiation of the site, but also its final stages. This is suggested by Structure 1 of Calle Trabajadores N^os^ 14–18, the most recent of the 16 sectors dated as part of this investigation (Fig. 54; Supplementary Table S1). As described above, Calle Trabajadores N^os^ 14–18 Structure 1 is a negative feature, circular in plan and without stone elements, of approximately 1.8 m maximum diameter and a depth of 0.3 m. This structure yielded a deposit of nine human skulls and some articulated limbs in connection with the articulated leg of a pig. On the one hand, the modelled estimates are compatible with a single burial event (Fig. 28); in addition an ongoing bioarchaeological study has found numerous defleshing marks on the human bones (Herrero Corral 2015). The fact that Valencina’s largest collection of Bell Beaker pottery was also found in this sector (Inacio et al. 2017) further underlines the ritual character that these later deposits seem to have, an issue that is further discussed below.

Given the seeming ubiquity of mortuary deposits in a wide range of contexts, from megalithic tombs to simple pits, across all the sectors detailed in this paper, and from the very beginning until the very end of its occupation, it is hard to see the Valencina site just as a settlement. Nor is it easy to maintain the previous simple distinction between a ‘necropolis’ and a ‘domestic’ zone, even though it does appear that many of the megalithic tombs and related structures are concentrated in the eastern to southeastern part, which suggests that there may have been differentiated areas across the site. On the other hand, the details of features given sector by sector in this paper (and see also García Sanjuán, Vargas Jiménez et al. 2013b) make it clear that Valencina was not just a place for treatment or disposal of the dead. So far, no obvious residential buildings have been found, but there are many pits (as well as negative features of other types), containing the residues of a very wide range of activities other than treatment of the dead, and connected to domestic and productive activities. There are also the still poorly-understood ditches to be taken into account. While there are a host of questions to be resolved by future research, the most plausible working hypothesis with the evidence at hand is that Valencina was a place of major assembly, bringing people together, possibly from a wide area, for intense social gatherings and commemoration of the dead (and it is important to note that because of the sampling strategy followed within the ToTL project, human bone samples have been favoured, giving some pre-eminence to burial contexts). There is no need to exclude prolonged residence by some people or groups, though that does not seem to catch the character of the place as a whole. The now more differentiated chronology raises the possibility of going beyond a single, blanket statement about the nature of Valencina, and that is what we will now further explore.

#### The Intensity and Duration of Activity

**Fig. 56:**
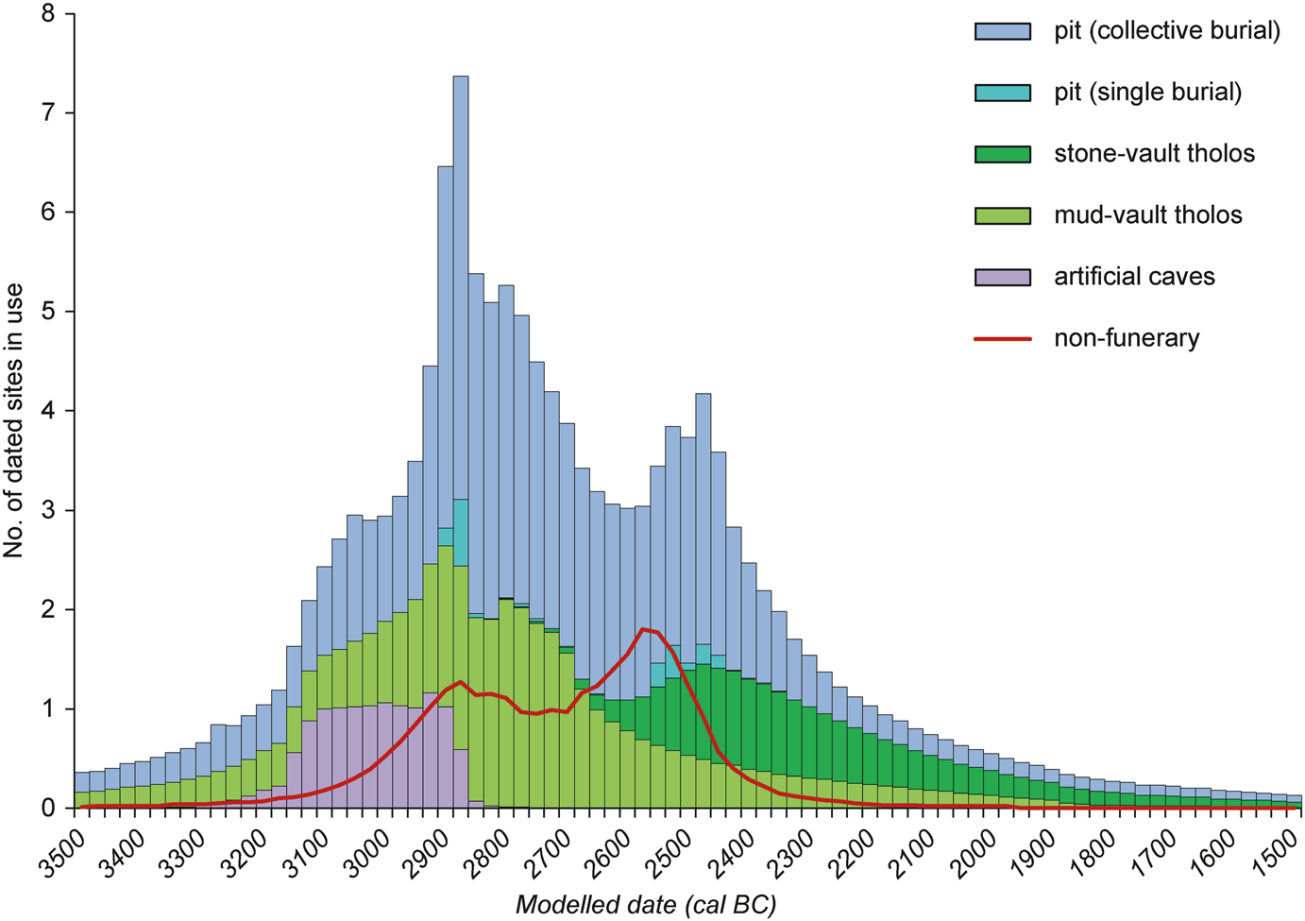
Number of dated funerary structures and non-funerary site occupied per 25-year period at Valencina de la Concepción, calculated from the normalised probability distributions for the use of each sector illustrated in Fig. 55

The dynamic character of activity at Valencina during the earlier part of the third millennium cal BC may be reflected in Fig. 56. This graph illustrates the intensity of different funerary practices and non-funerary activity on the site over its history, by adding the probability that each element of the site shown in Fig. 54 was in use in each 25-year period. So, for example, in the 25 years after 2900 cal BC, probably one of the dated artificial caves, three or four of the dated pits containing collective burials, one or two of the dated mud-vault tholoi, and a non-burial structure, were in use. Assuming that the structures that have been dated at Valencina are a representative sample of what was once there, this graph should reflect the intensity of activity on the site over time. Having started in the 32nd century cal BC, funerary activity seems to have peaked in the 29th century cal BC, persisting until at least the 24th century cal BC. Burial may have increased in intensity again on the site in the generations around 2500 cal BC, perhaps at the time when the stone-vaulted La Pastora tholos was constructed. The pattern of non-funerary activity is much less robust as we only have radiocarbon dates from four sectors (Fig. 54), but it is possible that this reflects a similar pattern to the burial structures.

A further conclusion of our study is that the investigated contexts exhibit usage patterns with quite different temporalities. Both the early artificial caves at La Huera and Calle Dinamarca N^os^ 3–5 display long spans suggesting repeated use by several generations (Figs. 5, 10). Of the nine collective burials in pits where more than one individual has been dated, six have produced statistically consistent groups of radiocarbon measurements at 2σ (Calle Dinamarca N^os^ 3–5 (Structure 28) (T′ = 2.8; T′ (5%) = 6.0; ν = 2), La Alcazaba (T′ = 3.3; T′ (5%) = 7.8; ν = 3), La Cima (T′ = 0.6; T′ (5%) = 3.8; ν = 1), PP4-Montelirio (Structure 10.031) (T′ = 0.0; T′ (5%) = 3.8; ν = 1), Calle Mariana de Pineda s/n (T′ = 2.8; T′ (5%) = 6.0; ν = 2), and Calle Trabajadores N^os^ 14–18 (T′ = 24.2; T′ (5%) = 27.7; ν = 17)); and one a group of measurements that are statistically consistent at 3σ, but not at 2σ (PP4-Montelirio (Structure 10.071) (T′ = 8.4; T′ (1%) = 11.3; ν = 3)). In each case, weighted means have been taken on replicate measurements on the same individual before calculation of the test statistic, and measurements identified as anomalous during the modelling process have been excluded. These results would be compatible with interpreting each act of collective burial as a single episode. Structure 64 in the IES sector is the most convincing exception to this *courte durée*, as this pit was probably a place of repeated burial for over a century (Fig. 13). As described above, on the basis of current evidence, the interpretation of Structure 1 at El Algarrobillo is vexed. If the dated samples are interpreted as coming from a continuous period of burial, then this clearly endured for a number of centuries (Fig. 35). But if we assume that the dated samples fall in two phases, indicated by the lower and upper levels in the pit, then burial at El Algarrobillo could have been concentrated in two episodes of collective burial, separated by a gap of several generations; the lower group of measurements shown in Fig. 36 are statistically consistent at 3σ; and the upper group are statistically consistent at 2σ (lower group: T′ = 8.3, T′ (1%) = 11.3; ν = 3; upper group: T′ = 3.4, T′ (5%) = 6.0; ν = 2). This scenario raises the question of whether this burial feature was marked externally, or whether it was remembered.

Estimating the duration of use of durable monuments that, once constructed, become points of reference and remembrance in the landscape is hazardous. In the absence of detailed stratigraphic information dividing primary use from subsequent activity (see, for example, the West Kennet long barrow in southern Britain; Bayliss, Whittle and Wysocki 2007b) or large suites of radiocarbon dates whose clustering may indicate different episodes of activity (as we have posited at El Algarrobillo), it is difficult to distinguish the duration of activity by the monument makers from later reuse of the site. For the tholoi considered here, we have best evidence about the duration of burial within Montelirio. This is discussed in detail above, but the initial period of burial in this tomb was clearly of restricted duration—confined to perhaps a few decades in the later 29th or 28th century cal BC (Fig. 22), or even to a single grand funeral (Fig. 23). The radiocarbon measurements on the dated human individuals from Structure 10.042 at PP4-Montelirio are statistically consistent at 3σ, but not at 2σ (PP4-Montelirio (Structure 10.042): T′ = 6.3, T′ (1%) = 6.6; ν = 1), and are also compatible with a relatively short period of use for these mud-vaulted tholoi of a few generations at most (Fig. 54). But the radiocarbon results on the dated human bones from Cerro de la Cabeza are more dispersed, and probably suggest a use of this tomb for a few generations at least (Fig. 45). Present evidence perhaps suggests a period of use of several centuries for the La Pastora stone-vaulted tholos (Fig. 48), although in the absence of radiocarbon dates on human remains from the chamber this estimate does not, perhaps, necessarily relate to the primary use of the tomb.

Overall, the habitual mode of burial at Valencina was of ‘short’ duration. Funerary structures, even when collective inhumations were deposited in them, were generally used for a single event, a few decades, or a few generations at most. It seems that, usually, funerary structures did not outlast the active remembrance of their construction within the community. Structures which are likely to have been in use for more than a century are rare: the artificial cave at La Huera (Fig. 5), the collective burial pit Structure 64 in Sector IES (Fig. 13), and possibly La Pastora (Fig. 48). The deposit at Structure 1 of Calle Trabajadores N^os^ 14–18, by contrast, corresponds in all likelihood to a single event in which the remains of people who likely died simultaneously were deposited concurrently.

This duality (or variability) of tempos in the use of monuments and burials has already been noted in the Neolithic period of the British Isles (Whittle et al. 2011), but is now demonstrated at Valencina for the first time. Figure 54 illustrates the wide disparities in the temporalities of all the sectors and features or structures studied as part of this project. The issue of the time span over which the funerary structures were used is of special interest regarding the Montelirio tholos. Anecdotally, when excavations of this monument began in 2007, the Spanish media reported extensively on comments (intended just as informal remarks) by one of the team members, who claimed that the individuals buried in the main chamber (mostly women) may have formed part of the ‘grave goods’ of an important individual buried there, in a scenario similar to the tomb of Queen Pu-Abi from the Third Dynasty of Ur, in Mesopotamia. This sensational idea immediately gained enormous traction in the media, so much so that in July 2007 a piece reporting these comments was one of the ten most viewed items in the online version of *El País* (the most widely-circulated newspaper in Spain, and, in its online version, across the Spanish-speaking world), a feat never before or since accomplished by a Spanish archaeological investigation. However, this hypothesis has never been formally defended in a scientific publication.

The Montelirio bioarchaeological study (Pecero Espín 2016) shows that primary burials were found across the entire extension of Montelirio’s large chamber, except in the disturbed areas; disarticulated bone material along the perimeter of this chamber, which is typical inside Copper Age collective burials, was not found (Pecero Espín 2016). Moreover, anatomic proximity suggests a similar rate of skeletonisation for at least a large part of the 20 identified individuals (Pecero Espín 2016). Overall, the meticulous examination of bone stratigraphy and anatomical proximity prevented the author of this report from ruling out some degree of synchronicity in the formation of the deposit, as skeletonisation rates are simultaneous or very similar (Pecero Espín 2016). Consideration must also be given to the special demographic structure of the group of individuals buried at Montelirio, which includes 15 confirmed or likely female individuals and five individuals of unspecified sex, all of adult age (between 20 and 35 years of age) except for two subadults (Pecero Espín 2016). In addition, individual UE 101 presents a remarkable case of polydactylism (six toes) on both feet (Pecero Espín 2016), a visible physical feature that must have significantly marked the individual’s social persona. In short, therefore, the anthropological report suggests that the Montelirio burial deposits represent a non-random selection of the social group that constructed the tomb and carried out the inhumations. The report concludes that the inhumations may have been synchronic or may have taken place within a relatively short period of time (see García Sanjuán et al. 2016 for an expanded discussion).

The study presented here is the first attempt to establish the temporality of the use of the Montelirio tholos through radiocarbon dating and formal modelling (see also Bayliss et al. 2016, in Spanish). As previously explained, the Bayesian radiocarbon model suggests that the use of this tholos began in *2875*–*2700* *cal BC* (*95%* *probability*; *start: Montelirio Tholos*; Fig. 21), probably in *2850*–*2805* *cal BC* (*38%* *probability*) or *2765*–*2715* *cal BC* (*30%* *probability*), and ended in *2805*–*2635* *cal BC* (*95%* *probability; end: Montelirio Tholos*; Fig. 21), probably in *2765*–*2730* *cal BC* (*18%* *probability*) or *2725*–*2665* *cal BC* (*50%* *probability*). This represents a time span of up to *200* *years* (*95%* *probability*; *use: Montelirio Tholos*; Fig. 22), probably of *up to 100* *years* (*68%* *probability*), within which activity took place inside the tomb. These estimates allow the possibility that this monument was used for a short period of time (some decades) but do not prove that all of the burials at this megalith were the result of a single burial event. Therefore, although the chronometric model backs the conclusions of the anthropological study in general terms, it is not possible to specify the period of use of the tomb beyond one or possibly two centuries. Montelirio therefore remains a burial deposit formed over a short period, although not as short as, for example, Structure 1 at Calle Trabajadores N^os^ 14–18 (Fig. 29).

Whether or not the funerary deposition at the Montelirio tholos was quick and short, the data currently available do not allow us to make any specific social or cultural association between the individuals who were buried in it. There may be several possible explanations for the temporal proximity or even the simultaneity of the burials, including death owing to disease, episodes of violence (war) or rituals (human sacrifices). In fact, the literature on the Iberian Copper Age already includes cases of simultaneous (or partially simultaneous) collective burials attributed to this range of factors, such as those at Longar (Viana, Navarra) (Armendáriz and Irigaray 1995) and San Juan Ante Portam Latinam (Laguardia, Navarra) (Vegas Aramburu 1992, 2014).

#### Tholoi

The chronological models presented here also allow for a new analysis of the development of the monumental architecture at the site. Traditionally, tholoi are considered characteristic of the Iberian Copper Age as a whole. However, the paucity of available radiocarbon dates has until now prevented the study of the development of this type of monument over the third millennium cal BC (García Sanjuán et al. 2011; Aranda Jiménez and Lozano Medina 2016). The new evidence presented here suggests a possible temporal evolution of the tholos architecture that has such an extraordinary expression at Valencina.

The earliest tholoi seem to be Structure 10.042–10.049 from the PP4-Montelirio sector, which was constructed in *3725*–*3555* *cal BC* (*9%* *probability*; *start: Structure 10.042 (PP4*-*Montelirio);* Fig. 19) or *3515*–*2875* *cal BC* (*86%* *probability*), probably in *3185*–*2880* *cal BC (68% probability*), and the tholos at Cerro de la Cabeza, which was built in *3495*–*2710* *cal BC* (*95%* *probability*; *start: Cerro de la Cabeza*; Fig. 44), probably in *3035*–*2865* *cal BC* (*61%* *probability*) or *2840*–*2790* *cal BC* (*7%* *probability*). It is uncertain which of these monuments was constructed first (it is *68%* *probable* that Structure 10.042–10.049 at PP4-Montelirio was constructed before the tholos at Cerro de la Cabeza [Supplementary Table S1]), although it is clear (*93%* *probable*) that both were built before Montelirio in *2875*–*2700* *cal BC* (*95%* *probability*; *start: Montelirio Tholos*; Fig. 21), probably in *2850*–*2805* *cal BC* (*38%* *probability*) or *2765*–*2715* *cal BC* (*30%* *probability*). Several features differentiate these three structures. First, Montelirio is much larger than the other two. Secondly, both Montelirio and Structure 10.042–10.049 have two chambers, while (as far as could be ascertained, given the degree of destruction it endured before archaeological excavations commenced), the tholos of Cerro de la Cabeza only had one. These three megalithic constructions were interpreted as tholoi by the excavators, despite the fact that no vestiges of stone-corbelled roofing were found in any of them. In fact, the careful geological study of the Montelirio stratigraphy led to the somewhat surprising conclusion that the roofing of the chambers was made from a mixture of sun-dried clay and marl. Unequivocal vestiges were found in the form of both the remnants of construction materials and post holes that, distributed circularly around both chambers, putatively served to support the frame or scaffolding used while the clay dome hardened (Fernández Flores and García Sanjuán 2016). Domes made from clay and (possibly) from perishable materials had previously been postulated in the case of Iberian Chalcolithic tholoi in which no clear vestiges of collapsed stone used for corbelling had been found. However, this hypothesis had not been proven until the study of Montelirio. The absence of collapsed corbelling stones in Structure 10.042–10.049 and at Cerro de la Cabeza opens up the prospect of domes made from sun-dried clay.

Additionally, the new dating suggests that La Pastora was built, with stone corbelling, in *2755*–*2465* *cal BC* (*95%* *probability*; *start: La Pastora*; Fig. 47), probably in *2615*–*2480* *cal BC* (*68%* *probability*). This is clearly later than the construction of Structure 10.042–10.049, Cerro de la Cabeza, and Montelirio (*99%* *probable*). In his study of the orientations of Iberian megalithic constructions, Michael Hoskin (2001, pp. 78–80) recorded an orientation of 243^o^ for La Pastora, facing towards sunset. This is an exceptional orientation, as the vast majority of southern Iberian megaliths face towards sunrise. In fact, Hoskin proposed that La Pastora may have had a stellar orientation rather than a solar one; if the monument had been constructed between 2300 and 2200 cal BC, this would have made viewing the Sirius star from the inside of the chamber a possibility. The results of the chronological modelling presented here (Fig. 47) do not support the construction date proposed by Hoskin.

The orientation of all of these megaliths may be relevant. Although the orientation of the Cerro de la Cabeza tholos could not be established, as the monument was badly destroyed before archaeological excavation took place, both Structure 10.042–10.049 and Montelirio present ‘canonical’ sunrise orientations that follow the Neolithic tradition. Just as La Pastora faces sunset, Matarrubilla, the other great tholos with a stone corbelling in Valencina, presents an unusual orientation facing towards the north, at 17^o^. This suggests a possible architectural evolution of the tholoi in Valencina from early constructions of sun-dried clay domes and solar orientations (Cerro de la Cabeza, Structure 10.042–10.049 and Montelirio) to constructions of stone vaults and non-solar orientations (La Pastora, Matarrubilla). If proven, this shift could suggest potentially profound differences in ideology and cult, possibly connected to changes in social organisation, over the course of the use of Valencina. (At the nearby sectors of Señorio de Guzmán/Divina Pastora [Arteaga Matute and Cruz-Auñón Briones 2001; López Aldana et al. 2015], Los Cabezuelos [Arteaga Matute and Cruz-Auñón 1999a, b], El Roquetito [Murillo Díaz et al. 1990] and Área 9 of Castilleja de Guzmán [Méndez Izquierdo 2007], several other tholoi were found, displaying various orientations and building characteristics, although only preliminary reports are available.) This could also help us interpret other major Copper Age monumental landscapes, such as Antequera (Málaga), where El Romeral, the largest Iberian tholos, also displays an anomalous, non-sunrise orientation (García Sanjuán, Moreno Escobar et al. 2016b).

#### Ditches

Another major element in the expression of monumentality at Valencina are the ditches. Multiple segments of ditches (often large in size) have been identified at different sectors of the site, although to date no major complete enclosure circuit has been found. The only confirmed ‘enclosure’ at Valencina is Structure 10.024 of the PP4-Montelirio sector. Although incomplete (part of it was cut by Castilleja de Guzmán’s Miguel de Cervantes Saavedra Street), this enclosure has a maximum diameter of c. 17 m, with a possible entrance on its southern side. Inside the enclosure and also cutting the ditch, pits (mostly circular in plan) were found. These features yielded a large quantity of faunal remains, which amount to 95% of those found in the entire PP4-Montelirio sector (Liesau et al. 2014, p. 77) as well as a large quantity of pottery (Mora Molina et al. 2013, p. 274). In fact, in some sectors, like Plan Parcial Matarrubilla and Parcela Municipal, different ditches are laid at right angles to one another, which does not suggest that all of them formed coherent, discrete enclosures. Hypotheses for their use include possible roles as irrigation ditches (Fernández Gómez 2011) or defensive systems that delimited residential or storage areas of a ‘fortified’ settlement (Nocete Calvo 2001, p. 137). Another possibility is that the Valencina ditches (and perhaps enclosures, if they formed them) were part of collective labour investments carried out as part of social practices involving the periodic assembly or gathering of large numbers of people. If so, then this monument ‘system’ would not have been designed and carried out at once, nor meant to be used or ‘experienced’ as a whole. At Camino de Las Yeseras (San Fernando de Henares, Madrid), the only Iberian Copper Age ditched enclosure for which a Bayesian chronological model is available, ditches have been argued to be the result of a mobilisation of labour that was intermittently applied by different generations (Balsera Nieto et al. 2015, pp. 151–153). Despite limitations and qualifications, the data obtained at Camino de Las Yeseras suggest that none of the ditches there were used for more than *40* *years* (*95%* *probability*; Balsera Nieto et al. 2015, Fig. 8).

**Fig. 57:**
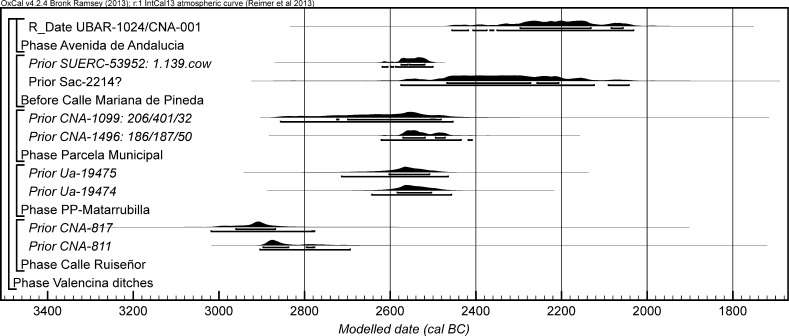
Probability distributions of dates from ditches at Valencina de la Concepción, derived from the models defined in Figs. 25, 31, 40 and 51 (Sac-2214, which is included from the model shown in Fig. 25, and UBAR-1024/CNA-001 have simply been calibrated)

There are currently nine radiocarbon dates on samples from ditches at Valencina (Fig. 57), seemingly spanning most of the third millennium cal BC (although only ditch 3 at Avenida de Andalucía N^o^ 9 falls in its second half), suggesting that these features were a very prominent part of social practice at the site. The quality of the data (mostly because of problems with the context and nature of the samples) prevents the establishment of a detailed chronology of the cutting and filling of those ditches, with the possible exception of Structure 1 at Calle Mariana de Pineda s/n. At the bottom of this ditch the articulating bones of a cow were found, whereas the upper part of the infill was later re-cut by a pit in which the remains of 18 individuals were deposited (no laboratory anthropological report is available yet). The chronological model, based on one date on the cow bone and three dates on human bone, suggests that the ditch was open and began to be filled in around *2620*–*2605* *cal BC* (*SUERC*-*53952: 1.139.cow*; *4%* *probability*; Fig. 25) or *2600*–*2500* *cal BC* (*91%* *probability*), probably around *2575*–*2515* *cal BC* (*68%* *probability*). It was certainly filled by the time Structure 30 was cut in *2600*–*2495* *cal BC* (*95%* *probability*; *build: Structure 30 (Calle Mariana de Pineda)*; Fig. 25), probably in *2575*–*2565* *cal BC* (*3%* *probability*) or *2555*–*2505* *cal BC* (*65%* *probability*).

Although there is much more to learn about the chronology of the ditches at Valencina, at least this is a start. Two observations are potentially significant. Despite our inability yet to model the date of ditch construction at Calle Mariana de Pineda s/n, the timespan of its infilling may have been relatively short, perhaps less than a single generation (*1*–*30* *years*; *95%* *probability*; *span: Ditch open*; Fig. 26, probably *1*–*15* *years* (*68%* *probability*)), which is basically coincident with the results obtained at Camino de Las Yeseras (Balsera Nieto et al. 2015, Fig. 8).

It may also be no accident that this activity falls late in the history of the site. There is no evidence that the ditches are ‘defensive’ in the traditional sense, but the mortuary activity at Calle Mariana de Pineda s/n and Calle Trabajadores N^os^ 14–18 may suggest swifter and—in the case of the latter—more dramatic events, so at least some of the ditch-digging at Valencina might represent a cultural response to a period of social instability.

#### Social Differentiation Through Time

A final conclusion emerging from our study is that human activity at Valencina de la Concepción underwent significant changes between its origins and the end of the Copper Age way of life there. When compared with other more monumental tombs, the grave goods found at the hypogea of La Huera and Calle Dinamarca N^os^ 3–5 do not feature the sophisticated objects manufactured with exotic raw materials that we see in tomb 10.042–10.049 or at the Montelirio tholos. In this respect, the data obtained in this study are a true cautionary tale against any simplification in the analysis of the potential social complexity at Valencina. Some of the structures with ‘poor’ contents and the tholoi with ‘rich’ contents may well correspond to chronologically separate events and changing social environments, in which case they do not necessarily prove variations in wealth, status or prowess between contemporary individuals or groups. In addition, the evidence obtained in the Large Chamber of the Montelirio tholos, which is the ‘wealthiest’ tomb ever found in Valencina, now supported by a strong chronological model (Bayliss et al. 2016 and this paper), has been interpreted as the likely expression of a group of religious specialists (possibly priestesses) quite unlike the ruling ‘class’ of an ‘aristocratic’ type that was predicted in the Copper Age ‘state’ hypothesis (García Sanjuán, Fernández Flores and Díaz-Zorita Bonilla 2016a, pp. 539–547).

The tradition of simpler mortuary practices also appears to continue throughout the entire biography of Valencina alongside that of the large tholos tombs, in the form of both single and collective burials in simple pits (Fig. 55), and therefore variations in resource mobilisation must have existed between factional groups, clans or communities using the site as a burial ground. The extent to which that can be interpreted in terms of the rise and development of complex forms of social organisation depends, first and foremost, on our ability to establish their precise temporality. In fact, a thorough ‘temporal de-construction’ of the site is needed before its nature, and the associated social complexity of which it was part, can be ascertained. That is perhaps best now explored in narrative sequence.

### Towards an Interpretive Narrative

Despite its imperfections, the evidence presented in this paper provides, for the first time, a more robust and more precise chronology for the development of human activity within Valencina, from the later fourth to the late third millennium cal BC. In the broad terms put forward by Bob Chapman (2008, p. 236), this corresponds to the suggested spans of an Initial Copper Age and a Full Copper Age, and the start of a Recent Copper Age, as noted at the start of the paper. We can now move beyond this kind of periodisation and begin to consider the implications of the more robust chronology presented here for the timing, order and duration of things, and the tempo of change. Unavoidably, since the Bayesian approach is both interpretive and iterative (Bayliss 2009; Bayliss, Bronk Ramsey et al. 2007), this is all provisional, but it marks the beginning of a profound shift away from fuzziness. In line with the overall aims of this paper, we retain a basic chronological structure for what follows: an attempt to outline the main trends in the development of Valencina.

There is no evidence of Late Neolithic activity at Valencina. In fact, across the lower Guadalquivir valley the settlement patterns of the whole Neolithic period (c. 5400–3200 cal BC) are rather poorly documented. There are published references to sites along the Corbones river, a tributary of the Guadalquivir on its left bank (Fernández Caro 1995), and around the old marine gulf today silted by the Guadalquivir sediments (Escacena Carrasco 2011). The only evidence from excavation comes from Los Álamos, a small open-air settlement near Fuentes de Andalucía (Arteaga Matute and Cruz-Auñón Briones 1999a). A very interesting site, however, is La Marismilla, in the municipality of Puebla del Río, some 25 km south of Valencina, where production of marine salt, which the excavators dated to the Late Neolithic and Early Copper Age, was recorded (Escacena Carrasco et al. 1996). Although no radiocarbon dates are available for La Marismilla, it has been suggested that salt production may have played a prominent role in the rise of Valencina as a major central place across the lower Guadalquivir valley in the late fourth millennium cal BC (García Sanjuán 2013, p. 50).

Although there is still little known about the cultural and social processes leading to it, by the last centuries of the fourth millennium cal BC Valencina started to be used as a burial place by local communities. Beginning in the later 32nd century cal BC, and continuing for well over a century, the ‘artificial cave’ of La Huera presents successive, collective burial of a rather unostentatious kind. The main focus of the mortuary rite appears to have been the deposition of intact corpses, one by one. Grave goods were not a prominent part of mortuary rites, and when selected for deposition, were not particularly distinctive. Skeletal remains were not sacrosanct, and were moved or otherwise disturbed by succeeding depositions. It is striking that the well-dated sequence spans a considerable period. This could be seen as the focus of a small but persistent group, such as a family, lineage or other kin-based grouping, or some kind of co-resident neighbourhood. As with La Huera, the mortuary activity dated at Calle Dinamarca N^os^ 3–5 also appears to span a significant period, probably beginning in the 31st century cal BC (it is important to stress that neither Calle Dinamarca N^os^ 3–5 structure dated here was excavated to the bottom). The emphasis is again, however, on successive, collective burial, of a non-spectacular but enduring kind. The activity here overlaps in time with the use of La Huera, but ends later, in the generations around 2900 cal BC (Fig. 54). Dating the mortuary activity at IES is not straightforward, as distributions are bimodal, and the present state of the evidence is challenging. The activity could belong to the 29th or to the 28th century cal BC. Structure 64 at IES may represent an ongoing tradition of successive, collective and undemonstrative burial, though given the absence of a full anthropological report on this structure the possibility of further diversity in the form of deposition of secondary remains should be kept in mind.

The 29th century cal BC sees the appearance of more varied mortuary practices. The earliest sun-dried mud-vault tholoi may have been built at Valencina at this time, alongside a range of collective burial in simple pits (Fig. 55; Supplementary Table S1). Such diverse burial activity is seen in features in the PP4-Montelirio sector such as Structure 10.071, a shallow, circular pit without stone elements (with an MNI of seven showing a clear succession of burial) or Structure 10.031, another shallow, oval pit without stone elements (with an MNI of three and no grave goods except for a single object made of carved bone or ivory). La Alcazaba, La Cima and perhaps El Algarrobillo also belong to this long-lasting, largely simple but diverse tradition of collective deposition in pits. These contexts show a variety of sizes and forms of pits but none appear to be as large as the ‘artificial cave’ of La Huera or Calle Dinamarca N^os^ 3–5. There was seemingly a spectrum of kinds of deposition, from articulated or probably originally articulated corpses, to partial remains, possibly including crania on their own (if any of the single crania at El Algarrobillo come from the lower layer). The quantities of human remains vary, as does the abundance of other material. Arguments can be made about whether or not neat conceptual or terminological distinctions between ‘mortuary’ and ‘non-mortuary’ activity are possible, but it is clear that the formal deposition of human remains was at its peak intensity at this time (Fig. 56) and that it covered much of the Valencina landscape (Fig. 2). Additionally, it is worth noting that the overall emphasis appears to have been on collective rather than individual deposits, and on shared activity and assembly of various kinds.

The major innovation of this time, however, was the introduction of megalithic architecture, in the form of tholoi. The prominent positions which at least some of these megaliths took among other structures and features, the sometimes spectacular grave goods in some of them, and the wide exchange and cultural connections they reveal, which are all eye-catching and of fundamental importance, represent a major shift in the life of the site. Following the models presented in this paper, and taking into account the difficulties with precisely dating these specific structures, the early tholos horizon appears to be largely confined to the 29th and 28th centuries cal BC, and thus the significance of their appearance, character, temporality and decline deserve our full attention in a new chronological perspective. There may then have been a hiatus at Valencina in the construction and use of tholos tombs, before the tradition gained traction again some two centuries later in a different and perhaps ‘heterodox’ form (potentially reflecting changes in the prevailing cosmology) at La Pastora and Matarrubilla. The character, chronology, and context of this reawakening are discussed further below.

The new tholos architecture established at Valencina in the 29th century BC was in several ways different to all previous funerary architecture at the site. It certainly required the mobilisation of greater labour than required for, say, the construction of an artificial cave or a simple pit. It probably demanded a greater degree of planning (large slate slabs and sandstone capstones had to be brought from some distance), and more skill in the execution of more precisely laid out chamber walls and roofing, however that was achieved (as discussed above, the development of the technical ability to create corbelled chambers seems to have started with false domes made of sun-dried clay). It presented a new kind of space, compared to the previous local tradition of negative or underground features: partly cut into the ground, but detached from the everyday by the addition of mounds, and approached by formal passages or corridors. This is an architecture of formal separation, with the participants involved in mortuary and other rites bound by the choreography it promoted.

The various constructions grouped in the PP4-Montelirio and Montelirio sectors arguably constitute a defined formal disposal area (sensu Chapman 1981; Parker Pearson 1999) within the site as a whole. There was clearly considerable diversity here, including structures with and without stone elements, and megalithic tombs of various sizes and shapes, including two-chambered ones like Structure 10.042–10.049 and the Montelirio tholos. But there may well also have been principles of spatial order, behind which there could have been further structuring notions of, say, seniority and relative ranking between individuals and kin groups. The space appears dominated by the largest tholoi, such as Structure 10.042–10.049 and, especially, Montelirio. It would be easy to assume that these could have been built first, with successors then clustering around them. However, it is salutary to remember the case of Knowth in the late fourth millennium cal BC, much further afield in eastern Ireland, where the main mound filled in a space established by smaller predecessors (Eogan 1986; Hensey 2015). Given that the start of activity at the Montelirio tholos is slightly later than in 10.042–10.049 and 10.071 in PP4-Montelirio (*99%* and *98%* *probability*, respectively, that the start of the two PP4-Montelirio features pre-date *start: Montelirio Tholos*), the dynamics of the use of space in the formal disposal area were probably fluid.

**Fig. 58:**
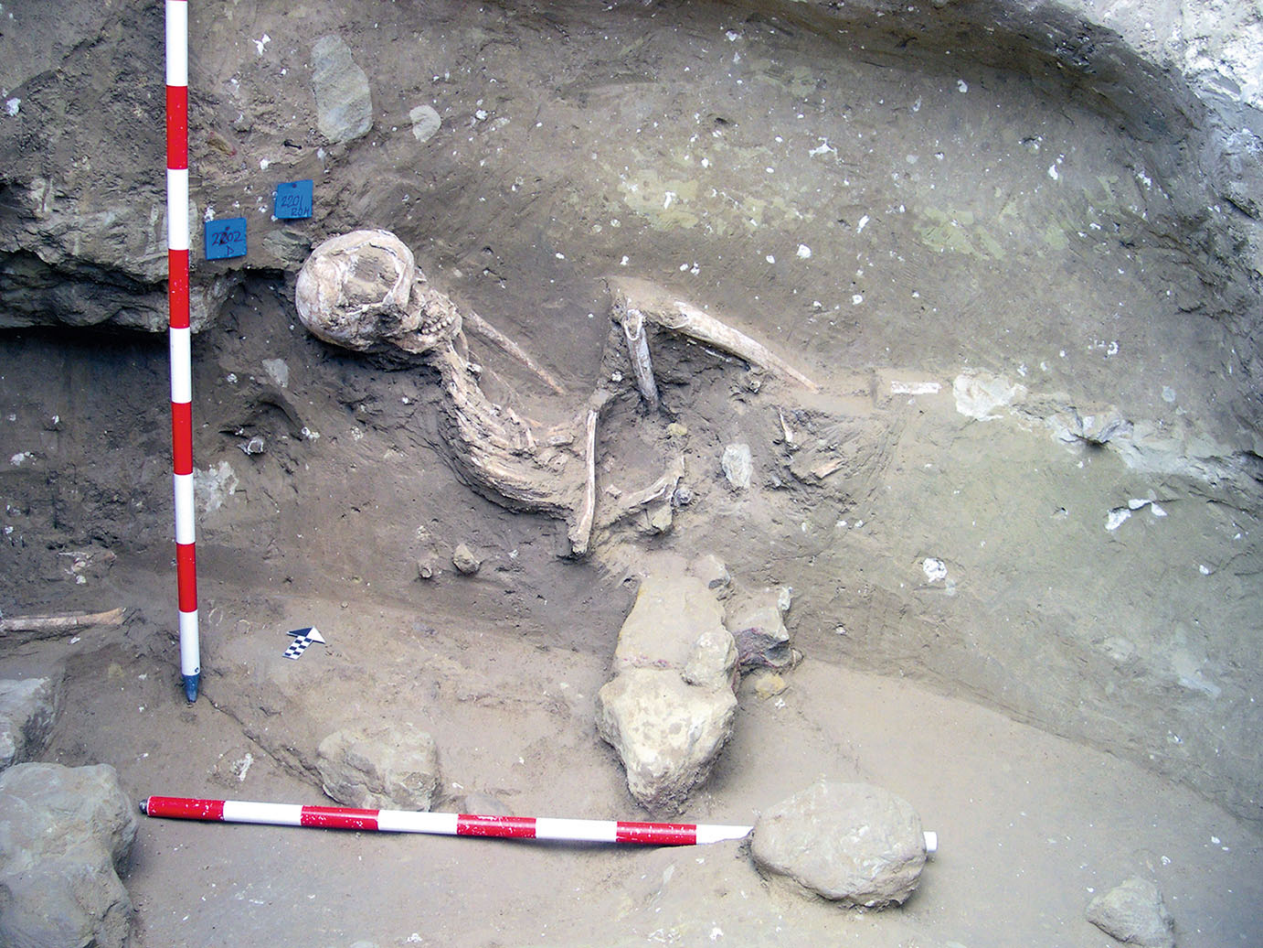
La Huera. Individual UE 2201 inserted into the top of the old deposits. Photo: Elena Méndez Izquierdo

The older suggestion of a sharp distinction between a ‘domestic’ zone and a ‘necropolis’ area within Valencina as a whole has already been challenged, for good reasons (Costa Caramé et al. 2010; García Sanjuán 2013). But in the horizon of tholos use suggested here, there could have been a concentration of tholoi and related features in one part of the site—subject, of course, to the reservation that there remain substantial areas of it, to the north, the northeast and the south, which have not yet seen extensive investigation. Cerro de la Cabeza, located in the northern part of the site, demonstrates the probable existence of tholos architecture outside the cluster in the southeastern sector (presided over by Montelirio), but the now-widespread investigations across the other sectors reported here show that there are few signs of a general scatter of tholoi through Valencina as a whole. If this stands up to further investigation, is it perhaps just coincidence that the new tholos architecture was concentrated in the eastern part of the site, close to where the currently earliest feature was located—at La Huera? And is it just further coincidence that the last corpse (Individual UE 2201) was inserted in La Huera into the top of the old deposits (Fig. 58) in *2920*–*2860* *cal BC* (*88%* *probability*; *SUERC*-*47677: 2201*; Fig. 4) or *2805*–*2760* *cal BC* (*7%* *probability*), probably in *2905*–*2875* *cal BC* (*68%* *probability*), after a measurable interval since the last use of the artificial cave, and perhaps at about the time when the first tholoi in the PP4-Montelirio and Montelirio sectors were built?

As well as a new spatial order, new kinds of temporality may now have been promoted. These could have worked in two ways. One new emphasis shifts to the immediate present, at the scale of the events of construction and individual funerals. Another new temporality may have marked the introduction of genealogical projections into remoter pasts.

The focus in the new, spectacular tholoi was not only on the large-scale architecture—with its emphasis on large and beautifully-dressed slate slabs, probably covered in bright red cinnabar-based pigment (and, at least in the case of Montelirio, painted and engraved with a wealth of graphic motifs)—but also on far more elaborate grave assemblages. The prime examples are 10.042–10.049 in PP4-Montelirio and the Montelirio tholos, with their spectacular assemblages of exotic materials and finely crafted objects. Regardless of whether the people in the Montelirio tholos were all deposited at the same time, we can think in terms not just of the burial of individuals but also of assembly and display. Some of these corpses were dressed in elaborate shrouds or costumes, to judge by the concentrations of perforated beads (of limestone, shell and amber) in the Montelirio tomb. Small gold foil as well as amber and ivory ornaments could also have been worn on or otherwise accompanied the dead body—but an alternative interpretation of the gold foils, based on the largest example ever found in Iberia, is possible (Murillo-Barroso et al. 2015). Interestingly, inside the main chamber of Montelirio it is impossible to establish clear-cut patterns of association between individuals and artefacts, other than the ceremonial attires or mantles made with thousands of perforated beads found on top of some of them. Directly connected with the individual inhumed in the lower level in Structure 10.049, however, was an impressive array of things, including an ivory tusk, flint blades, one flint dagger and amber pommel, an ivory bowl and other small ivory objects, which were clearly intended as the grave goods of a socially distinctive man, possibly a high-ranking individual, or someone with particular wealth (perhaps connected with the exchange or manufacture of ivory, as the whole tusk might suggest) (García Sanjuán et al. 2018). Above this individual, although separated by a carefully arranged cover made of horizontally-laid slate slabs, there were yet more objects, including (and it is worth listing them again) an ostrich egg shell, five complete or semi-complete ceramic plates, an astonishingly sophisticated rock-crystal dagger blade, 38 flint blades, an arrowhead, some beads, and various ivory objects, including the hilt of the dagger and a rigid support for its sheath, a heavily-decorated segment of a tusk, a vessel and fragments of a possible bracelet.

Even if the architecture of this early tholoi tradition would have severely restricted attendance at the final moments in which the dead were laid to rest, it is hard to imagine that such notable conjunctions of things and bodies were not also the occasion for highly visible procession and public display. Such attention to the event of the funeral seems to contrast strongly with the longer-scale persistence of previous modest mortuary practice.

Some of this at least is surely evidence of increasing social differentiation among the communities that lived at, near, or within range of Valencina in the early part of the Copper Age.

What the ‘showy’ grave goods suggest is that at some point in the evolution of the local Copper Age communities, perhaps in the 29th century cal BC, some individuals or groups (kin or corporate) began to have access to foreign raw materials which were to be transformed through highly specialised and skilled techniques (demanded by, for example ivory carving and rock-crystal knapping). Many of the things and materials left inside the Montelirio tholos and Structure 10.042–10.049 came from far beyond Valencina de la Concepción, from within Iberia, north Africa and perhaps the central Mediterranean, and even the Levant (Murillo-Barroso and García Sanjuán 2013; García Sanjuán, Luciañez Triviño et al. 2013a; García Sanjuán, Fernández Flores and Díaz-Zorita Bonilla 2016a). Such origins readily evoke the power of the distant and the exotic, which Mary Helms (1988, 1998) in particular has shown can be an effective means to bolster the social position, individual charisma and mystique of self-aggrandising individuals. In this case, geographical distance could also have gone hand in hand with temporal distance. Although the immediacy of individual funerals has been emphasised above, another, new, dimension of their temporality may have rested in claims to control of the past. At the same time, there may also have been economic changes that enabled the concentration of influence, wealth or authority within these groups or elites. Among the exotic grave goods of Montelirio are several figurines of pigs, including a carved ivory comb, and acorns (at least eight carved in ivory and one on amber) which may suggest a particular emphasis on pig production and pannage among those represented (García Sanjuán 2017). It is possible to imagine that early adopters of pig farming and pannage production would find themselves able, for the first time, to generate large amounts of meat quickly, which may have been convertible into both economic and social capital (through, for example, conspicuous consumption or feasting), which in turn may have accelerated—or even driven—a process of rapid social differentiation.

It is also tempting to think of the individuals or small groups of individuals prominently displayed in these funerary send-offs as the founders of more tightly defined descent groups, such as lineages, and subsequent burials may have taken up position in relation to them. The earlier tradition of successive burial was probably, as argued, making reference to the past, but it is possible that both more precise and more extended genealogical reckoning was now being attempted in the new style of mortuary rite. It has been suggested that Iberian Late Neolithic and Copper Age plaques were material devices aimed at maintaining and transmitting information regarding genealogical descent (cf. Lillios 2008). The distant in space and the remote in time thus could have met in the demonstrative singularity of individual funerals. This is not to describe the whole of tholos-associated rites; diversity again seems to apply. We know little of the contents of Cerro de la Cabeza, but Structure 10.071 at PP4-Montelirio certainly appears not only to have rather less dramatic deposits than those just discussed, but also echoes the earlier tradition of collective, successive burials. Structure 10.031 also presents a seemingly simple, small collective deposition which contained just one artefact: a staff or baton decorated with geometric patterns not unlike those frequent in idols. (Since this object is significantly fragmented and eroded, it has so far been impossible to establish whether it is made of human or animal bone. Some precedents exist in Iberia for Copper Age symbolic objects made of human bone [Delibes de Castro and De Paz Fernández 2002].)

Given the restricted numbers of assemblages of exotic and finely crafted paraphernalia, confined to a limited number of larger tombs, the architecture of which evokes prominence and pronounces separation, it is hard not to think of individuals or groups whose aim was to promote themselves above others. In this sense, the 29th and 28th centuries cal BC at Valencina de la Concepción seem to have been characterised by quite intense social competition. Whether such potential self-aggrandisers were also competing among themselves (rather than with more distant neighbours, or rivals in far-flung exchange networks) will depend on establishing more robust chronology in the future. From the modelling, it appears that the activity in the Montelirio tholos probably started slightly later (*99%* *probability* that *start: Montelirio Tholos* is later than *start: Structure 10.042 (PP4*-*Montelirio*)) than that in Structure 10.042–10.049, so while emulation of predecessors could have been a factor, it is not easy to establish direct competition between exactly contemporary prominent individuals. In this context, it has been suggested that the upper layer (later deposition) of Structure 10.049 presents subtle hints (in the form of a long barbed arrowhead, an ostrich egg-shell and perforated beads, absent in the lower layer: the earlier deposition in this tomb) of connection with the exceptional craftsmanship present in the nearby Montelirio tholos, which would point to the possibility of a ‘genealogical’ connection between these two major monuments (García Sanjuán, Fernández Flores and Díaz-Zorita Bonilla 2016a; García Sanjuán et al. 2018).

The suggestion of social competition in both Valencina and southern Iberia in the first half of the third millennium cal BC is hardly new. Since the early claim of factional competition at Los Millares (Chapman 1990), the debate has been rather polarised between proponents of something along the lines of an early state or class society (Cruz-Auñón Briones and Arteaga Matute 1996, p. 599; Nocete Calvo 2001, p. 95; Afonso Marrero and Cámara Serrano 2006, p. 143; Nocete Calvo et al. 2008, p. 731) and the view of emergent but unstable elites, with limited capacity to break up traditional Neolithic bonds of collectiveness and communality (García Sanjuán 2006; Chapman 2008, p. 243; Díaz-del-Río 2011; García Sanjuán and Murillo-Barroso 2013, pp. 133–135). The evidence reviewed in this paper strongly suggests that, for the case of Valencina de la Concepción at least, the latter kind of scenario better applies, whatever terminology might best be used. We could talk of big men, self-aggrandisers, trans-egalitarian societies, aristocrats or chiefs, for example, but each of these terms has connotations that can vary in important ways (Flannery and Marcus 2012). There is no evidence for the scale of difference, the levels and control of production, or the institutionalisation of coercive power that the early state model requires. The formally modelled date estimates at the heart of this paper support the notion of a finite surge in efforts to display prominence and difference, through labour mobilisation, through exchange and acquisition of exotic paraphernalia (among which copper objects are remarkably absent), and through funerary architecture and ostentatious funerals, whose main motives seem to have been to show off on a grand scale and perhaps to be seen to control the past. But this is all concentrated in the mortuary sphere. There is no clear evidence of any domestic or residential special arrangements for people like the individual inhumed in Structure 10.049, or for large public buildings or central plazas. Finely crafted objects made of ivory, amber or rock-crystal were deposited with the dead in the realm of putative ancestors and remoter time, but other valuable objects, such as the large gold foil with oculi found in Structure 10.029 of the PP4-Montelirio sector, were not claimed as grave goods by any particular individual. Instead, they appear to have been used as non-funerary votive offerings in a more general sense. This seems to show that the capacity of aggrandisers or elites to hoard wealth was limited by social restrictions. In addition, there is no clear sign of institutionalised hoarding or the accumulation of valuables for their own sake. By way of comparison, there are ethnographic cases where activity by elites is tolerated, as long as it brings renown and benefit to society as a whole (Flannery and Marcus 2012, pp. 183, 206), and perhaps the jostling in the mortuary domain at Valencina is a manifestation of this kind of tension between an emerging elite and the rest of society. If that is the currently most plausible interpretation of developments at Valencina, it still leaves open the possibility of regional variation across southern Iberia (cf. Chapman 2008, p. 247), to which we briefly return at the end of this paper.

None of the four non-funerary sectors that have radiocarbon dates provide evidence for occupation on the site contemporary with the first funerary activities at the end of the fourth millennium cal BC (Fig. 54). Activity at Calle Ruiseñor appears to fall principally in the 30th and 29th centuries cal BC (Fig. 31) and was thus contemporary with the first intense period of funerary practice apparent in Fig. 56. The non-funerary activity at Parcela Municipal has been dated imprecisely, but it may have started at this time (Fig. 51). It certainly seems to have continued into the 26th century cal BC. Ivory working at IES (402/403; Fig. 12) also probably dates to the period when burial was less intense on the site. The main period of occupation at Plan Parcial Matarrubilla was probably confined to a relatively restricted period of a few generations, centred on the 26th century cal BC (Fig. 40). Since more than half the dated samples were fragments of charcoal retrieved either from furnaces or from within fragments of copper slag, the activities in this sector at this time certainly included copper working. According to our analysis, the evidence for copper working at PP-Matarrubilla appears to have started after the period when sun-dried mud-vault tholos tombs, and particularly Montelirio, were in use on the site (*85%* *probable* that *start: PP*-*Matarrubilla* [Fig. 40] is later than *end: Montelirio Tholos* [Fig. 21]), and before the stone-vaulted tholos at La Pastora was constructed (*69%* *probable)*. The modelling of 16 of the 18 radiocarbon dates from Plan Parcial Matarrubilla places the activity there between *2815*–*2495* *cal BC* (*95%* *probability*; *start: PP*-*Matarrubilla*; Fig. 40), probably *2660*–*2540* *cal BC* (*68%* *probability*), and *2570*–*2425* *cal BC* (*95%* *probability*; *end: PP*-*Matarrubilla*; Fig. 40), probably *2545*–*2465* *cal BC* (*68%* *probability*). This revised dating is of special relevance, given that the interpretation by Nocete Calvo et al. (2008) is that the main copper production at PP-Matarrubilla had occurred between 2750 and 2500 cal BC, which would have made it contemporary with the Montelirio tholos, where not a single copper artefact was found. In fact, according to our revised chronology, the copper smelting activity at PP-Matarrubilla is very probably later than the use of the Montelirio tholos. The earliest copper item from the site may be the small object (of no recognisable form) found at the lower level of Structure 10.049 in association with a single adult male inhumation. Using the chronology of Structure 10.042 as a proxy (all attempts at dating Structure 10.049 directly through radiocarbon determinations on human bone or ivory samples having failed), this would probably be earlier than either the use of the Montelirio tholos or the smelting activity at PP-Matarrubilla.

In fact, there are many unresolved issues regarding the activity recorded at PP-Matarrubilla, the largest sector ever excavated at the site. The study by Nocete Calvo et al. (2008) focused only on the copper smelting remains, but the activity at this sector must have been much more complex than that. Two of the dates included in that timespan come from a ditch (Fig. 57), whereas various other dates were obtained from animal bone, which raises a number of questions: what exactly was the connection of the ditches with copper smelting? Did smelting take place ‘inside’ the ditches, or were the ditches used, as claimed (Nocete Calvo et al. 2008), to ‘dump’ the smelting debris? If so, were the ditches cut only for that purpose? If the ditches of the PP-Matarrubilla were not cut (and used) to serve only as ‘dumping’ places, what other roles did they have? Furthermore, how are the faunal remains connected to the smelting activity? The study of the small assemblage of 789 animal bones found in this sector revealed the consumption of bovids, suids and ovicaprids (Abril López et al. 2010). The authors of this study suggested that ‘the faunal assemblage reflects some degree of selective transportation, since the small presence of phalanxes, bones with high osseous density, seems to suggest that they were previously discarded in the butchery areas’ (Abril López et al. 2010, p. 97; our translation from the original Spanish). Interestingly, the study of the faunal assemblage found at Calle Mariana de Pineda s/n reached the exact same conclusion: ‘the predominance of remains belonging to limbs, which are by far the anatomical parts that provide most of the meat, together with the relative scarcity of elements belonging to hoofs … suggests the possibility of a selective transportation from the area where the butchering took place into the area where consumption effectively occurred’ (Pajuelo Pando and López Aldana 2013a, b, p. 452; our translation from the original Spanish). As has been suggested (García Sanjuán 2013, p. 42), it would be very interesting to examine whether these observations apply to faunal assemblages found at other sectors of Valencina, as they could be used to ascertain the degree of stability or temporality of the site’s occupation. If butchery did not take place where the remains were found, and the meatiest parts were brought from elsewhere, how far was the meat taken from? And what implications does this have for assessing seasonality or permanency in the occupation of the site? Were herds kept at the site or were they kept elsewhere, the meaty parts being brought onto the site only on specific occasions? Thus far, there have also been no significant synthetic studies of variation in food species represented in the different sectors and periods at Valencina that might shed light on the kinds of economic changes associated with the social changes we discuss above. Together with the potential for isotope studies of animal bones, this must be a priority for future work and it is to be hoped that the increasingly detailed chronological framework available might enable faunal remains to be investigated and synthesised more fully.

The evidence retrieved further afield in the lower Guadalquivir valley suggests intense activity around Valencina in the earlier third millennium cal BC. At Cortijo de Miraflores—a Copper Age settlement found on the left bank of the Guadalquivir river in what is today a park within the city of Seville, barely 8 km east of Valencina as the crow flies—four radiocarbon dates suggest activity at this period (Lara Montero et al. 2006). Four samples of charcoal (*Quercus ilex*) were dated from Structures I4/001 and I6/003, which calibrate at 2σ to 2880–2575 cal BC (KIA-24924; 4133 ± 39 BP), 3015–2895 cal BC (KIA-24925; 4327 ± 24 BP), 2865–2575 cal BC (KIA-24926; 4109 ± 28 BP) and 2890–2670 cal BC (KIA-24927; 4190 ± 28 BP) (Stuiver and Reimer 1986; Reimer et al. 2013). The only available date for Copper Age Carmona (some 25 km to the northeast of Valencina) gave a result of 2860–2805 cal BC (11% probability; Ua-21476: 4055 ± 50 BP; Nocete Calvo et al. 2011, Table 1) or 2755–2720 cal BC (4% probability) or 2705–2470 cal BC (80% probability). This result was produced on a fragment of *Quercus ilex* charcoal found within a lump of copper slag from metallurgical Structure E1 at Dolores Quintanilla 6. Recently, third millennium dates have also been obtained for a fascinating ditched enclosure system at Loma del Real Tesoro, near Carmona (Escudero Carrillo et al. 2017).

In fact, there is a plethora of Copper Age sites distributed throughout the lower Guadalquivir valley, a short distance from Valencina, for which, unfortunately, no radiocarbon dates are currently available. If Valencina acted as a central or gathering place for these communities, then it would make sense for animals not to be kept permanently at the site, but for the meat to be brought in for gatherings and assembly. Understanding this wider context must be a goal for future research.

By the time activity at PP-Matarrubilla ended, it is very likely that La Pastora had already been built (*83%* *probable)*. In addition, it is *74%* *probable* that the single inhumation at Parcela Municipal (*CNA*-*1499: 435/ROH437*; Fig. 51) is later than the construction of La Pastora.

All in all, the generations around 2900 cal BC seem to have been witness to the most intense funerary activity at Valencina—the flourishing of the site, as it were. At this time, no conclusive evidence has been found to affirm that permanent residence took place at the site at all—see discussion in García Sanjuán (2013) and García Sanjuán and Murillo-Barroso (2013). Likewise, no evidence is yet available to establish the role played by ditches (and perhaps ditched enclosures) within this period.

Timing and duration are again revealing in considering questions of discontinuity and a possible later re-invention of the tholos tradition. Although the estimates modelled in this paper are far from perfect, they suggest not only a relatively swift emergence of new rites, but also their relatively rapid decline (Fig. 55). The view that factionalism and tensions ‘*eventually*’ developed (Chapman 2008, p. 243, our emphasis) can now much more robustly be replaced by the interpretation of relatively quick change; perhaps only a few generations of ‘wannabes’, their supporters and peers could sustain—or tolerate—the efforts required to connect with the far-flung and to manipulate the local social and political context. Again much depends on our choice of language. Was this a rapid ‘collapse’ (sensu Tainter 2006)? Or was it more by way of a gradual subsiding of effort? More precision is required in future research, but it appears that the formal modelling has identified a potential ending, or hiatus, in the tholos tradition that was previously unsuspected.

After the initial phase of the mud-vault tholoi, there seems to have been a reduction in the intensity of funerary activity at Valencina which lasted for much of the 27th and 26th centuries cal BC, although it is not clear that this reduction was mirrored in the non-funerary activities on the site (Fig. 56). Burial activity intensified again, however, in the generations around 2500 cal BC.

This may be the time in which we should place the very big tholoi of Matarrubilla and La Pastora. The description of Ontiveros provided by Juan de Mata Carriazo y Arroquia (1962) suggests that the corridor of this monument is similar to that of Montelirio, with large slate slabs painted in red and covered by capstones occasionally separated by vertically placed slate lintels. No radiocarbon dates are available for either Matarrubilla or Ontiveros but the model shown in Fig. 47 suggests that La Pastora was constructed in *2755*–*2465* *cal BC* (*95%* *probability*; *start: La Pastora*), probably in *2615*–*2480* *cal BC* (*68%* *probability*). Activity in the atrium continued until *2485*–*1360* *cal BC* (*95%* *probability*; *end: La Pastora*; Fig. 47), probably until *2435*–*2035* *cal BC* (*68%* *probability*). We know something of the contents of Matarrubilla, in the form of ivory, gold and green stones (Obermaier 1919; Collantes de Terán 1969; Perea Caveda 1991; Schuhmacher et al. 2013; Odriozola Lloret and García Sanjuán 2013). Distinctive materials from La Pastora, such as the javelin or spear heads noted earlier, have traditionally been seen as a later re-use of the tholos mound, because the date inferred from their morphology was c. 2300–2000 cal BC (Mederos Martín 2000, pp. 94–95; Gernez 2011, p. 336). However, recent research suggests that this type of spear system may have originated in eastern Anatolia at a much earlier date, as shown by the numerous examples from Arslantepe and the site of Başur Höyük in the Turkish Upper Tigris region. This tradition, probably linked to the Southern Caucasus Kura-Araxes culture, was well established later in the whole Upper and Middle Euphrates valley in the course of the first half of the third millennium BC. At Arslantepe, the first examples are thought to date from 3300–3100 BC from the palace complex (a group of weapons from Period VIA, Late Chalcolithic 5); two more items have been found in a sort of public/communal building in the pastoralist settlement of Period VIB1 (the very beginning of Early Bronze I: estimated around 3100–3000 BC). These were immediately followed by the examples from the so-called Royal Tomb, dated originally to period VIB2, 2900–2800 BC, although there are reasons to think it very probably belongs to the same phase of the public building of period VIB1, or to the end of the period, at the transition to VIB2, and therefore no later than 2900 BC (Frangipane 2008).Therefore, although definite clues are scarce, it seems now possible that the ‘inspiration’ or ‘model’ for the La Pastora spear heads could have arrived in Valencina much earlier than previously thought. Interestingly, Matarrubilla and La Pastora share a common feature—unlike the monuments of the PP4-Montelirio/Montelirio sectors, they do not face sunrise (Hoskin 2001). They clearly sit at the heart of an area of intense activity, with the major tombs in the PP4-Montelirio/Montelirio sectors immediately around them. The difference in orientation (somewhat ‘heretical’ if compared to the early tholos horizon) and the differences in architecture (stone instead of sun-dried mud corbelling; the use of various kinds of stone but virtually no slate slabs; the very long corridor and very small chamber of La Pastora; and the presence of the enormous monolithic basin in Matarrubilla) suggest that they correspond to a different, perhaps later, ideological framework than the Montelirio tholos and tomb 10.042–10.049. That might simply reveal social or conceptual differences with other tholoi, but it could also be an important chronological clue. Was the non-solar orientation of La Pastora and Matarrubilla a deliberate attempt at challenging the earlier tradition reflected in the PP4-Montelirio/Montelirio sectors?

The activity dated at La Pastora clearly overlaps with the use of Bell Beaker ceramics at Valencina (*100%* *probable*). It is therefore not impossible that the very different kind of tholos that La Pastora represents could be a Beaker-related monument, although the construction of La Pastora very probably pre-dates the Beaker deposit at Calle Trabajadores N^os^ 14–18 (*85%* *probable*; Supplementary Table S1) and on general grounds such an association is unlikely. Users of Beakers across Western Europe variously re-used, respected or avoided pre-existing monumental constructions, but do not appear to have built things of this kind themselves. Within the lower Guadalquivir valley, Bell-Beaker re-use of earlier megalithic monuments is recorded at the nearby site of El Gandul (Lazarich González and Sánchez Andreu 2000). So could La Pastora, Matarrubilla and Ontiveros represent a pre-Beaker surge of activity at Valencina, perhaps representing a second push to reassert old lineage power or collective identity in the face of changes in the wider world beyond the lower Guadalquivir valley, along the lines suggested for other regions (Cardoso 2014; Vander Linden 2013)? Their location to the west of the easterly focus of the site could be significant from this perspective, placed in an arc, one might speculate, to protect an old space of funerary and sacred activity and tradition. Further afield, an analogous situation is offered by the probable date for the construction of Silbury Hill in northern Wiltshire, in southern England, around 2400 cal BC, right at the end of the Late Neolithic and just before or at the time of the first introduction of Beakers into Britain (Bayliss, McAvoy and Whittle 2007; Marshall et al. 2013).

As for non-megalithic monumentality, re-use of ditches seems to have continued well into the third quarter of the third millennium cal BC. The model for Calle Mariana de Pineda s/n estimates that Structure 30 (partly cutting a V-shaped ditch, Structure 1) was constructed in *2600*–*2495* *cal BC* (*95%* *probability*; *build: Structure 30 (Calle Mariana de Pineda)*; Fig. 25), probably in *2575*–*2565* *cal BC* (*3%* *probability*) or *2555*–*2505* *cal BC* (*65%* *probability*), thus making this date a *terminus ante quem* for when the ditch was in use. We have already speculated above whether ditch digging could also be related to changing and perhaps troubled times. The cutting of Structure 30 may, in any case, be seen as an attempt at continued use, or the re-use, of a pre-existing ditch.

In the scenario in which there was a gap or reduction in funerary activity for much of the 27th and 26th centuries BC, both Calle Mariana de Pineda s/n and Calle Trabajadores N^os^ 14–18 could hypothetically be seen as episodes of activity presaging and defining the final decline of the site. Perhaps it is significant that both those sectors could represent not just short-lived depositions but single events. The simple, single articulated burial from Parcela Municipal (*CNA*-*1499: 435/ROH437*; Fig. 51) and the burial from Structure 10 at La Gallega (Fig. 42) also fall in this later period of burial on the site. The deposit in Structure 30 at Calle Mariana de Pineda s/n appears to have been a simple, collective one, perhaps harking back to earlier traditions, while the Calle Trabajadores N^os^ 14–18 one is now clearly accompanied by evidence for scalping (if not butchery) of human bones in connection with large quantities of Bell Beaker pottery (Inacio et al. 2017). Is it just coincidence that the first clear evidence for such treatment of the dead so far discovered in Valencina, with possible implications of inter-personal violence, should fall chronologically at a point in the sequence where a major cultural change, in the form of decline—is taking place?

The question arises of whether these are traces of an exceptional event—involving the manipulation and deposition of selected human bones. Systematic bioarchaeological research on human bone material at Valencina only began a few years ago. Reliable data are still scarce and therefore other excavated human remains presenting signs of defleshing may have gone unnoticed. However, it is worth stressing that while the deposit of crania at Calle Trabajadores N^os^ 14–18 is located virtually at the centre of what some publications (Arteaga Mature and Cruz-Auñón Briones 1999a, b, 2001; Cruz-Auñón Briones and Arteaga Mature 1996, 1999) have deemed the ‘domestic’, ‘residential’ and ‘production’ area of the Valencina site, in none of the best known Copper Age settlements of southern Iberia, such as Zambujal, Los Millares or Marroquíes Bajos, has a deposit of this nature ever been found in association with domestic, residential or productive structures. The conjunction of elements at Calle Trabajadores N^os^ 14–18 (synchronicity of deaths, deposition of crania without full anatomical connection, defleshing marks and a large amount of Bell Beaker pottery) is unique, which lends credit to the possibility that such a deposit occurred at a time of stress or crisis during the period in which the collapse of the ideology and social order developing at the site since the 32nd century cal BC seems to have occurred.

The chronological model of Structure 30 at Calle Mariana de Pineda s/n, which post-dates the ditch in this sector, suggests that it was in use for *up to 50* *years* (*95%* *probability*; *use: Structure 30 (Calle Mariana de Pineda)*; Fig. 26), probably for *up to 25* *years* (*68%* *probability*) from *2600*–*2495* *cal BC* (*95%* *probability*; *build: Structure 30* (*Calle Mariana de Pineda*); Fig. 25), probably from *2575*–*2565* *cal BC* (*3%* *probability*) or *2555*–*2505* *cal BC* (*65%* *probability*) until the end of burial activity in *2580*–*2430* *cal BC* (*95%* *probability*; *end: Calle Mariana de Pineda*; Fig. 25), probably in *2570*–*2560* *cal BC* (*2%* *probability*) or *2525*–*2470* *cal BC* (*66%* *probability*). The tholos at La Pastora was probably constructed before Structure 30 at Calle Mariana de Pineda s/n (*64%* *probable*; Supplementary Table S1), but certainly continued to be used in some form afterwards (*100%* *probable*). Funerary activity at Calle Trabajadores N^os^ 14–18, however, probably occurred after both La Pastora (*85%* *probable*) and Structure 30 at Calle Mariana de Pineda (*100%* *probable*).

The most recent dates on human bone currently available for Valencina are those from Calle Trabajadores (Fig. 28), the burial from Structure 10 at La Gallega (Fig. 42), and human bone found in the atrium at La Pastora (Fig. 47). It should also be noted that copper working activity at Valencina probably continued into the last quarter of the third millennium cal BC. A sample of unidentified charcoal from a deposit which including copper-working waste from a ditch at Avenida de Andalucía (UBA-1024; Table 2; Fig. 42) falls into this period and, of the two samples from PP-Matarrubilla which clearly relate to a later episode of activity in this sector at this time (Fig. 40), one (Ua-32043) was a fragment of *Quercus ilex* charcoal recovered from within a lump of copper-working waste (Table 2).

This brings us to the question of the end of activity at Valencina, which is hard to pin down. Some radiocarbon dates do suggest some activity in the first centuries of the Early Bronze Age (c. 2200–1500 cal BC). Some of these dates were measured on imperfect samples—on samples of unknown material (Cerro de la Cabeza; Fig. 43; Table 2), or on unspecified charred material (Avenida de Andalucía; Fig. 42; Table 2). But others were obtained from material associated with copper smelting at the PP-Matarrubilla sector (Fig. 40) and at IES (Ua-32887; Table 3). This evidence suggests that the productive elements of the site may have survived the demise of the funerary complex and that sporadic activity, still poorly detected, may have taken place in the Early Bronze Age.

Indeed, from c. 2400 cal BC onwards, funerary activity at Valencina becomes difficult to discern at all. It is at around this time when, marking the end of the Copper Age funerary ideology and the beginning of the Bronze Age, primary individual inhumations in cists, small pits and *covachas* appear at the neighbouring sites of Jardín de Alá, Salteras, of SE-K and SE-B, Gerena (Hunt Ortíz et al. 2008), the Las Canteras tholos, at El Gandul (Hurtado Pérez and Amores Carredano 1984), and Carmona (Belén Deamos et al. 2015), all within a 35 km radius of Valencina. Thus, it seems conceivable that at the beginning of the 25th century cal BC, the social relations that had given rise to the funerary complex at Valencina around 700 years earlier were in crisis or threatened by dissolution. This is in line with the ‘twilight of enclosures’ (Valera 2015) and indeed with what has been termed the ‘collapse’ of the Copper Age way of life in southwest Iberia as a whole (Soares and Tavares 1998)—but that is a much wider question, which must be pursued elsewhere.

## Conclusions

Truly science-based archaeological research at Valencina only started about 10 years ago. Since the number of excavation records to be processed is enormous (in excess of 120 excavations have been carried out at the site), the future study of the site represents a major challenge. If the results of the excavation at PP4-Montelirio are extrapolated to the entire site, then there would be in excess of 40,000 prehistoric features in Valencina, of which only a small fraction have been investigated. However, great advances have been made in the last decade, with the study of collections of human and animal bone, and the archaeometric analysis of material culture. Our contribution in this paper is an attempt to introduce better temporal resolution to the research being undertaken.

The dating programme reported here provides the basis for a whole new kind of discussion regarding Valencina and, by implication, other southern Iberian Copper Age sites. The results of our 3-year sampling and dating effort, supported by several of the excavators who have worked extensively at the site in the last 15 years, offer numerous insights based on modelled estimates for timing, order and duration, but they also provide a further, more robust sense of the pattern and tempo of change at Valencina. On the basis of the results presented here, instead of activity spread across a full millennium, a more nuanced pattern of establishment, consolidation, ‘surge’ and decline can be suggested. Future research could now seek radically to refine such a picture. We do not understand the conditions under which Valencina was first selected as a focus for aggregation (or a place of residence); we do not know how quickly initial practices were adopted, nor how much time it took for significant areas of the total known site to come into use, nor the fine details of contemporaneous use of different areas and sectors. We do not yet grasp the circumstances that led to the showy new mortuary practices associated with the ‘early’ tholos horizon, or what conditions led to the construction of different (perhaps ‘heretical’), very large tholoi later on. Were these purely local conditions, or did they relate to the region around or beyond? We do not know the full extent or density of distributions of tholoi within the complex as a whole. We know very little—next to nothing—about the temporality of the segments of huge ditches that have been found throughout the site, and in fact, it is still not known whether those segments ever formed full enclosures, and what those enclosures may have been built for. And indeed we do not yet definitively know whether it is more appropriate to call Valencina a ‘village’, an ‘aggregation’ or a ‘place of assembly’.

If questions of this kind remain for Valencina, imagine the wider set of problems facing an enhanced understanding of the Copper Age in southern Iberia as a whole. Not only do we await the full publication of some other important sites, mentioned through this paper, but we also need their individual chronologies to be refined. The trajectory of change may well have varied between major sites and between regions. In conclusion, however, the results presented here seem to us to support a picture of fluid, dynamic and ultimately unstable social differentiation, rather than any model of incipient state formation or the emergence of class society. It is not better chronology alone that will resolve this great issue, but there seems to be little hope of settling it without a better grasp of the timing, duration and tempo of change, made possible by formal modelling.

## Electronic supplementary material

Below is the link to the electronic supplementary material.

Supplementary material 1 (DOCX 22 kb)
