# Supplementary material for: Assembling the Dead, Gathering the Living: Radiocarbon Dating and Bayesian Modelling for Copper Age Valencina de la Concepción (Seville, Spain)
Source: J World Prehist. 2018 May 19;31(2):179–313. doi: 10.1007/s10963-018-9114-2 (PMC5984651; doi:10.1007/s10963-018-9114-2)
Supplement: Supplementary file 1 — Supplementary material 1 (DOCX 22 kb) [file 10963_2018_9114_MOESM1_ESM.docx]

Supplementary Table 1. Probabilities of the order of key parameters relating to funerary practice at Valencina de la Concepción. The table gives the probability that the parameter down the left-hand column is earlier than the parameter along the top row. For example, the probability that *start: La Huera* is earlier than *start: Structure 28 (Calle Dinamarca)* is *71%*.

|  | ***start: La Huera*** | ***end: La Huera main use*** | ***start: Structure 5 (Calle Dinamarca)*** | ***end: Structure 5 (Calle Dinamarca)*** | ***start: Structure 28 (Calle Dinamarca)*** | ***end: Structure 28 (Calle Dinamarca)*** | **start: Structure 64 (IES)** | ***end: Structure 64 (IES)*** | ***start: La Alcazaba*** | ***end: La Alcazaba*** | ***start: El Algarrobillo*** | ***end: El Algarrobillo*** | ***start: Structure 10.031 (PP4: Montelirio)*** | ***end: Structure 10.031 (PP4: Montelirio)*** | ***start: Structure 10.042 (PP4: Montelirio)*** | ***end: Structure 10.042 (PP4: Montelirio)*** | ***start: Structure 10.071 (PP4: Montelirio)*** | ***end: Structure 10.071 (PP4: Montelirio)*** | ***start: Montelirio Tholos*** | ***end: Montelirio Tholos*** | ***start: Cerro de la Cabeza*** | ***end: Cerro de la Cabeza*** | ***start: La Pastora*** | ***end: La Pastora*** | ***build: Structure 30 (Calle Mariana de Pineda)*** | ***end: Calle Mariana de Pineda*** | ***start: Calle Trabajadores*** | ***end: Calle Trabajadores*** |
| --- | --- | --- | --- | --- | --- | --- | --- | --- | --- | --- | --- | --- | --- | --- | --- | --- | --- | --- | --- | --- | --- | --- | --- | --- | --- | --- | --- | --- |
| ***start: La Huera*** | - | 1.00 | 1.00 | 1.00 | 0.71 | 0.98 | 1.00 | 1.00 | 0.99 | 1.00 | 0.97 | 1.00 | 0.78 | 1.00 | 0.70 | 1.00 | 0.96 | 1.00 | 1.00 | 1.00 | 0.87 | 1.00 | 1.00 | 1.00 | 1.00 | 1.00 | 1.00 | 1.00 |
| ***end: La Huera main use*** |  | - | 0.44 | 1.00 | 0.00 | 0.22 | 0.81 | 1.00 | 0.87 | 1.00 | 0.68 | 1.00 | 0.64 | 1.00 | 0.31 | 1.00 | 0.69 | 1.00 | 1.00 | 1.00 | 0.55 | 1.00 | 1.00 | 1.00 | 1.00 | 1.00 | 1.00 | 1.00 |
| ***start: Structure 5 (Calle Dinamarca)*** |  |  | - | 1.00 | 0.00 | 0.24 | 0.87 | 1.00 | 0.91 | 1.00 | 0.71 | 1.00 | 0.65 | 1.00 | 0.34 | 1.00 | 0.73 | 1.00 | 1.00 | 1.00 | 0.59 | 1.00 | 1.00 | 1.00 | 1.00 | 1.00 | 1.00 | 1.00 |
| ***end: Structure 5 (Calle Dinamarca)*** |  |  |  | - | 0.00 | 0.12 | 0.04 | 0.99 | 0.02 | 0.50 | 0.30 | 1.00 | 0.53 | 1.00 | 0.03 | 0.98 | 0.04 | 0.94 | 0.95 | 1.00 | 0.15 | 0.99 | 1.00 | 1.00 | 1.00 | 1.00 | 1.00 | 1.00 |
| ***start: Structure 28 (Calle Dinamarca)*** |  |  |  |  | - | 0.95 | 1.00 | 1.00 | 0.99 | 1.00 | 0.95 | 1.00 | 0.77 | 1.00 | 0.67 | 1.00 | 0.95 | 1.00 | 1.00 | 1.00 | 0.84 | 1.00 | 1.00 | 1.00 | 1.00 | 1.00 | 1.00 | 1.00 |
| ***end: Structure 28 (Calle Dinamarca)*** |  |  |  |  |  | - | 0.82 | 0.94 | 0.83 | 0.89 | 0.75 | 1.00 | 0.65 | 0.98 | 0.43 | 0.96 | 0.74 | 0.93 | 0.92 | 0.95 | 0.63 | 0.95 | 0.98 | 1.00 | 0.98 | 0.99 | 0.99 | 1.00 |
| ***start: Structure 64 (IES)*** |  |  |  |  |  |  | - | 1.00 | 0.63 | 0.96 | 0.53 | 1.00 | 0.60 | 1.00 | 0.18 | 0.99 | 0.45 | 0.99 | 0.98 | 1.00 | 0.40 | 0.99 | 1.00 | 1.00 | 1.00 | 1.00 | 1.00 | 1.00 |
| ***end: Structure 64 (IES)*** |  |  |  |  |  |  |  | - | 0.00 | 0.05 | 0.04 | 1.00 | 0.30 | 0.93 | 0.00 | 0.70 | 0.00 | 0.40 | 0.29 | 0.72 | 0.02 | 0.66 | 0.95 | 1.00 | 0.99 | 1.00 | 1.00 | 1.00 |
| ***start: La Alcazaba*** |  |  |  |  |  |  |  |  | - | 0.98 | 0.49 | 1.00 | 0.60 | 1.00 | 0.14 | 1.00 | 0.35 | 1.00 | 0.99 | 1.00 | 0.35 | 1.00 | 1.00 | 1.00 | 1.00 | 1.00 | 1.00 | 1.00 |
| ***end: La Alcazaba*** |  |  |  |  |  |  |  |  |  | - | 0.28 | 1.00 | 0.51 | 0.99 | 0.03 | 0.95 | 0.04 | 0.89 | 0.89 | 0.97 | 0.14 | 0.95 | 0.99 | 1.00 | 0.99 | 1.00 | 1.00 | 1.00 |
| ***start: El Algarrobillo*** |  |  |  |  |  |  |  |  |  |  | - | 1.00 | 0.58 | 0.99 | 0.22 | 0.96 | 0.42 | 0.89 | 0.89 | 0.99 | 0.39 | 0.96 | 1.00 | 1.00 | 1.00 | 1.00 | 1.00 | 1.00 |
| ***end: El Algarrobillo*** |  |  |  |  |  |  |  |  |  |  |  | - | 0.00 | 0.37 | 0.00 | 0.26 | 0.00 | 0.04 | 0.00 | 0.00 | 0.00 | 0.16 | 0.04 | 0.90 | 0.03 | 0.14 | 0.12 | 0.56 |
| ***start: Structure 10.031 (PP4: Montelirio)*** |  |  |  |  |  |  |  |  |  |  |  |  | - | 0.95 | 0.29 | 0.83 | 0.38 | 0.68 | 0.63 | 0.77 | 0.37 | 0.80 | 0.96 | 1.00 | 0.99 | 1.00 | 1.00 | 1.00 |
| ***end: Structure 10.031 (PP4: Montelirio)*** |  |  |  |  |  |  |  |  |  |  |  |  |  | - | 0.00 | 0.33 | 0.00 | 0.11 | 0.04 | 0.10 | 0.00 | 0.26 | 0.36 | 0.79 | 0.44 | 0.52 | 0.52 | 0.64 |
| ***start: Structure 10.042 (PP4: Montelirio)*** |  |  |  |  |  |  |  |  |  |  |  |  |  |  | - | 1.00 | 0.76 | 0.99 | 0.99 | 1.00 | 0.68 | 1.00 | 1.00 | 1.00 | 1.00 | 1.00 | 1.00 | 1.00 |
| ***end: Structure 10.042 (PP4: Montelirio)*** |  |  |  |  |  |  |  |  |  |  |  |  |  |  |  | - | 0.00 | 0.27 | 0.20 | 0.38 | 0.02 | 0.45 | 0.59 | 0.86 | 0.64 | 0.68 | 0.68 | 0.74 |
| ***start: Structure 10.071 (PP4: Montelirio)*** |  |  |  |  |  |  |  |  |  |  |  |  |  |  |  |  | - | 0.99 | 0.98 | 1.00 | 0.45 | 1.00 | 1.00 | 1.00 | 1.00 | 1.00 | 1.00 | 1.00 |
| ***end: Structure 10.071 (PP4: Montelirio)*** |  |  |  |  |  |  |  |  |  |  |  |  |  |  |  |  |  | - | 0.47 | 0.70 | 0.06 | 0.71 | 0.88 | 0.99 | 0.93 | 0.94 | 0.94 | 0.97 |
| ***start: Montelirio Tholos*** |  |  |  |  |  |  |  |  |  |  |  |  |  |  |  |  |  |  | - | 0.89 | 0.06 | 0.78 | 0.99 | 1.00 | 1.00 | 1.00 | 1.00 | 1.00 |
| ***end: Montelirio Tholos*** |  |  |  |  |  |  |  |  |  |  |  |  |  |  |  |  |  |  |  | - | 0.01 | 0.56 | 0.93 | 1.00 | 1.00 | 1.00 | 1.00 | 1.00 |
| ***start: Cerro de la Cabeza*** |  |  |  |  |  |  |  |  |  |  |  |  |  |  |  |  |  |  |  |  | - | 0.98 | 1.00 | 1.00 | 1.00 | 1.00 | 1.00 | 1.00 |
| ***end: Cerro de la Cabeza*** |  |  |  |  |  |  |  |  |  |  |  |  |  |  |  |  |  |  |  |  |  | - | 0.69 | 0.92 | 0.74 | 0.79 | 0.78 | 0.85 |
| ***start: La Pastora*** |  |  |  |  |  |  |  |  |  |  |  |  |  |  |  |  |  |  |  |  |  |  | - | 1.00 | 0.64 | 0.83 | 0.85 | 0.99 |
| ***end: La Pastora*** |  |  |  |  |  |  |  |  |  |  |  |  |  |  |  |  |  |  |  |  |  |  |  | - | 0.00 | 0.01 | 0.00 | 0.07 |
| ***build: Structure 30 (Calle Mariana de Pineda)*** |  |  |  |  |  |  |  |  |  |  |  |  |  |  |  |  |  |  |  |  |  |  |  |  | - | 0.84 | 0.87 | 1.00 |
| ***end: Calle Mariana de Pineda*** |  |  |  |  |  |  |  |  |  |  |  |  |  |  |  |  |  |  |  |  |  |  |  |  |  | - | 0.55 | 0.96 |
| ***start: Calle Trabajadores*** |  |  |  |  |  |  |  |  |  |  |  |  |  |  |  |  |  |  |  |  |  |  |  |  |  |  | - | 1.00 |
| ***end: Calle Trabajadores*** |  |  |  |  |  |  |  |  |  |  |  |  |  |  |  |  |  |  |  |  |  |  |  |  |  |  |  | - |
